# Supplementary material for: Efficient discovery of frequently co-occurring mutations in a sequence database with matrix factorization
Source: PLoS Comput Biol. 2025 Apr 24;21(4):e1012391. doi: 10.1371/journal.pcbi.1012391 (PMC12273922; doi:10.1371/journal.pcbi.1012391)
Supplement: S1 Table — (PDF) [file pcbi.1012391.s001.pdf]

Table S1. List of all identified co-mutations labeled by unique IDs.

ID	Co-mutation
C1	N501
C2	T478
C3	L452,T478
C4	S371,S373,K417,E484
C5	S371,K417,S477,T478,E484
C6	S371,K417,N440,S477,T478
C7	K417,T478,E484,Q498,Y505
C8	G339,S371,S373,S375,K417,S477,T478
C9	G339,R346,S371,S373,S375,G496,N501
C10	S477,T478,E484,Q493,G496,Q498,N501,Y505
C11	G339,S477,T478,E484,Q493,Q498,N501,Y505
C12	K417,N440,S477,T478,E484,Q493,Q498,N501,Y505
C13	G339,S371,S373,S375,K417,N440,G446,S477,T478
C14	S371,D405,R408,K417,N440,L452,S477,E484,N501
C15	S371,S373,S375,K417,N440,G446,S477,T478,E484,G496,N501
C16	G339,S371,S373,S375,S477,T478,Q493,G496,Q498,N501,Y505
C17	G339,K417,N440,G446,S477,T478,E484,Q493,Q498,N501,Y505
C18	G339,S371,S373,S375,K417,N440,G446,T478,G496,Q498,Y505
C19	G339,R346,S371,S373,S375,S477,E484,Q493,G496,Q498,Y505
C20	G339,S371,S373,S375,T376,D405,R408,K417,N440,L452,E484,N501
C21	G339,S371,S373,S375,T376,D405,R408,K417,N440,S477,T478,E484,N501
C22	G339,S371,S373,S375,T376,D405,R408,K417,S477,T478,E484,Q498,Y505
C23	G339,S371,S373,S375,N440,G446,S477,T478,E484,Q493,G496,Q498,Y505
C24	G339,S371,S373,S375,T376,D405,R408,K417,N440,S477,T478,Q498,N501,Y505
C25	G339,S371,S373,S375,K417,N440,G446,S477,E484,Q493,G496,Q498,N501,Y505
C26	G339,R346,S371,S373,S375,K417,N440,G446,S477,T478,E484,Q493,G496,Q498,Y505
C27	G339,S371,S373,S375,T376,D405,R408,K417,N440,S477,T478,E484,Q493,Q498,N501,Y505
C28	G339,S371,S373,S375,T376,D405,R408,K417,N440,L452,T478,E484,F486,Q498,N501,Y505
C29	G339,S371,S373,S375,T376,D405,R408,K417,N440,S477,T478,E484,F486,Q498,N501,Y505
C30	G339,S371,S373,S375,T376,D405,R408,K417,N440,L452,S477,T478,E484,F486,Q498,Y505
C31	D405
C32	D405,E484,N501
C33	D405,K417,N440,E484,N501
C34	D405,K417,N440,L452,S477,E484,N501
C35	D405,K417,N440,S477,E484,N501
C36	D405,K417,N440,T478,E484,F486,Q498,N501,Y505
C37	D405,K417,N440,T478,E484,F486,Q498,Y505
C38	D405,K417,N440,T478,E484,N501
C39	D405,K417,N440,T478,E484,Q498,N501,Y505
C40	D405,K417,N440,T478,Q498,N501,Y505

C41	D405,K417,S477,E484,N501
C42	D405,K417,T478,E484,Q498,Y505
C43	D405,L452
C44	D405,L452,S477,E484,N501
C45	D405,L452,T478
C46	D405,N440,L452,S477,E484,N501
C47	D405,N440,S477,E484,N501
C48	D405,N501
C49	D405,R408,K417
C50	D405,R408,K417,L452,S477,E484,N501
C51	D405,R408,K417,N440
C52	D405,R408,K417,N440,E484,N501
C53	D405,R408,K417,N440,L452
C54	D405,R408,K417,N440,L452,E484,N501
C55	D405,R408,K417,N440,L452,S477,E484,N501
C56	D405,R408,K417,N440,L452,S477,T478,E484,Q498,Y505
C57	D405,R408,K417,N440,L452,T478,E484,Q498,N501,Y505
C58	D405,R408,K417,N440,S477,E484,N501
C59	D405,R408,K417,N440,S477,T478,E484,N501
C60	D405,R408,K417,N440,S477,T478,E484,Q493,Q498,N501,Y505
C61	D405,R408,K417,N440,S477,T478,E484,Q498,N501,Y505
C62	D405,R408,K417,N440,S477,T478,E484,Q498,Y505
C63	D405,R408,K417,N440,S477,T478,Q498,N501,Y505
C64	D405,R408,K417,N440,T478,E484,Q498,N501,Y505
C65	D405,R408,K417,S477,E484,N501
C66	D405,R408,K417,S477,T478,E484,Q498,Y505
C67	D405,R408,N440,S477,E484,N501
C68	D405,R408,S477,E484,N501
C69	D405,S477,E484,N501
C70	D405,T478
C71	E484
C72	E484,F486
C73	E484,F486,N501
C74	E484,F486,Q498
C75	E484,F486,Q498,N501
C76	E484,F486,Q498,N501,Y505
C77	E484,F486,Q498,Y505
C78	E484,F486,Y505
C79	E484,G496
C80	E484,G496,N501
C81	E484,G496,Q498,N501
C82	E484,G496,Q498,N501,Y505
C83	E484,N501

C84	E484,N501,Y505
C85	E484,Q493
C86	E484,Q493,G496
C87	E484,Q493,G496,N501
C88	E484,Q493,G496,N501,Y505
C89	E484,Q493,G496,Q498
C90	E484,Q493,G496,Q498,N501
C91	E484,Q493,G496,Q498,N501,Y505
C92	E484,Q493,G496,Q498,Y505
C93	E484,Q493,G496,Y505
C94	E484,Q493,N501
C95	E484,Q493,N501,Y505
C96	E484,Q493,Q498
C97	E484,Q493,Q498,N501
C98	E484,Q493,Q498,N501,Y505
C99	E484,Q493,Q498,Y505
C100	E484,Q493,Y505
C101	E484,Q498
C102	E484,Q498,N501
C103	E484,Q498,N501,Y505
C104	E484,Q498,Y505
C105	E484,Y505
C106	F486
C107	F486,N501
C108	F486,Q498,N501,Y505
C109	F486,Q498,Y505
C110	G339
C111	G339,D405,E484,N501
C112	G339,D405,K417,E484,N501
C113	G339,D405,K417,N440,E484,N501
C114	G339,D405,K417,N440,S477,T478,E484,N501
C115	G339,D405,K417,N440,S477,T478,E484,Q493,Q498,N501,Y505
C116	G339,D405,K417,N440,S477,T478,E484,Q498,N501,Y505
C117	G339,D405,K417,N440,S477,T478,E484,Q498,Y505
C118	G339,D405,K417,N440,S477,T478,Q498,N501,Y505
C119	G339,D405,K417,N440,T478,E484,Q498,N501,Y505
C120	G339,D405,K417,S477,T478,E484,F486,Q498,N501,Y505
C121	G339,D405,K417,S477,T478,E484,F486,Q498,Y505
C122	G339,D405,K417,S477,T478,E484,N501
C123	G339,D405,K417,S477,T478,E484,Q493,Q498,N501,Y505
C124	G339,D405,K417,S477,T478,E484,Q498,N501,Y505
C125	G339,D405,K417,S477,T478,E484,Q498,Y505
C126	G339,D405,K417,S477,T478,Q498,N501,Y505

C127	G339,D405,K417,T478,E484,F486,Q498,N501,Y505
C128	G339,D405,K417,T478,E484,Q498,N501,Y505
C129	G339,D405,L452,E484,N501
C130	G339,D405,L452,S477,T478,E484,F486,Q498,Y505
C131	G339,D405,L452,T478,E484,F486,Q498,N501,Y505
C132	G339,D405,N440,E484,N501
C133	G339,D405,N440,S477,T478,E484,N501
C134	G339,D405,N440,S477,T478,E484,Q493,Q498,N501,Y505
C135	G339,D405,N440,S477,T478,E484,Q498,N501,Y505
C136	G339,D405,N440,S477,T478,E484,Q498,Y505
C137	G339,D405,N440,S477,T478,Q498,N501,Y505
C138	G339,D405,N440,T478,E484,Q498,N501,Y505
C139	G339,D405,R408,E484,N501
C140	G339,D405,R408,K417
C141	G339,D405,R408,K417,E484,N501
C142	G339,D405,R408,K417,L452,E484,N501
C143	G339,D405,R408,K417,L452,S477,T478,E484,Q498,Y505
C144	G339,D405,R408,K417,L452,T478,E484,Q498,N501,Y505
C145	G339,D405,R408,K417,N440
C146	G339,D405,R408,K417,N440,E484,N501
C147	G339,D405,R408,K417,N440,L452
C148	G339,D405,R408,K417,N440,S477,T478,E484,N501
C149	G339,D405,R408,K417,N440,S477,T478,E484,Q493,Q498,N501,Y505
C150	G339,D405,R408,K417,N440,S477,T478,E484,Q498,N501,Y505
C151	G339,D405,R408,K417,N440,S477,T478,E484,Q498,Y505
C152	G339,D405,R408,K417,N440,S477,T478,Q498,N501,Y505
C153	G339,D405,R408,K417,N440,T478,E484,Q498,N501,Y505
C154	G339,D405,R408,K417,S477,T478,E484,N501
C155	G339,D405,R408,K417,S477,T478,E484,Q493,Q498,N501,Y505
C156	G339,D405,R408,K417,S477,T478,E484,Q498,N501,Y505
C157	G339,D405,R408,K417,S477,T478,E484,Q498,Y505
C158	G339,D405,R408,K417,S477,T478,Q498,N501,Y505
C159	G339,D405,R408,K417,T478,E484,Q498,N501,Y505
C160	G339,D405,R408,N440,E484,N501
C161	G339,D405,R408,N440,S477,T478,E484,N501
C162	G339,D405,R408,N440,S477,T478,E484,Q493,Q498,N501,Y505
C163	G339,D405,R408,N440,S477,T478,E484,Q498,N501,Y505
C164	G339,D405,R408,N440,S477,T478,E484,Q498,Y505
C165	G339,D405,R408,N440,S477,T478,Q498,N501,Y505
C166	G339,D405,R408,N440,T478,E484,Q498,N501,Y505
C167	G339,D405,R408,S477,T478,E484,N501
C168	G339,D405,R408,S477,T478,E484,Q493,Q498,N501,Y505
C169	G339,D405,R408,S477,T478,E484,Q498,N501,Y505

C170	G339,D405,R408,S477,T478,E484,Q498,Y505
C171	G339,D405,R408,S477,T478,Q498,N501,Y505
C172	G339,D405,R408,T478,E484,Q498,N501,Y505
C173	G339,D405,S477,T478,E484,F486,Q498,N501,Y505
C174	G339,D405,S477,T478,E484,N501
C175	G339,D405,S477,T478,E484,Q493,Q498,N501,Y505
C176	G339,D405,S477,T478,E484,Q498,N501,Y505
C177	G339,D405,S477,T478,E484,Q498,Y505
C178	G339,D405,S477,T478,Q498,N501,Y505
C179	G339,D405,T478,E484,Q498,N501,Y505
C180	G339,E484
C181	G339,E484,F486
C182	G339,E484,F486,Q498
C183	G339,E484,F486,Q498,N501
C184	G339,E484,F486,Q498,N501,Y505
C185	G339,E484,F486,Q498,Y505
C186	G339,E484,G496
C187	G339,E484,G496,Q498
C188	G339,E484,G496,Q498,N501
C189	G339,E484,G496,Q498,N501,Y505
C190	G339,E484,G496,Q498,Y505
C191	G339,E484,G496,Y505
C192	G339,E484,N501
C193	G339,E484,N501,Y505
C194	G339,E484,Q493
C195	G339,E484,Q493,G496
C196	G339,E484,Q493,G496,N501,Y505
C197	G339,E484,Q493,G496,Q498
C198	G339,E484,Q493,G496,Q498,N501
C199	G339,E484,Q493,G496,Q498,N501,Y505
C200	G339,E484,Q493,G496,Q498,Y505
C201	G339,E484,Q493,G496,Y505
C202	G339,E484,Q493,N501
C203	G339,E484,Q493,N501,Y505
C204	G339,E484,Q493,Q498
C205	G339,E484,Q493,Q498,N501
C206	G339,E484,Q493,Q498,N501,Y505
C207	G339,E484,Q493,Q498,Y505
C208	G339,E484,Q493,Y505
C209	G339,E484,Q498
C210	G339,E484,Q498,N501
C211	G339,E484,Q498,N501,Y505
C212	G339,E484,Q498,Y505

C213	G339,E484,Y505
C214	G339,F486,Q498,N501,Y505
C215	G339,F486,Q498,Y505
C216	G339,G446
C217	G339,G446,E484
C218	G339,G446,E484,G496
C219	G339,G446,E484,G496,N501
C220	G339,G446,E484,N501
C221	G339,G446,E484,Q493,G496,Q498
C222	G339,G446,E484,Q493,G496,Q498,N501,Y505
C223	G339,G446,E484,Q493,G496,Q498,Y505
C224	G339,G446,E484,Q493,Q498,N501,Y505
C225	G339,G446,E484,Q493,Q498,Y505
C226	G339,G446,G496
C227	G339,G446,G496,Q498
C228	G339,G446,G496,Q498,N501
C229	G339,G446,G496,Q498,Y505
C230	G339,G446,N501
C231	G339,G446,Q493,G496,Q498,N501,Y505
C232	G339,G446,Q493,G496,Q498,Y505
C233	G339,G446,Q493,Q498,N501,Y505
C234	G339,G446,Q498,N501
C235	G339,G446,S477,E484,G496,N501,Y505
C236	G339,G446,S477,E484,Q493
C237	G339,G446,S477,E484,Q493,G496,N501,Y505
C238	G339,G446,S477,E484,Q493,G496,Q498,N501
C239	G339,G446,S477,E484,Q493,G496,Q498,N501,Y505
C240	G339,G446,S477,E484,Y505
C241	G339,G446,S477,G496,N501,Y505
C242	G339,G446,S477,T478
C243	G339,G446,S477,T478,E484,G496,Y505
C244	G339,G446,S477,T478,E484,N501,Y505
C245	G339,G446,S477,T478,E484,Q493
C246	G339,G446,S477,T478,E484,Q493,G496,Q498
C247	G339,G446,S477,T478,E484,Q493,G496,Q498,Y505
C248	G339,G446,S477,T478,E484,Q493,G496,Y505
C249	G339,G446,S477,T478,E484,Q493,N501,Y505
C250	G339,G446,S477,T478,E484,Q493,Q498
C251	G339,G446,S477,T478,E484,Q493,Q498,N501
C252	G339,G446,S477,T478,E484,Q493,Q498,N501,Y505
C253	G339,G446,S477,T478,E484,Y505
C254	G339,G446,S477,T478,G496,Y505
C255	G339,G446,S477,T478,N501,Y505

C256	G339,G446,T478
C257	G339,G446,T478,E484,Q493,G496,Q498
C258	G339,G446,T478,E484,Q493,G496,Q498,Y505
C259	G339,G446,T478,E484,Q493,Q498
C260	G339,G446,T478,E484,Q493,Q498,N501,Y505
C261	G339,G446,T478,G496,Q498
C262	G339,G446,T478,G496,Q498,Y505
C263	G339,G446,T478,G496,Y505
C264	G339,G446,T478,Q493,G496,Q498,Y505
C265	G339,G446,T478,Q493,Q498,N501,Y505
C266	G339,G446,T478,Y505
C267	G339,G496
C268	G339,G496,N501
C269	G339,G496,Q498
C270	G339,G496,Q498,N501
C271	G339,G496,Q498,N501,Y505
C272	G339,G496,Q498,Y505
C273	G339,G496,Y505
C274	G339,K417
C275	G339,K417,E484
C276	G339,K417,E484,N501
C277	G339,K417,E484,Q493,G496,Q498,N501,Y505
C278	G339,K417,E484,Q493,G496,Q498,Y505
C279	G339,K417,E484,Q493,Q498,N501,Y505
C280	G339,K417,E484,Q498,N501,Y505
C281	G339,K417,E484,Q498,Y505
C282	G339,K417,G446,E484
C283	G339,K417,G446,E484,Q493,Q498,N501,Y505
C284	G339,K417,G446,Q493,Q498,N501,Y505
C285	G339,K417,G446,S477,E484,Q493,G496,Q498,N501,Y505
C286	G339,K417,G446,S477,T478
C287	G339,K417,G446,S477,T478,E484,Q493,G496,Q498,Y505
C288	G339,K417,G446,S477,T478,E484,Q493,Q498,N501,Y505
C289	G339,K417,G446,T478,G496,Q498,Y505
C290	G339,K417,G496,Q498,Y505
C291	G339,K417,N440
C292	G339,K417,N440,E484,N501
C293	G339,K417,N440,E484,Q493,Q498,N501,Y505
C294	G339,K417,N440,E484,Q498,N501
C295	G339,K417,N440,E484,Q498,N501,Y505
C296	G339,K417,N440,G446
C297	G339,K417,N440,G446,E484,Q493
C298	G339,K417,N440,G446,E484,Q493,Q498,N501,Y505

C299	G339,K417,N440,G446,N501,Y505
C300	G339,K417,N440,G446,Q493
C301	G339,K417,N440,G446,Q493,Q498,N501,Y505
C302	G339,K417,N440,G446,S477
C303	G339,K417,N440,G446,S477,E484,Q493,G496,Q498,N501,Y505
C304	G339,K417,N440,G446,S477,E484,Q493,Q498,N501,Y505
C305	G339,K417,N440,G446,S477,Q493,Q498,N501,Y505
C306	G339,K417,N440,G446,S477,T478
C307	G339,K417,N440,G446,S477,T478,E484
C308	G339,K417,N440,G446,S477,T478,E484,N501,Y505
C309	G339,K417,N440,G446,S477,T478,E484,Q493
C310	G339,K417,N440,G446,S477,T478,E484,Q493,G496,Q498,Y505
C311	G339,K417,N440,G446,S477,T478,E484,Q493,Q498
C312	G339,K417,N440,G446,S477,T478,E484,Q493,Q498,N501
C313	G339,K417,N440,G446,S477,T478,E484,Q493,Q498,Y505
C314	G339,K417,N440,G446,S477,T478,E484,Q493,Y505
C315	G339,K417,N440,G446,S477,T478,E484,Q498,N501,Y505
C316	G339,K417,N440,G446,S477,T478,N501
C317	G339,K417,N440,G446,S477,T478,N501,Y505
C318	G339,K417,N440,G446,S477,T478,Q493,Q498,N501,Y505
C319	G339,K417,N440,G446,S477,T478,Q498,N501,Y505
C320	G339,K417,N440,G446,S477,T478,Y505
C321	G339,K417,N440,G446,T478,E484,Q493,Q498,N501,Y505
C322	G339,K417,N440,G446,T478,G496,Q498,Y505
C323	G339,K417,N440,G446,T478,Q498
C324	G339,K417,N440,G446,T478,Q498,N501
C325	G339,K417,N440,Q493,Q498,N501,Y505
C326	G339,K417,N440,S477,E484,Q493,Q498,N501,Y505
C327	G339,K417,N440,S477,E484,Q498,N501,Y505
C328	G339,K417,N440,S477,T478
C329	G339,K417,N440,S477,T478,E484
C330	G339,K417,N440,S477,T478,E484,N501
C331	G339,K417,N440,S477,T478,E484,Q493
C332	G339,K417,N440,S477,T478,E484,Q493,N501,Y505
C333	G339,K417,N440,S477,T478,E484,Q493,Q498
C334	G339,K417,N440,S477,T478,E484,Q493,Q498,N501
C335	G339,K417,N440,S477,T478,E484,Q493,Q498,N501,Y505
C336	G339,K417,N440,S477,T478,E484,Q493,Q498,Y505
C337	G339,K417,N440,S477,T478,E484,Q498,N501
C338	G339,K417,N440,S477,T478,E484,Q498,N501,Y505
C339	G339,K417,N440,S477,T478,E484,Q498,Y505
C340	G339,K417,N440,S477,T478,Q493,Q498,N501,Y505
C341	G339,K417,N440,S477,T478,Q498,N501,Y505

C342	G339,K417,N440,T478,E484,Q498,N501,Y505
C343	G339,K417,N440,T478,Q498,Y505
C344	G339,K417,N501
C345	G339,K417,Q493,Q498,N501,Y505
C346	G339,K417,Q498,N501,Y505
C347	G339,K417,S477,E484,Q493,Q498,N501,Y505
C348	G339,K417,S477,E484,Q498,N501,Y505
C349	G339,K417,S477,T478
C350	G339,K417,S477,T478,E484,F486,Q498,N501,Y505
C351	G339,K417,S477,T478,E484,F486,Q498,Y505
C352	G339,K417,S477,T478,E484,N501
C353	G339,K417,S477,T478,E484,Q493
C354	G339,K417,S477,T478,E484,Q493,Q498,N501
C355	G339,K417,S477,T478,E484,Q493,Q498,N501,Y505
C356	G339,K417,S477,T478,E484,Q493,Q498,Y505
C357	G339,K417,S477,T478,E484,Q498,N501,Y505
C358	G339,K417,S477,T478,E484,Q498,Y505
C359	G339,K417,S477,T478,Q498,N501,Y505
C360	G339,K417,T478
C361	G339,K417,T478,E484,F486,Q498,N501,Y505
C362	G339,K417,T478,E484,Q498,N501,Y505
C363	G339,K417,T478,Q498,Y505
C364	G339,K417,Y505
C365	G339,L452
C366	G339,L452,E484
C367	G339,L452,E484,N501
C368	G339,L452,N501
C369	G339,L452,S477,T478,E484,F486,Q498,Y505
C370	G339,L452,T478
C371	G339,L452,T478,E484
C372	G339,L452,T478,E484,F486,Q498,N501,Y505
C373	G339,L452,T478,E484,Q498,N501,Y505
C374	G339,L452,T478,E484,Q498,Y505
C375	G339,L452,T478,Q498,N501,Y505
C376	G339,L452,T478,Q498,Y505
C377	G339,N440
C378	G339,N440,E484
C379	G339,N440,E484,N501
C380	G339,N440,E484,Q493,G496,Q498,N501,Y505
C381	G339,N440,E484,Q493,G496,Q498,Y505
C382	G339,N440,E484,Q493,Q498,N501,Y505
C383	G339,N440,E484,Q498,N501,Y505
C384	G339,N440,E484,Q498,Y505

C385	G339,N440,G446
C386	G339,N440,G446,E484,Q493,G496,Q498,N501,Y505
C387	G339,N440,G446,E484,Q493,G496,Q498,Y505
C388	G339,N440,G446,E484,Q493,N501,Y505
C389	G339,N440,G446,E484,Q493,Q498,N501,Y505
C390	G339,N440,G446,G496,Q498,Y505
C391	G339,N440,G446,N501,Y505
C392	G339,N440,G446,Q493
C393	G339,N440,G446,Q493,G496,Q498,N501,Y505
C394	G339,N440,G446,Q493,Q498
C395	G339,N440,G446,Q493,Q498,N501
C396	G339,N440,G446,Q493,Q498,N501,Y505
C397	G339,N440,G446,S477
C398	G339,N440,G446,S477,E484
C399	G339,N440,G446,S477,E484,Q493,G496,Q498
C400	G339,N440,G446,S477,E484,Q493,G496,Q498,N501
C401	G339,N440,G446,S477,E484,Q493,G496,Q498,N501,Y505
C402	G339,N440,G446,S477,E484,Q493,G496,Q498,Y505
C403	G339,N440,G446,S477,E484,Q493,Q498,N501,Y505
C404	G339,N440,G446,S477,T478
C405	G339,N440,G446,S477,T478,E484
C406	G339,N440,G446,S477,T478,E484,Q493
C407	G339,N440,G446,S477,T478,E484,Q493,G496,Q498
C408	G339,N440,G446,S477,T478,E484,Q493,G496,Q498,Y505
C409	G339,N440,G446,S477,T478,E484,Q493,N501,Y505
C410	G339,N440,G446,S477,T478,E484,Q493,Q498
C411	G339,N440,G446,S477,T478,E484,Q493,Q498,N501
C412	G339,N440,G446,S477,T478,E484,Q493,Q498,N501,Y505
C413	G339,N440,G446,S477,T478,E484,Q493,Q498,Y505
C414	G339,N440,G446,S477,T478,Q493,Q498,N501,Y505
C415	G339,N440,G446,T478
C416	G339,N440,G446,T478,E484,Q493,G496,Q498,Y505
C417	G339,N440,G446,T478,E484,Q493,Q498,N501
C418	G339,N440,G446,T478,E484,Q493,Q498,N501,Y505
C419	G339,N440,G446,T478,G496,Q498
C420	G339,N440,G446,T478,G496,Q498,Y505
C421	G339,N440,G446,T478,Q493,G496,Q498,Y505
C422	G339,N440,G446,T478,Q493,Q498,N501,Y505
C423	G339,N440,G446,T478,Q498,Y505
C424	G339,N440,G446,Y505
C425	G339,N440,G496
C426	G339,N440,G496,Q498,Y505
C427	G339,N440,N501

C428	G339,N440,N501,Y505
C429	G339,N440,Q493,G496
C430	G339,N440,Q493,G496,N501
C431	G339,N440,Q493,N501
C432	G339,N440,Q493,Q498,N501,Y505
C433	G339,N440,Q498,N501,Y505
C434	G339,N440,S477,E484,N501
C435	G339,N440,S477,E484,Q493,G496,Q498,N501
C436	G339,N440,S477,E484,Q493,G496,Q498,N501,Y505
C437	G339,N440,S477,E484,Q493,Q498,N501,Y505
C438	G339,N440,S477,E484,Q498,N501,Y505
C439	G339,N440,S477,E484,Q498,Y505
C440	G339,N440,S477,Q498,N501,Y505
C441	G339,N440,S477,T478
C442	G339,N440,S477,T478,E484
C443	G339,N440,S477,T478,E484,N501
C444	G339,N440,S477,T478,E484,Q493,G496,Q498
C445	G339,N440,S477,T478,E484,Q493,G496,Q498,Y505
C446	G339,N440,S477,T478,E484,Q493,Q498
C447	G339,N440,S477,T478,E484,Q493,Q498,N501
C448	G339,N440,S477,T478,E484,Q493,Q498,N501,Y505
C449	G339,N440,S477,T478,E484,Q493,Q498,Y505
C450	G339,N440,S477,T478,E484,Q498
C451	G339,N440,S477,T478,E484,Q498,N501
C452	G339,N440,S477,T478,E484,Q498,N501,Y505
C453	G339,N440,S477,T478,E484,Q498,Y505
C454	G339,N440,S477,T478,Q498
C455	G339,N440,S477,T478,Q498,N501
C456	G339,N440,S477,T478,Q498,N501,Y505
C457	G339,N440,T478
C458	G339,N440,T478,E484
C459	G339,N440,T478,E484,N501
C460	G339,N440,T478,E484,Q493,G496,Q498,Y505
C461	G339,N440,T478,E484,Q493,Q498,N501,Y505
C462	G339,N440,T478,E484,Q498
C463	G339,N440,T478,E484,Q498,N501
C464	G339,N440,T478,E484,Q498,N501,Y505
C465	G339,N440,T478,E484,Q498,Y505
C466	G339,N440,T478,G496,Q498
C467	G339,N440,T478,G496,Q498,Y505
C468	G339,N440,T478,N501
C469	G339,N440,T478,Q493,Q498,N501,Y505
C470	G339,N440,T478,Q498,N501,Y505

C471	G339,N440,T478,Q498,Y505
C472	G339,N440,Y505
C473	G339,N501
C474	G339,N501,Y505
C475	G339,Q493
C476	G339,Q493,G496
C477	G339,Q493,G496,N501
C478	G339,Q493,G496,N501,Y505
C479	G339,Q493,G496,Q498
C480	G339,Q493,G496,Q498,N501
C481	G339,Q493,G496,Q498,N501,Y505
C482	G339,Q493,G496,Q498,Y505
C483	G339,Q493,G496,Y505
C484	G339,Q493,N501
C485	G339,Q493,N501,Y505
C486	G339,Q493,Q498
C487	G339,Q493,Q498,N501
C488	G339,Q493,Q498,N501,Y505
C489	G339,Q493,Q498,Y505
C490	G339,Q493,Y505
C491	G339,Q498
C492	G339,Q498,N501
C493	G339,Q498,N501,Y505
C494	G339,Q498,Y505
C495	G339,R346
C496	G339,R346,E484
C497	G339,R346,E484,G496,Y505
C498	G339,R346,E484,Q493,G496
C499	G339,R346,E484,Q493,G496,Q498
C500	G339,R346,E484,Q493,G496,Q498,Y505
C501	G339,R346,E484,Q493,G496,Y505
C502	G339,R346,E484,Y505
C503	G339,R346,G446,E484,Q493,G496,Q498,Y505
C504	G339,R346,G446,G496,Q498
C505	G339,R346,G446,Q493,G496,Q498,Y505
C506	G339,R346,G446,S477,T478,E484,Q493,G496,Q498,Y505
C507	G339,R346,G446,S477,T478,E484,Q493,G496,Y505
C508	G339,R346,G446,S477,T478,E484,Y505
C509	G339,R346,G446,S477,T478,G496,Y505
C510	G339,R346,G446,T478,E484,Q493,G496,Q498
C511	G339,R346,G446,T478,E484,Q493,G496,Q498,Y505
C512	G339,R346,G446,T478,Q493,G496,Q498,Y505
C513	G339,R346,G496

C514	G339,R346,G496,N501
C515	G339,R346,G496,Q498
C516	G339,R346,G496,Q498,Y505
C517	G339,R346,K417,N440,G446,S477,T478,E484,Q493,G496,Q498,Y505
C518	G339,R346,N440,G446,E484,Q493,G496,Q498,Y505
C519	G339,R346,N440,G446,S477,T478
C520	G339,R346,N440,G446,S477,T478,E484,Q493,G496,Q498
C521	G339,R346,N440,G446,S477,T478,E484,Q493,G496,Q498,Y505
C522	G339,R346,N440,G446,S477,T478,E484,Q493,Q498,Y505
C523	G339,R346,N440,G446,T478,E484,Q493,G496,Q498,Y505
C524	G339,R346,N440,G446,Y505
C525	G339,R346,N440,S477,T478,E484,Q493,G496,Q498
C526	G339,R346,N440,S477,T478,E484,Q493,G496,Q498,Y505
C527	G339,R346,N440,Y505
C528	G339,R346,N501
C529	G339,R346,Q493
C530	G339,R346,Q493,G496,Q498,Y505
C531	G339,R346,Q493,G496,Y505
C532	G339,R346,Q498
C533	G339,R346,S371,S373,G496,N501
C534	G339,R346,S371,S373,K417,G446,Q493,G496,Q498,Y505
C535	G339,R346,S371,S373,K417,N440,G446,S477,T478,E484,Q493,G496,Q498,Y505
C536	G339,R346,S371,S373,K417,N440,G446,T478,Q498
C537	G339,R346,S371,S373,N501
C538	G339,R346,S371,S373,Q493,G496,Q498,Y505
C539	G339,R346,S371,S373,Q498
C540	G339,R346,S371,S373,S375
C541	G339,R346,S371,S373,S375,E484
C542	G339,R346,S371,S373,S375,E484,G496,Q498,Y505
C543	G339,R346,S371,S373,S375,E484,G496,Y505
C544	G339,R346,S371,S373,S375,E484,Q493,G496
C545	G339,R346,S371,S373,S375,E484,Q493,G496,Q498
C546	G339,R346,S371,S373,S375,E484,Q493,G496,Q498,Y505
C547	G339,R346,S371,S373,S375,E484,Q493,Q498
C548	G339,R346,S371,S373,S375,E484,Q493,Q498,Y505
C549	G339,R346,S371,S373,S375,E484,Q498,Y505
C550	G339,R346,S371,S373,S375,E484,Y505
C551	G339,R346,S371,S373,S375,G446
C552	G339,R346,S371,S373,S375,G446,E484,Q493,G496,Q498,Y505
C553	G339,R346,S371,S373,S375,G446,Q493,G496,Q498,Y505
C554	G339,R346,S371,S373,S375,G446,S477,T478
C555	G339,R346,S371,S373,S375,G446,S477,T478,E484,Q493,G496,Q498
C556	G339,R346,S371,S373,S375,G446,S477,T478,E484,Q493,G496,Q498,Y505

C557	G339,R346,S371,S373,S375,G496
C558	G339,R346,S371,S373,S375,G496,Q498
C559	G339,R346,S371,S373,S375,G496,Q498,Y505
C560	G339,R346,S371,S373,S375,G496,Y505
C561	G339,R346,S371,S373,S375,K417
C562	G339,R346,S371,S373,S375,K417,G446,E484
C563	G339,R346,S371,S373,S375,K417,G446,S477,T478,E484,Q493,G496,Q498,Y505
C564	G339,R346,S371,S373,S375,K417,N440
C565	G339,R346,S371,S373,S375,K417,N440,G446
C566	G339,R346,S371,S373,S375,K417,N440,G446,E484,Q493,G496,Q498,Y505
C567	G339,R346,S371,S373,S375,K417,N440,G446,Q493,G496,Q498,Y505
C568	G339,R346,S371,S373,S375,K417,N440,G446,S477
C569	G339,R346,S371,S373,S375,K417,N440,G446,S477,E484,Q493,G496,Q498,Y505
C570	G339,R346,S371,S373,S375,K417,N440,G446,S477,Q493,G496,Q498,Y505
C571	G339,R346,S371,S373,S375,K417,N440,G446,S477,T478
C572	G339,R346,S371,S373,S375,K417,N440,G446,S477,T478,E484
C573	G339,R346,S371,S373,S375,K417,N440,G446,S477,T478,E484,G496,Q498,Y505
C574	G339,R346,S371,S373,S375,K417,N440,G446,S477,T478,E484,Q493
C575	G339,R346,S371,S373,S375,K417,N440,G446,S477,T478,E484,Q493,G496
C576	G339,R346,S371,S373,S375,K417,N440,G446,S477,T478,E484,Q493,G496,Q498
C577	G339,R346,S371,S373,S375,K417,N440,G446,S477,T478,E484,Q493,G496,Y505
C578	G339,R346,S371,S373,S375,K417,N440,G446,S477,T478,E484,Q493,Q498,Y505
C579	G339,R346,S371,S373,S375,K417,N440,G446,S477,T478,E484,Q493,Y505
C580	G339,R346,S371,S373,S375,K417,N440,G446,S477,T478,E484,Q498,Y505
C581	G339,R346,S371,S373,S375,K417,N440,G446,S477,T478,E484,Y505
C582	G339,R346,S371,S373,S375,K417,N440,G446,S477,T478,G496,Q498,Y505
C583	G339,R346,S371,S373,S375,K417,N440,G446,S477,T478,Q493,G496,Q498,Y505
C584	G339,R346,S371,S373,S375,K417,N440,G446,S477,T478,Y505
C585	G339,R346,S371,S373,S375,K417,N440,G446,T478,E484,Q493,G496,Q498,Y505
C586	G339,R346,S371,S373,S375,K417,N440,S477,T478,E484
C587	G339,R346,S371,S373,S375,K417,N440,S477,T478,E484,G496,Q498,Y505
C588	G339,R346,S371,S373,S375,K417,N440,S477,T478,E484,Q493,G496,Q498,Y505
C589	G339,R346,S371,S373,S375,K417,N440,S477,T478,E484,Q493,Q498,Y505
C590	G339,R346,S371,S373,S375,K417,N440,S477,T478,E484,Q498,Y505
C591	G339,R346,S371,S373,S375,K417,S477,E484,Q493,G496,Q498,Y505
C592	G339,R346,S371,S373,S375,K417,S477,T478
C593	G339,R346,S371,S373,S375,K417,S477,T478,E484,Q493,G496,Q498,Y505
C594	G339,R346,S371,S373,S375,K417,S477,T478,E484,Q493,Q498,Y505
C595	G339,R346,S371,S373,S375,K417,S477,T478,E484,Q498,Y505
C596	G339,R346,S371,S373,S375,K417,Y505
C597	G339,R346,S371,S373,S375,N440,G446
C598	G339,R346,S371,S373,S375,N440,G446,Q493
C599	G339,R346,S371,S373,S375,N440,G446,Q493,G496,Q498,Y505

C600	G339,R346,S371,S373,S375,N440,G446,S477
C601	G339,R346,S371,S373,S375,N440,G446,S477,T478
C602	G339,R346,S371,S373,S375,N440,G446,S477,T478,E484
C603	G339,R346,S371,S373,S375,N440,G446,S477,T478,E484,Q493,G496
C604	G339,R346,S371,S373,S375,N440,G446,S477,T478,E484,Q493,G496,Q498
C605	G339,R346,S371,S373,S375,N440,G446,S477,T478,E484,Q493,G496,Q498,Y505
C606	G339,R346,S371,S373,S375,N440,G446,S477,T478,Q493,G496,Q498,Y505
C607	G339,R346,S371,S373,S375,N440,G446,Y505
C608	G339,R346,S371,S373,S375,N440,S477,T478,E484,Q493,G496,Q498,Y505
C609	G339,R346,S371,S373,S375,N440,S477,T478,E484,Q493,Q498,Y505
C610	G339,R346,S371,S373,S375,N440,S477,T478,E484,Q498,Y505
C611	G339,R346,S371,S373,S375,N501
C612	G339,R346,S371,S373,S375,Q493
C613	G339,R346,S371,S373,S375,Q493,G496
C614	G339,R346,S371,S373,S375,Q493,G496,Q498
C615	G339,R346,S371,S373,S375,Q493,G496,Q498,Y505
C616	G339,R346,S371,S373,S375,Q493,G496,Y505
C617	G339,R346,S371,S373,S375,Q493,Q498
C618	G339,R346,S371,S373,S375,Q493,Q498,Y505
C619	G339,R346,S371,S373,S375,Q493,Y505
C620	G339,R346,S371,S373,S375,Q498
C621	G339,R346,S371,S373,S375,Q498,Y505
C622	G339,R346,S371,S373,S375,S477
C623	G339,R346,S371,S373,S375,S477,E484
C624	G339,R346,S371,S373,S375,S477,E484,G496
C625	G339,R346,S371,S373,S375,S477,E484,G496,Q498,Y505
C626	G339,R346,S371,S373,S375,S477,E484,Q493
C627	G339,R346,S371,S373,S375,S477,E484,Q493,G496
C628	G339,R346,S371,S373,S375,S477,E484,Q493,G496,Q498
C629	G339,R346,S371,S373,S375,S477,E484,Q493,G496,Y505
C630	G339,R346,S371,S373,S375,S477,E484,Q493,Q498,Y505
C631	G339,R346,S371,S373,S375,S477,E484,Q493,Y505
C632	G339,R346,S371,S373,S375,S477,E484,Q498,Y505
C633	G339,R346,S371,S373,S375,S477,E484,Y505
C634	G339,R346,S371,S373,S375,S477,G496,Q498,Y505
C635	G339,R346,S371,S373,S375,S477,Q493,G496,Q498,Y505
C636	G339,R346,S371,S373,S375,S477,T478
C637	G339,R346,S371,S373,S375,S477,T478,E484,G496
C638	G339,R346,S371,S373,S375,S477,T478,E484,G496,Q498,Y505
C639	G339,R346,S371,S373,S375,S477,T478,E484,Q493
C640	G339,R346,S371,S373,S375,S477,T478,E484,Q493,G496
C641	G339,R346,S371,S373,S375,S477,T478,E484,Q493,G496,Q498
C642	G339,R346,S371,S373,S375,S477,T478,E484,Q493,G496,Q498,Y505

C643	G339,R346,S371,S373,S375,S477,T478,E484,Q493,G496,Y505
C644	G339,R346,S371,S373,S375,S477,T478,E484,Q493,Q498,Y505
C645	G339,R346,S371,S373,S375,S477,T478,E484,Q498,Y505
C646	G339,R346,S371,S373,S375,S477,T478,Q493,G496,Q498,Y505
C647	G339,R346,S371,S373,S375,S477,Y505
C648	G339,R346,S371,S373,S375,T478
C649	G339,R346,S371,S373,S375,T478,E484,G496,Y505
C650	G339,R346,S371,S373,S375,T478,E484,Q493,G496,Q498
C651	G339,R346,S371,S373,S375,T478,E484,Q493,G496,Q498,Y505
C652	G339,R346,S371,S373,S375,T478,E484,Q493,Q498,Y505
C653	G339,R346,S371,S373,S375,T478,E484,Q498,Y505
C654	G339,R346,S371,S373,S375,T478,G496,Q498
C655	G339,R346,S371,S373,S375,T478,Q493,G496,Q498,Y505
C656	G339,R346,S371,S373,S375,T478,Q498
C657	G339,R346,S371,S373,S375,Y505
C658	G339,R346,S371,S373,S477,E484,Q493,G496,Q498,Y505
C659	G339,R346,S371,S375,G496,N501
C660	G339,R346,S371,S375,K417,N440,G446,S477,T478,E484,Q493,G496,Q498,Y505
C661	G339,R346,S371,S375,S477,E484,Q493,G496,Q498,Y505
C662	G339,R346,S373,E484,Q493,G496,Q498,Y505
C663	G339,R346,S373,G496,N501
C664	G339,R346,S373,N440,G446,S477,T478,E484,Q493,G496,Q498,Y505
C665	G339,R346,S373,N501
C666	G339,R346,S373,Q493
C667	G339,R346,S373,S375,E484,Q493,G496,Q498
C668	G339,R346,S373,S375,G446,S477,T478,E484,Q493,G496,Q498,Y505
C669	G339,R346,S373,S375,G496,N501
C670	G339,R346,S373,S375,K417,N440,G446,S477,T478,E484,Q493,G496,Q498,Y505
C671	G339,R346,S373,S375,N440,G446,S477,T478,E484,Q493,G496,Q498,Y505
C672	G339,R346,S373,S375,N440,G446,T478,E484,Q493,G496,Q498
C673	G339,R346,S373,S375,N501
C674	G339,R346,S373,S375,S477,E484,Q493,G496,Q498,Y505
C675	G339,R346,S373,S375,S477,E484,Q498,Y505
C676	G339,R346,S373,S375,S477,T478,E484,Q498,Y505
C677	G339,R346,S373,S477,E484,Q493,G496,Q498,Y505
C678	G339,R346,S373,S477,E484,Q498,Y505
C679	G339,R346,S373,S477,T478,E484,Q498,Y505
C680	G339,R346,S375,E484,Q493,G496,Q498,Y505
C681	G339,R346,S375,G496,N501
C682	G339,R346,S375,K417,N440,G446,S477,T478
C683	G339,R346,S375,K417,N440,G446,T478,E484,Q493,G496,Q498,Y505
C684	G339,R346,S375,K417,S477,T478,E484,Q493,G496,Q498,Y505
C685	G339,R346,S375,N440,G446,S477,T478,E484,Q493,G496,Q498

C686	G339,R346,S375,N440,G446,S477,T478,E484,Q493,G496,Q498,Y505
C687	G339,R346,S375,N440,S477,T478,E484,Q493,G496,Q498,Y505
C688	G339,R346,S375,N501
C689	G339,R346,S375,S477
C690	G339,R346,S375,S477,E484,Q493,G496,Q498
C691	G339,R346,S375,S477,E484,Q493,G496,Q498,Y505
C692	G339,R346,S375,S477,T478,E484,Q493,G496,Q498,Y505
C693	G339,R346,S477
C694	G339,R346,S477,E484
C695	G339,R346,S477,E484,G496,Q498,Y505
C696	G339,R346,S477,E484,Q493,G496,Q498
C697	G339,R346,S477,E484,Q493,G496,Q498,Y505
C698	G339,R346,S477,E484,Q493,G496,Y505
C699	G339,R346,S477,E484,Q493,Q498,Y505
C700	G339,R346,S477,E484,Y505
C701	G339,R346,S477,G496,Y505
C702	G339,R346,S477,Q493,G496,Q498,Y505
C703	G339,R346,S477,T478,E484
C704	G339,R346,S477,T478,E484,G496,Q498,Y505
C705	G339,R346,S477,T478,E484,Q493,G496,Q498
C706	G339,R346,S477,T478,E484,Q493,G496,Q498,Y505
C707	G339,R346,S477,T478,E484,Q493,Q498,Y505
C708	G339,R346,S477,Y505
C709	G339,R346,T478
C710	G339,R346,T478,E484,G496,Y505
C711	G339,R346,T478,E484,Q493,G496
C712	G339,R346,T478,E484,Q493,G496,Q498
C713	G339,R346,T478,E484,Q493,G496,Q498,Y505
C714	G339,R346,T478,E484,Q493,G496,Y505
C715	G339,R346,T478,E484,Y505
C716	G339,R346,T478,Q493
C717	G339,R346,Y505
C718	G339,S371
C719	G339,S371,E484,F486,Q498,N501,Y505
C720	G339,S371,E484,F486,Q498,Y505
C721	G339,S371,E484,N501
C722	G339,S371,E484,Q493,Q498,N501,Y505
C723	G339,S371,E484,Q493,Q498,Y505
C724	G339,S371,E484,Q498,N501,Y505
C725	G339,S371,E484,Q498,Y505
C726	G339,S371,N501
C727	G339,S371,Q493,Q498,N501,Y505
C728	G339,S371,Q498,N501,Y505

C729	G339,S371,Q498,Y505
C730	G339,S371,S373
C731	G339,S371,S373,D405,E484,N501
C732	G339,S371,S373,D405,R408,K417,N440,E484,N501
C733	G339,S371,S373,D405,R408,K417,N440,S477,T478,E484,N501
C734	G339,S371,S373,D405,R408,K417,N440,S477,T478,E484,Q493,Q498,N501,Y505
C735	G339,S371,S373,D405,R408,K417,N440,S477,T478,E484,Q498,N501,Y505
C736	G339,S371,S373,D405,R408,K417,N440,S477,T478,E484,Q498,Y505
C737	G339,S371,S373,D405,R408,K417,N440,S477,T478,Q498,N501,Y505
C738	G339,S371,S373,D405,R408,K417,N440,T478,E484,Q498,N501,Y505
C739	G339,S371,S373,D405,R408,K417,S477,T478,E484,Q498,Y505
C740	G339,S371,S373,D405,S477,T478,E484,F486,Q498,N501,Y505
C741	G339,S371,S373,D405,S477,T478,E484,F486,Q498,Y505
C742	G339,S371,S373,D405,S477,T478,E484,N501
C743	G339,S371,S373,D405,S477,T478,E484,Q498,N501,Y505
C744	G339,S371,S373,D405,S477,T478,E484,Q498,Y505
C745	G339,S371,S373,D405,S477,T478,Q498,N501,Y505
C746	G339,S371,S373,D405,T478,E484,F486,Q498,N501,Y505
C747	G339,S371,S373,E484,F486,Q498,N501,Y505
C748	G339,S371,S373,E484,F486,Q498,Y505
C749	G339,S371,S373,E484,N501
C750	G339,S371,S373,E484,Q493,Q498
C751	G339,S371,S373,E484,Q493,Q498,N501,Y505
C752	G339,S371,S373,E484,Q493,Q498,Y505
C753	G339,S371,S373,E484,Q498,N501,Y505
C754	G339,S371,S373,E484,Q498,Y505
C755	G339,S371,S373,G446,Q493,G496,Q498,Y505
C756	G339,S371,S373,G496,N501
C757	G339,S371,S373,K417
C758	G339,S371,S373,K417,G446
C759	G339,S371,S373,K417,G446,G496,Q498,Y505
C760	G339,S371,S373,K417,G446,Q493,G496,Q498,N501,Y505
C761	G339,S371,S373,K417,N440,E484,N501
C762	G339,S371,S373,K417,N440,G446,Q498,N501
C763	G339,S371,S373,K417,N440,G446,S477,E484,Q493,G496,Q498,N501,Y505
C764	G339,S371,S373,K417,N440,G446,S477,T478
C765	G339,S371,S373,K417,N440,G446,S477,T478,E484,Q493,G496,Q498,Y505
C766	G339,S371,S373,K417,N440,G446,T478
C767	G339,S371,S373,K417,N440,G446,T478,G496,Q498,Y505
C768	G339,S371,S373,K417,N440,G446,T478,Q498
C769	G339,S371,S373,K417,N440,N501
C770	G339,S371,S373,K417,N440,S477,E484,Q493,Q498,N501,Y505
C771	G339,S371,S373,K417,N440,S477,T478

C772	G339,S371,S373,K417,N440,S477,T478,E484,N501
C773	G339,S371,S373,K417,N440,S477,T478,E484,Q493,Q498,N501,Y505
C774	G339,S371,S373,K417,N440,S477,T478,E484,Q493,Q498,Y505
C775	G339,S371,S373,K417,N440,S477,T478,E484,Q498,N501,Y505
C776	G339,S371,S373,K417,N440,S477,T478,E484,Q498,Y505
C777	G339,S371,S373,K417,N440,S477,T478,Q498,N501,Y505
C778	G339,S371,S373,K417,N440,T478,E484,Q498,N501,Y505
C779	G339,S371,S373,K417,N440,T478,N501
C780	G339,S371,S373,K417,N440,T478,Q498
C781	G339,S371,S373,K417,N440,T478,Q498,N501
C782	G339,S371,S373,K417,N440,T478,Q498,Y505
C783	G339,S371,S373,K417,N501
C784	G339,S371,S373,K417,Q493,Q498,N501,Y505
C785	G339,S371,S373,K417,Q498,N501,Y505
C786	G339,S371,S373,K417,Q498,Y505
C787	G339,S371,S373,K417,S477,T478
C788	G339,S371,S373,K417,S477,T478,E484,Q498,Y505
C789	G339,S371,S373,K417,T478
C790	G339,S371,S373,K417,T478,Q498
C791	G339,S371,S373,L452,E484,N501
C792	G339,S371,S373,L452,S477,T478,E484,F486,Q498,Y505
C793	G339,S371,S373,L452,S477,T478,E484,Q498,Y505
C794	G339,S371,S373,L452,T478,E484,F486,Q498,N501,Y505
C795	G339,S371,S373,L452,T478,E484,Q498,N501,Y505
C796	G339,S371,S373,N440,E484,N501
C797	G339,S371,S373,N440,G446,S477,E484,Q493,G496,Q498,N501,Y505
C798	G339,S371,S373,N440,G446,S477,T478
C799	G339,S371,S373,N440,G446,S477,T478,E484,Q493,G496,Q498,Y505
C800	G339,S371,S373,N440,G446,T478,G496,Q498,Y505
C801	G339,S371,S373,N440,G446,T478,Q498
C802	G339,S371,S373,N440,S477,E484,Q493,Q498,N501,Y505
C803	G339,S371,S373,N440,S477,T478
C804	G339,S371,S373,N440,S477,T478,E484,N501
C805	G339,S371,S373,N440,S477,T478,E484,Q493,Q498,N501,Y505
C806	G339,S371,S373,N440,S477,T478,E484,Q493,Q498,Y505
C807	G339,S371,S373,N440,S477,T478,E484,Q498,N501,Y505
C808	G339,S371,S373,N440,S477,T478,E484,Q498,Y505
C809	G339,S371,S373,N440,S477,T478,Q498,N501,Y505
C810	G339,S371,S373,N440,T478,E484,Q498,N501,Y505
C811	G339,S371,S373,N440,T478,Q498,Y505
C812	G339,S371,S373,N501
C813	G339,S371,S373,Q493,G496,Q498,N501,Y505
C814	G339,S371,S373,Q493,Q498

C815	G339,S371,S373,Q493,Q498,N501,Y505
C816	G339,S371,S373,Q493,Q498,Y505
C817	G339,S371,S373,Q498
C818	G339,S371,S373,Q498,N501,Y505
C819	G339,S371,S373,Q498,Y505
C820	G339,S371,S373,S375
C821	G339,S371,S373,S375,D405,K417,N440,E484,N501
C822	G339,S371,S373,S375,D405,K417,N440,S477,T478,E484,N501
C823	G339,S371,S373,S375,D405,K417,N440,S477,T478,E484,Q493,Q498,N501,Y505
C824	G339,S371,S373,S375,D405,K417,N440,S477,T478,E484,Q498,N501,Y505
C825	G339,S371,S373,S375,D405,K417,N440,S477,T478,E484,Q498,Y505
C826	G339,S371,S373,S375,D405,K417,N440,S477,T478,Q498,N501,Y505
C827	G339,S371,S373,S375,D405,K417,N440,T478,E484,Q498,N501,Y505
C828	G339,S371,S373,S375,D405,K417,S477,T478,E484,Q498,Y505
C829	G339,S371,S373,S375,D405,R408,K417,L452,E484,N501
C830	G339,S371,S373,S375,D405,R408,K417,L452,S477,T478,E484,Q498,Y505
C831	G339,S371,S373,S375,D405,R408,K417,L452,T478,E484,Q498,N501,Y505
C832	G339,S371,S373,S375,D405,R408,K417,N440,E484,N501
C833	G339,S371,S373,S375,D405,R408,K417,N440,L452,E484,N501
C834	G339,S371,S373,S375,D405,R408,K417,N440,L452,S477,T478,E484,Q498,Y505
C835	G339,S371,S373,S375,D405,R408,K417,N440,L452,T478,E484,Q498,N501,Y505
C836	G339,S371,S373,S375,D405,R408,K417,N440,S477,T478,E484,N501
C837	G339,S371,S373,S375,D405,R408,K417,N440,S477,T478,E484,Q493,Q498,N501,Y505
C838	G339,S371,S373,S375,D405,R408,K417,N440,S477,T478,E484,Q498,N501,Y505
C839	G339,S371,S373,S375,D405,R408,K417,N440,S477,T478,E484,Q498,Y505
C840	G339,S371,S373,S375,D405,R408,K417,N440,S477,T478,Q498,N501,Y505
C841	G339,S371,S373,S375,D405,R408,K417,N440,T478,E484,Q498,N501,Y505
C842	G339,S371,S373,S375,D405,R408,K417,S477,T478,E484,N501
C843	G339,S371,S373,S375,D405,R408,K417,S477,T478,E484,Q493,Q498,N501,Y505
C844	G339,S371,S373,S375,D405,R408,K417,S477,T478,E484,Q498,N501,Y505
C845	G339,S371,S373,S375,D405,R408,K417,S477,T478,E484,Q498,Y505
C846	G339,S371,S373,S375,D405,R408,K417,S477,T478,Q498,N501,Y505
C847	G339,S371,S373,S375,E484
C848	G339,S371,S373,S375,E484,F486,Q498,N501,Y505
C849	G339,S371,S373,S375,E484,F486,Q498,Y505
C850	G339,S371,S373,S375,E484,G496
C851	G339,S371,S373,S375,E484,G496,N501
C852	G339,S371,S373,S375,E484,G496,Q498
C853	G339,S371,S373,S375,E484,G496,Q498,N501
C854	G339,S371,S373,S375,E484,G496,Q498,N501,Y505
C855	G339,S371,S373,S375,E484,G496,Q498,Y505
C856	G339,S371,S373,S375,E484,G496,Y505
C857	G339,S371,S373,S375,E484,N501

C858	G339,S371,S373,S375,E484,N501,Y505
C859	G339,S371,S373,S375,E484,Q493
C860	G339,S371,S373,S375,E484,Q493,G496
C861	G339,S371,S373,S375,E484,Q493,G496,N501
C862	G339,S371,S373,S375,E484,Q493,G496,Q498
C863	G339,S371,S373,S375,E484,Q493,G496,Q498,N501
C864	G339,S371,S373,S375,E484,Q493,G496,Q498,N501,Y505
C865	G339,S371,S373,S375,E484,Q493,G496,Q498,Y505
C866	G339,S371,S373,S375,E484,Q493,G496,Y505
C867	G339,S371,S373,S375,E484,Q493,N501
C868	G339,S371,S373,S375,E484,Q493,Q498
C869	G339,S371,S373,S375,E484,Q493,Q498,N501
C870	G339,S371,S373,S375,E484,Q493,Q498,N501,Y505
C871	G339,S371,S373,S375,E484,Q493,Q498,Y505
C872	G339,S371,S373,S375,E484,Q498
C873	G339,S371,S373,S375,E484,Q498,N501
C874	G339,S371,S373,S375,E484,Q498,N501,Y505
C875	G339,S371,S373,S375,E484,Q498,Y505
C876	G339,S371,S373,S375,E484,Y505
C877	G339,S371,S373,S375,G446
C878	G339,S371,S373,S375,G446,E484
C879	G339,S371,S373,S375,G446,E484,Q493,G496,Q498,N501,Y505
C880	G339,S371,S373,S375,G446,E484,Q493,G496,Q498,Y505
C881	G339,S371,S373,S375,G446,G496,Q498,Y505
C882	G339,S371,S373,S375,G446,Q493,G496,Q498,N501,Y505
C883	G339,S371,S373,S375,G446,Q493,G496,Q498,Y505
C884	G339,S371,S373,S375,G446,S477
C885	G339,S371,S373,S375,G446,S477,E484,Q493,G496,Q498
C886	G339,S371,S373,S375,G446,S477,E484,Q493,G496,Q498,N501,Y505
C887	G339,S371,S373,S375,G446,S477,E484,Q493,Q498,N501,Y505
C888	G339,S371,S373,S375,G446,S477,T478
C889	G339,S371,S373,S375,G446,S477,T478,E484,Q493,G496,Q498
C890	G339,S371,S373,S375,G446,S477,T478,E484,Q493,G496,Q498,Y505
C891	G339,S371,S373,S375,G446,S477,T478,E484,Q493,Q498,Y505
C892	G339,S371,S373,S375,G446,T478
C893	G339,S371,S373,S375,G446,T478,G496,Q498
C894	G339,S371,S373,S375,G446,T478,G496,Q498,Y505
C895	G339,S371,S373,S375,G446,T478,Q498,Y505
C896	G339,S371,S373,S375,G496
C897	G339,S371,S373,S375,G496,N501
C898	G339,S371,S373,S375,G496,N501,Y505
C899	G339,S371,S373,S375,G496,Q498
C900	G339,S371,S373,S375,G496,Q498,N501

C901	G339,S371,S373,S375,G496,Q498,N501,Y505
C902	G339,S371,S373,S375,G496,Q498,Y505
C903	G339,S371,S373,S375,G496,Y505
C904	G339,S371,S373,S375,K417
C905	G339,S371,S373,S375,K417,E484
C906	G339,S371,S373,S375,K417,E484,N501
C907	G339,S371,S373,S375,K417,E484,Q493,Q498,N501,Y505
C908	G339,S371,S373,S375,K417,E484,Q493,Q498,Y505
C909	G339,S371,S373,S375,K417,E484,Q498,N501,Y505
C910	G339,S371,S373,S375,K417,E484,Q498,Y505
C911	G339,S371,S373,S375,K417,G446
C912	G339,S371,S373,S375,K417,G446,E484
C913	G339,S371,S373,S375,K417,G446,E484,Q493,G496,Q498,N501,Y505
C914	G339,S371,S373,S375,K417,G446,E484,Q493,G496,Q498,Y505
C915	G339,S371,S373,S375,K417,G446,G496,Q498,Y505
C916	G339,S371,S373,S375,K417,G446,S477
C917	G339,S371,S373,S375,K417,G446,S477,E484,Q493,G496,Q498,N501,Y505
C918	G339,S371,S373,S375,K417,G446,S477,E484,Q493,Q498,N501,Y505
C919	G339,S371,S373,S375,K417,G446,S477,T478
C920	G339,S371,S373,S375,K417,G446,S477,T478,E484,Q493,G496,Q498,Y505
C921	G339,S371,S373,S375,K417,G446,S477,T478,E484,Q493,Q498,Y505
C922	G339,S371,S373,S375,K417,G446,T478
C923	G339,S371,S373,S375,K417,G446,T478,G496,Q498,Y505
C924	G339,S371,S373,S375,K417,G446,T478,Q498,Y505
C925	G339,S371,S373,S375,K417,G496,Q498,Y505
C926	G339,S371,S373,S375,K417,N440
C927	G339,S371,S373,S375,K417,N440,E484
C928	G339,S371,S373,S375,K417,N440,E484,N501
C929	G339,S371,S373,S375,K417,N440,E484,Q493
C930	G339,S371,S373,S375,K417,N440,E484,Q493,Q498,N501,Y505
C931	G339,S371,S373,S375,K417,N440,E484,Q493,Q498,Y505
C932	G339,S371,S373,S375,K417,N440,E484,Q498
C933	G339,S371,S373,S375,K417,N440,E484,Q498,N501
C934	G339,S371,S373,S375,K417,N440,E484,Q498,N501,Y505
C935	G339,S371,S373,S375,K417,N440,E484,Q498,Y505
C936	G339,S371,S373,S375,K417,N440,G446
C937	G339,S371,S373,S375,K417,N440,G446,E484,Q493
C938	G339,S371,S373,S375,K417,N440,G446,E484,Q493,G496,Q498,N501,Y505
C939	G339,S371,S373,S375,K417,N440,G446,E484,Q493,G496,Q498,Y505
C940	G339,S371,S373,S375,K417,N440,G446,G496
C941	G339,S371,S373,S375,K417,N440,G446,G496,Q498
C942	G339,S371,S373,S375,K417,N440,G446,G496,Q498,Y505
C943	G339,S371,S373,S375,K417,N440,G446,N501,Y505

C944	G339,S371,S373,S375,K417,N440,G446,Q493,G496
C945	G339,S371,S373,S375,K417,N440,G446,Q493,G496,Q498,N501,Y505
C946	G339,S371,S373,S375,K417,N440,G446,Q493,G496,Q498,Y505
C947	G339,S371,S373,S375,K417,N440,G446,S477
C948	G339,S371,S373,S375,K417,N440,G446,S477,E484
C949	G339,S371,S373,S375,K417,N440,G446,S477,E484,G496,Q498,N501,Y505
C950	G339,S371,S373,S375,K417,N440,G446,S477,E484,N501,Y505
C951	G339,S371,S373,S375,K417,N440,G446,S477,E484,Q493
C952	G339,S371,S373,S375,K417,N440,G446,S477,E484,Q493,G496
C953	G339,S371,S373,S375,K417,N440,G446,S477,E484,Q493,G496,Q498
C954	G339,S371,S373,S375,K417,N440,G446,S477,E484,Q493,G496,Q498,N501
C955	G339,S371,S373,S375,K417,N440,G446,S477,E484,Q493,G496,Q498,Y505
C956	G339,S371,S373,S375,K417,N440,G446,S477,E484,Q493,G496,Y505
C957	G339,S371,S373,S375,K417,N440,G446,S477,E484,Q493,Q498,N501,Y505
C958	G339,S371,S373,S375,K417,N440,G446,S477,E484,Q493,Y505
C959	G339,S371,S373,S375,K417,N440,G446,S477,E484,Q498,N501,Y505
C960	G339,S371,S373,S375,K417,N440,G446,S477,G496,Q498,N501,Y505
C961	G339,S371,S373,S375,K417,N440,G446,S477,N501,Y505
C962	G339,S371,S373,S375,K417,N440,G446,S477,Q493,G496,Q498,N501,Y505
C963	G339,S371,S373,S375,K417,N440,G446,S477,T478,E484
C964	G339,S371,S373,S375,K417,N440,G446,S477,T478,E484,G496,Q498,Y505
C965	G339,S371,S373,S375,K417,N440,G446,S477,T478,E484,Q493
C966	G339,S371,S373,S375,K417,N440,G446,S477,T478,E484,Q493,G496
C967	G339,S371,S373,S375,K417,N440,G446,S477,T478,E484,Q493,G496,Q498
C968	G339,S371,S373,S375,K417,N440,G446,S477,T478,E484,Q493,G496,Q498,Y505
C969	G339,S371,S373,S375,K417,N440,G446,S477,T478,E484,Q493,G496,Y505
C970	G339,S371,S373,S375,K417,N440,G446,S477,T478,E484,Q493,Q498,Y505
C971	G339,S371,S373,S375,K417,N440,G446,S477,T478,E484,Q498,Y505
C972	G339,S371,S373,S375,K417,N440,G446,S477,T478,E484,Y505
C973	G339,S371,S373,S375,K417,N440,G446,S477,T478,Q493,G496,Q498,Y505
C974	G339,S371,S373,S375,K417,N440,G446,S477,T478,Y505
C975	G339,S371,S373,S375,K417,N440,G446,S477,Y505
C976	G339,S371,S373,S375,K417,N440,G446,T478
C977	G339,S371,S373,S375,K417,N440,G446,T478,E484,Q493,G496,Q498,Y505
C978	G339,S371,S373,S375,K417,N440,G446,T478,G496
C979	G339,S371,S373,S375,K417,N440,G446,T478,G496,Q498
C980	G339,S371,S373,S375,K417,N440,G446,T478,G496,Y505
C981	G339,S371,S373,S375,K417,N440,G446,T478,Q498,Y505
C982	G339,S371,S373,S375,K417,N440,G446,T478,Y505
C983	G339,S371,S373,S375,K417,N440,G446,Y505
C984	G339,S371,S373,S375,K417,N440,L452,E484,N501
C985	G339,S371,S373,S375,K417,N440,L452,S477,T478,E484,F486,Q498,Y505
C986	G339,S371,S373,S375,K417,N440,L452,S477,T478,E484,Q498,Y505

C987	G339,S371,S373,S375,K417,N440,L452,T478,E484,F486,Q498,N501,Y505
C988	G339,S371,S373,S375,K417,N440,L452,T478,E484,Q498,N501,Y505
C989	G339,S371,S373,S375,K417,N440,N501
C990	G339,S371,S373,S375,K417,N440,N501,Y505
C991	G339,S371,S373,S375,K417,N440,Q493
C992	G339,S371,S373,S375,K417,N440,Q493,Q498,N501,Y505
C993	G339,S371,S373,S375,K417,N440,Q493,Q498,Y505
C994	G339,S371,S373,S375,K417,N440,Q498
C995	G339,S371,S373,S375,K417,N440,Q498,N501,Y505
C996	G339,S371,S373,S375,K417,N440,Q498,Y505
C997	G339,S371,S373,S375,K417,N440,S477
C998	G339,S371,S373,S375,K417,N440,S477,E484
C999	G339,S371,S373,S375,K417,N440,S477,E484,G496,Q498,N501,Y505
C1000	G339,S371,S373,S375,K417,N440,S477,E484,N501
C1001	G339,S371,S373,S375,K417,N440,S477,E484,Q493
C1002	G339,S371,S373,S375,K417,N440,S477,E484,Q493,G496,Q498,N501,Y505
C1003	G339,S371,S373,S375,K417,N440,S477,E484,Q493,N501,Y505
C1004	G339,S371,S373,S375,K417,N440,S477,E484,Q493,Q498
C1005	G339,S371,S373,S375,K417,N440,S477,E484,Q493,Q498,N501
C1006	G339,S371,S373,S375,K417,N440,S477,E484,Q493,Q498,N501,Y505
C1007	G339,S371,S373,S375,K417,N440,S477,E484,Q493,Q498,Y505
C1008	G339,S371,S373,S375,K417,N440,S477,E484,Q498,N501
C1009	G339,S371,S373,S375,K417,N440,S477,E484,Q498,N501,Y505
C1010	G339,S371,S373,S375,K417,N440,S477,E484,Q498,Y505
C1011	G339,S371,S373,S375,K417,N440,S477,N501
C1012	G339,S371,S373,S375,K417,N440,S477,Q493,Q498,N501,Y505
C1013	G339,S371,S373,S375,K417,N440,S477,Q498,N501,Y505
C1014	G339,S371,S373,S375,K417,N440,S477,Q498,Y505
C1015	G339,S371,S373,S375,K417,N440,S477,T478
C1016	G339,S371,S373,S375,K417,N440,S477,T478,E484
C1017	G339,S371,S373,S375,K417,N440,S477,T478,E484,F486,Q498,N501,Y505
C1018	G339,S371,S373,S375,K417,N440,S477,T478,E484,F486,Q498,Y505
C1019	G339,S371,S373,S375,K417,N440,S477,T478,E484,N501
C1020	G339,S371,S373,S375,K417,N440,S477,T478,E484,N501,Y505
C1021	G339,S371,S373,S375,K417,N440,S477,T478,E484,Q493
C1022	G339,S371,S373,S375,K417,N440,S477,T478,E484,Q493,G496,Q498,Y505
C1023	G339,S371,S373,S375,K417,N440,S477,T478,E484,Q493,Q498
C1024	G339,S371,S373,S375,K417,N440,S477,T478,E484,Q493,Q498,N501
C1025	G339,S371,S373,S375,K417,N440,S477,T478,E484,Q493,Q498,N501,Y505
C1026	G339,S371,S373,S375,K417,N440,S477,T478,E484,Q493,Q498,Y505
C1027	G339,S371,S373,S375,K417,N440,S477,T478,E484,Q493,Y505
C1028	G339,S371,S373,S375,K417,N440,S477,T478,E484,Q498
C1029	G339,S371,S373,S375,K417,N440,S477,T478,E484,Q498,N501

C1030	G339,S371,S373,S375,K417,N440,S477,T478,E484,Q498,N501,Y505
C1031	G339,S371,S373,S375,K417,N440,S477,T478,E484,Q498,Y505
C1032	G339,S371,S373,S375,K417,N440,S477,T478,E484,Y505
C1033	G339,S371,S373,S375,K417,N440,S477,T478,N501
C1034	G339,S371,S373,S375,K417,N440,S477,T478,N501,Y505
C1035	G339,S371,S373,S375,K417,N440,S477,T478,Q493,Q498,N501,Y505
C1036	G339,S371,S373,S375,K417,N440,S477,T478,Q493,Q498,Y505
C1037	G339,S371,S373,S375,K417,N440,S477,T478,Q498
C1038	G339,S371,S373,S375,K417,N440,S477,T478,Q498,N501
C1039	G339,S371,S373,S375,K417,N440,S477,T478,Q498,N501,Y505
C1040	G339,S371,S373,S375,K417,N440,S477,T478,Q498,Y505
C1041	G339,S371,S373,S375,K417,N440,S477,T478,Y505
C1042	G339,S371,S373,S375,K417,N440,T478
C1043	G339,S371,S373,S375,K417,N440,T478,E484
C1044	G339,S371,S373,S375,K417,N440,T478,E484,F486,Q498,N501,Y505
C1045	G339,S371,S373,S375,K417,N440,T478,E484,N501
C1046	G339,S371,S373,S375,K417,N440,T478,E484,N501,Y505
C1047	G339,S371,S373,S375,K417,N440,T478,E484,Q493,Q498,N501,Y505
C1048	G339,S371,S373,S375,K417,N440,T478,E484,Q498
C1049	G339,S371,S373,S375,K417,N440,T478,E484,Q498,N501
C1050	G339,S371,S373,S375,K417,N440,T478,E484,Q498,N501,Y505
C1051	G339,S371,S373,S375,K417,N440,T478,E484,Q498,Y505
C1052	G339,S371,S373,S375,K417,N440,T478,E484,Y505
C1053	G339,S371,S373,S375,K417,N440,T478,G496,Q498,Y505
C1054	G339,S371,S373,S375,K417,N440,T478,N501,Y505
C1055	G339,S371,S373,S375,K417,N440,T478,Q498
C1056	G339,S371,S373,S375,K417,N440,T478,Q498,N501,Y505
C1057	G339,S371,S373,S375,K417,N440,T478,Q498,Y505
C1058	G339,S371,S373,S375,K417,N440,T478,Y505
C1059	G339,S371,S373,S375,K417,N440,Y505
C1060	G339,S371,S373,S375,K417,Q493,Q498,N501,Y505
C1061	G339,S371,S373,S375,K417,Q493,Q498,Y505
C1062	G339,S371,S373,S375,K417,Q498,N501,Y505
C1063	G339,S371,S373,S375,K417,Q498,Y505
C1064	G339,S371,S373,S375,K417,S477
C1065	G339,S371,S373,S375,K417,S477,E484,G496,Q498,N501,Y505
C1066	G339,S371,S373,S375,K417,S477,E484,N501
C1067	G339,S371,S373,S375,K417,S477,E484,Q493
C1068	G339,S371,S373,S375,K417,S477,E484,Q493,G496,Q498,N501
C1069	G339,S371,S373,S375,K417,S477,E484,Q493,G496,Q498,N501,Y505
C1070	G339,S371,S373,S375,K417,S477,E484,Q493,Q498,N501,Y505
C1071	G339,S371,S373,S375,K417,S477,E484,Q498,N501,Y505
C1072	G339,S371,S373,S375,K417,S477,E484,Q498,Y505

C1073	G339,S371,S373,S375,K417,S477,Q498,N501,Y505
C1074	G339,S371,S373,S375,K417,S477,Q498,Y505
C1075	G339,S371,S373,S375,K417,S477,T478,E484
C1076	G339,S371,S373,S375,K417,S477,T478,E484,G496,Q498,Y505
C1077	G339,S371,S373,S375,K417,S477,T478,E484,N501
C1078	G339,S371,S373,S375,K417,S477,T478,E484,Q493
C1079	G339,S371,S373,S375,K417,S477,T478,E484,Q493,G496,Q498
C1080	G339,S371,S373,S375,K417,S477,T478,E484,Q493,G496,Q498,Y505
C1081	G339,S371,S373,S375,K417,S477,T478,E484,Q493,Q498,N501
C1082	G339,S371,S373,S375,K417,S477,T478,E484,Q493,Q498,N501,Y505
C1083	G339,S371,S373,S375,K417,S477,T478,E484,Q493,Q498,Y505
C1084	G339,S371,S373,S375,K417,S477,T478,E484,Q498
C1085	G339,S371,S373,S375,K417,S477,T478,E484,Q498,N501
C1086	G339,S371,S373,S375,K417,S477,T478,E484,Q498,N501,Y505
C1087	G339,S371,S373,S375,K417,S477,T478,E484,Q498,Y505
C1088	G339,S371,S373,S375,K417,S477,T478,E484,Y505
C1089	G339,S371,S373,S375,K417,S477,T478,Q498,N501
C1090	G339,S371,S373,S375,K417,S477,T478,Q498,N501,Y505
C1091	G339,S371,S373,S375,K417,S477,T478,Q498,Y505
C1092	G339,S371,S373,S375,K417,S477,T478,Y505
C1093	G339,S371,S373,S375,K417,T478
C1094	G339,S371,S373,S375,K417,T478,E484,Q498,N501
C1095	G339,S371,S373,S375,K417,T478,E484,Q498,N501,Y505
C1096	G339,S371,S373,S375,K417,T478,E484,Q498,Y505
C1097	G339,S371,S373,S375,K417,T478,G496,Q498
C1098	G339,S371,S373,S375,K417,T478,G496,Q498,Y505
C1099	G339,S371,S373,S375,K417,T478,Q498
C1100	G339,S371,S373,S375,K417,T478,Q498,Y505
C1101	G339,S371,S373,S375,K417,Y505
C1102	G339,S371,S373,S375,L452
C1103	G339,S371,S373,S375,L452,E484
C1104	G339,S371,S373,S375,L452,E484,N501
C1105	G339,S371,S373,S375,L452,N501
C1106	G339,S371,S373,S375,L452,S477,T478,E484
C1107	G339,S371,S373,S375,L452,S477,T478,E484,Q498,Y505
C1108	G339,S371,S373,S375,L452,S477,T478,Q498,Y505
C1109	G339,S371,S373,S375,L452,T478
C1110	G339,S371,S373,S375,L452,T478,E484
C1111	G339,S371,S373,S375,L452,T478,E484,Q498,N501,Y505
C1112	G339,S371,S373,S375,L452,T478,E484,Q498,Y505
C1113	G339,S371,S373,S375,L452,T478,Q498
C1114	G339,S371,S373,S375,L452,T478,Q498,N501
C1115	G339,S371,S373,S375,L452,T478,Q498,N501,Y505

C1116	G339,S371,S373,S375,L452,T478,Q498,Y505
C1117	G339,S371,S373,S375,N440
C1118	G339,S371,S373,S375,N440,E484
C1119	G339,S371,S373,S375,N440,E484,N501
C1120	G339,S371,S373,S375,N440,E484,N501,Y505
C1121	G339,S371,S373,S375,N440,E484,Q493,N501,Y505
C1122	G339,S371,S373,S375,N440,E484,Q493,Q498,N501,Y505
C1123	G339,S371,S373,S375,N440,E484,Q493,Q498,Y505
C1124	G339,S371,S373,S375,N440,E484,Q498
C1125	G339,S371,S373,S375,N440,E484,Q498,N501,Y505
C1126	G339,S371,S373,S375,N440,E484,Q498,Y505
C1127	G339,S371,S373,S375,N440,E484,Y505
C1128	G339,S371,S373,S375,N440,G446
C1129	G339,S371,S373,S375,N440,G446,E484,Q493
C1130	G339,S371,S373,S375,N440,G446,E484,Q493,G496,N501,Y505
C1131	G339,S371,S373,S375,N440,G446,E484,Q493,G496,Q498,Y505
C1132	G339,S371,S373,S375,N440,G446,E484,Q493,G496,Y505
C1133	G339,S371,S373,S375,N440,G446,G496,Q498
C1134	G339,S371,S373,S375,N440,G446,G496,Q498,Y505
C1135	G339,S371,S373,S375,N440,G446,G496,Y505
C1136	G339,S371,S373,S375,N440,G446,N501,Y505
C1137	G339,S371,S373,S375,N440,G446,Q493
C1138	G339,S371,S373,S375,N440,G446,Q493,G496
C1139	G339,S371,S373,S375,N440,G446,Q493,G496,Q498
C1140	G339,S371,S373,S375,N440,G446,Q493,G496,Q498,N501
C1141	G339,S371,S373,S375,N440,G446,Q493,G496,Q498,N501,Y505
C1142	G339,S371,S373,S375,N440,G446,Q493,G496,Q498,Y505
C1143	G339,S371,S373,S375,N440,G446,S477
C1144	G339,S371,S373,S375,N440,G446,S477,E484
C1145	G339,S371,S373,S375,N440,G446,S477,E484,Q493,G496
C1146	G339,S371,S373,S375,N440,G446,S477,E484,Q493,G496,Q498
C1147	G339,S371,S373,S375,N440,G446,S477,E484,Q493,G496,Q498,N501
C1148	G339,S371,S373,S375,N440,G446,S477,E484,Q493,G496,Q498,N501,Y505
C1149	G339,S371,S373,S375,N440,G446,S477,E484,Q493,G496,Q498,Y505
C1150	G339,S371,S373,S375,N440,G446,S477,Q493,G496,Q498,N501,Y505
C1151	G339,S371,S373,S375,N440,G446,S477,Q493,G496,Q498,Y505
C1152	G339,S371,S373,S375,N440,G446,S477,T478
C1153	G339,S371,S373,S375,N440,G446,S477,T478,E484
C1154	G339,S371,S373,S375,N440,G446,S477,T478,E484,G496,Q498,Y505
C1155	G339,S371,S373,S375,N440,G446,S477,T478,E484,Q493
C1156	G339,S371,S373,S375,N440,G446,S477,T478,E484,Q493,G496
C1157	G339,S371,S373,S375,N440,G446,S477,T478,E484,Q493,G496,Q498
C1158	G339,S371,S373,S375,N440,G446,S477,T478,E484,Q493,G496,Y505

C1159	G339,S371,S373,S375,N440,G446,S477,T478,E484,Q493,Q498,Y505
C1160	G339,S371,S373,S375,N440,G446,S477,T478,E484,Q493,Y505
C1161	G339,S371,S373,S375,N440,G446,S477,T478,E484,Q498,Y505
C1162	G339,S371,S373,S375,N440,G446,S477,T478,E484,Y505
C1163	G339,S371,S373,S375,N440,G446,S477,T478,G496,Q498,Y505
C1164	G339,S371,S373,S375,N440,G446,S477,T478,Q493,G496,Q498,Y505
C1165	G339,S371,S373,S375,N440,G446,S477,T478,Y505
C1166	G339,S371,S373,S375,N440,G446,T478
C1167	G339,S371,S373,S375,N440,G446,T478,E484,Q493,G496,Q498,Y505
C1168	G339,S371,S373,S375,N440,G446,T478,G496
C1169	G339,S371,S373,S375,N440,G446,T478,G496,Q498
C1170	G339,S371,S373,S375,N440,G446,T478,G496,Q498,Y505
C1171	G339,S371,S373,S375,N440,G446,Y505
C1172	G339,S371,S373,S375,N440,G496,Q498,Y505
C1173	G339,S371,S373,S375,N440,N501
C1174	G339,S371,S373,S375,N440,N501,Y505
C1175	G339,S371,S373,S375,N440,Q493
C1176	G339,S371,S373,S375,N440,Q493,G496,Q498,N501,Y505
C1177	G339,S371,S373,S375,N440,Q493,G496,Q498,Y505
C1178	G339,S371,S373,S375,N440,Q493,Q498
C1179	G339,S371,S373,S375,N440,Q493,Q498,N501
C1180	G339,S371,S373,S375,N440,Q493,Q498,N501,Y505
C1181	G339,S371,S373,S375,N440,Q493,Q498,Y505
C1182	G339,S371,S373,S375,N440,Q498
C1183	G339,S371,S373,S375,N440,Q498,N501
C1184	G339,S371,S373,S375,N440,Q498,N501,Y505
C1185	G339,S371,S373,S375,N440,Q498,Y505
C1186	G339,S371,S373,S375,N440,S477
C1187	G339,S371,S373,S375,N440,S477,E484
C1188	G339,S371,S373,S375,N440,S477,E484,Q493,G496,Q498
C1189	G339,S371,S373,S375,N440,S477,E484,Q493,G496,Q498,N501,Y505
C1190	G339,S371,S373,S375,N440,S477,E484,Q493,Q498
C1191	G339,S371,S373,S375,N440,S477,E484,Q493,Q498,N501,Y505
C1192	G339,S371,S373,S375,N440,S477,E484,Q493,Q498,Y505
C1193	G339,S371,S373,S375,N440,S477,E484,Q498,N501,Y505
C1194	G339,S371,S373,S375,N440,S477,E484,Q498,Y505
C1195	G339,S371,S373,S375,N440,S477,T478
C1196	G339,S371,S373,S375,N440,S477,T478,E484
C1197	G339,S371,S373,S375,N440,S477,T478,E484,G496,Q498,Y505
C1198	G339,S371,S373,S375,N440,S477,T478,E484,N501
C1199	G339,S371,S373,S375,N440,S477,T478,E484,Q493
C1200	G339,S371,S373,S375,N440,S477,T478,E484,Q493,G496,Q498
C1201	G339,S371,S373,S375,N440,S477,T478,E484,Q493,G496,Q498,Y505

C1202	G339,S371,S373,S375,N440,S477,T478,E484,Q493,Q498
C1203	G339,S371,S373,S375,N440,S477,T478,E484,Q493,Q498,N501
C1204	G339,S371,S373,S375,N440,S477,T478,E484,Q493,Q498,N501,Y505
C1205	G339,S371,S373,S375,N440,S477,T478,E484,Q493,Q498,Y505
C1206	G339,S371,S373,S375,N440,S477,T478,E484,Q493,Y505
C1207	G339,S371,S373,S375,N440,S477,T478,E484,Q498
C1208	G339,S371,S373,S375,N440,S477,T478,E484,Q498,N501
C1209	G339,S371,S373,S375,N440,S477,T478,E484,Q498,N501,Y505
C1210	G339,S371,S373,S375,N440,S477,T478,E484,Q498,Y505
C1211	G339,S371,S373,S375,N440,S477,T478,N501
C1212	G339,S371,S373,S375,N440,S477,T478,Q493,Q498,N501,Y505
C1213	G339,S371,S373,S375,N440,S477,T478,Q493,Q498,Y505
C1214	G339,S371,S373,S375,N440,S477,T478,Q498
C1215	G339,S371,S373,S375,N440,S477,T478,Q498,N501
C1216	G339,S371,S373,S375,N440,S477,T478,Q498,N501,Y505
C1217	G339,S371,S373,S375,N440,S477,T478,Q498,Y505
C1218	G339,S371,S373,S375,N440,T478
C1219	G339,S371,S373,S375,N440,T478,E484
C1220	G339,S371,S373,S375,N440,T478,E484,Q498
C1221	G339,S371,S373,S375,N440,T478,E484,Q498,N501
C1222	G339,S371,S373,S375,N440,T478,E484,Q498,N501,Y505
C1223	G339,S371,S373,S375,N440,T478,E484,Q498,Y505
C1224	G339,S371,S373,S375,N440,T478,G496,Q498
C1225	G339,S371,S373,S375,N440,T478,G496,Q498,Y505
C1226	G339,S371,S373,S375,N440,T478,Q498
C1227	G339,S371,S373,S375,N440,T478,Q498,N501,Y505
C1228	G339,S371,S373,S375,N440,T478,Q498,Y505
C1229	G339,S371,S373,S375,N440,Y505
C1230	G339,S371,S373,S375,N501
C1231	G339,S371,S373,S375,N501,Y505
C1232	G339,S371,S373,S375,Q493
C1233	G339,S371,S373,S375,Q493,G496
C1234	G339,S371,S373,S375,Q493,G496,N501
C1235	G339,S371,S373,S375,Q493,G496,N501,Y505
C1236	G339,S371,S373,S375,Q493,G496,Q498
C1237	G339,S371,S373,S375,Q493,G496,Q498,N501
C1238	G339,S371,S373,S375,Q493,G496,Q498,N501,Y505
C1239	G339,S371,S373,S375,Q493,G496,Q498,Y505
C1240	G339,S371,S373,S375,Q493,G496,Y505
C1241	G339,S371,S373,S375,Q493,N501
C1242	G339,S371,S373,S375,Q493,N501,Y505
C1243	G339,S371,S373,S375,Q493,Q498
C1244	G339,S371,S373,S375,Q493,Q498,N501

C1245	G339,S371,S373,S375,Q493,Q498,N501,Y505
C1246	G339,S371,S373,S375,Q493,Q498,Y505
C1247	G339,S371,S373,S375,Q493,Y505
C1248	G339,S371,S373,S375,Q498
C1249	G339,S371,S373,S375,Q498,N501
C1250	G339,S371,S373,S375,Q498,N501,Y505
C1251	G339,S371,S373,S375,Q498,Y505
C1252	G339,S371,S373,S375,R408,E484,N501
C1253	G339,S371,S373,S375,R408,K417,N440,E484,N501
C1254	G339,S371,S373,S375,R408,K417,N440,L452,E484,N501
C1255	G339,S371,S373,S375,R408,K417,N440,L452,S477,T478,E484,Q498,Y505
C1256	G339,S371,S373,S375,R408,K417,N440,L452,T478,E484,Q498,N501,Y505
C1257	G339,S371,S373,S375,R408,K417,N440,S477,T478,E484,N501
C1258	G339,S371,S373,S375,R408,K417,N440,S477,T478,E484,Q493,Q498,N501,Y505
C1259	G339,S371,S373,S375,R408,K417,N440,S477,T478,E484,Q498,N501,Y505
C1260	G339,S371,S373,S375,R408,K417,N440,S477,T478,E484,Q498,Y505
C1261	G339,S371,S373,S375,R408,K417,N440,S477,T478,Q498,N501,Y505
C1262	G339,S371,S373,S375,R408,K417,N440,T478,E484,Q498,N501,Y505
C1263	G339,S371,S373,S375,R408,K417,S477,T478,E484,Q498,Y505
C1264	G339,S371,S373,S375,R408,S477,T478,E484,N501
C1265	G339,S371,S373,S375,R408,S477,T478,E484,Q493,Q498,N501,Y505
C1266	G339,S371,S373,S375,R408,S477,T478,E484,Q498,N501,Y505
C1267	G339,S371,S373,S375,R408,S477,T478,E484,Q498,Y505
C1268	G339,S371,S373,S375,R408,S477,T478,Q498,N501,Y505
C1269	G339,S371,S373,S375,R408,T478,E484,Q498,N501,Y505
C1270	G339,S371,S373,S375,S477
C1271	G339,S371,S373,S375,S477,E484
C1272	G339,S371,S373,S375,S477,E484,G496,N501
C1273	G339,S371,S373,S375,S477,E484,G496,Q498,N501,Y505
C1274	G339,S371,S373,S375,S477,E484,G496,Q498,Y505
C1275	G339,S371,S373,S375,S477,E484,N501
C1276	G339,S371,S373,S375,S477,E484,Q493
C1277	G339,S371,S373,S375,S477,E484,Q493,G496
C1278	G339,S371,S373,S375,S477,E484,Q493,G496,N501,Y505
C1279	G339,S371,S373,S375,S477,E484,Q493,G496,Q498
C1280	G339,S371,S373,S375,S477,E484,Q493,G496,Q498,N501
C1281	G339,S371,S373,S375,S477,E484,Q493,G496,Q498,N501,Y505
C1282	G339,S371,S373,S375,S477,E484,Q493,G496,Q498,Y505
C1283	G339,S371,S373,S375,S477,E484,Q493,G496,Y505
C1284	G339,S371,S373,S375,S477,E484,Q493,Q498
C1285	G339,S371,S373,S375,S477,E484,Q493,Q498,N501
C1286	G339,S371,S373,S375,S477,E484,Q493,Q498,N501,Y505
C1287	G339,S371,S373,S375,S477,E484,Q493,Q498,Y505

C1288	G339,S371,S373,S375,S477,E484,Q493,Y505
C1289	G339,S371,S373,S375,S477,E484,Q498
C1290	G339,S371,S373,S375,S477,E484,Q498,N501
C1291	G339,S371,S373,S375,S477,E484,Q498,N501,Y505
C1292	G339,S371,S373,S375,S477,E484,Q498,Y505
C1293	G339,S371,S373,S375,S477,E484,Y505
C1294	G339,S371,S373,S375,S477,G496,Q498,N501,Y505
C1295	G339,S371,S373,S375,S477,G496,Q498,Y505
C1296	G339,S371,S373,S375,S477,Q493,G496,Q498,N501,Y505
C1297	G339,S371,S373,S375,S477,Q493,G496,Q498,Y505
C1298	G339,S371,S373,S375,S477,Q493,Q498,N501,Y505
C1299	G339,S371,S373,S375,S477,Q493,Q498,Y505
C1300	G339,S371,S373,S375,S477,Q498,N501,Y505
C1301	G339,S371,S373,S375,S477,Q498,Y505
C1302	G339,S371,S373,S375,S477,T478
C1303	G339,S371,S373,S375,S477,T478,E484
C1304	G339,S371,S373,S375,S477,T478,E484,F486,Q498,N501,Y505
C1305	G339,S371,S373,S375,S477,T478,E484,F486,Q498,Y505
C1306	G339,S371,S373,S375,S477,T478,E484,G496
C1307	G339,S371,S373,S375,S477,T478,E484,G496,Q498,Y505
C1308	G339,S371,S373,S375,S477,T478,E484,N501
C1309	G339,S371,S373,S375,S477,T478,E484,N501,Y505
C1310	G339,S371,S373,S375,S477,T478,E484,Q493
C1311	G339,S371,S373,S375,S477,T478,E484,Q493,G496
C1312	G339,S371,S373,S375,S477,T478,E484,Q493,G496,Q498
C1313	G339,S371,S373,S375,S477,T478,E484,Q493,G496,Q498,Y505
C1314	G339,S371,S373,S375,S477,T478,E484,Q493,G496,Y505
C1315	G339,S371,S373,S375,S477,T478,E484,Q493,N501,Y505
C1316	G339,S371,S373,S375,S477,T478,E484,Q493,Q498
C1317	G339,S371,S373,S375,S477,T478,E484,Q493,Q498,N501
C1318	G339,S371,S373,S375,S477,T478,E484,Q493,Q498,N501,Y505
C1319	G339,S371,S373,S375,S477,T478,E484,Q493,Q498,Y505
C1320	G339,S371,S373,S375,S477,T478,E484,Q498
C1321	G339,S371,S373,S375,S477,T478,E484,Q498,N501
C1322	G339,S371,S373,S375,S477,T478,E484,Q498,N501,Y505
C1323	G339,S371,S373,S375,S477,T478,E484,Q498,Y505
C1324	G339,S371,S373,S375,S477,T478,E484,Y505
C1325	G339,S371,S373,S375,S477,T478,G496,N501
C1326	G339,S371,S373,S375,S477,T478,G496,Q498,N501,Y505
C1327	G339,S371,S373,S375,S477,T478,G496,Q498,Y505
C1328	G339,S371,S373,S375,S477,T478,N501
C1329	G339,S371,S373,S375,S477,T478,N501,Y505
C1330	G339,S371,S373,S375,S477,T478,Q493

C1331	G339,S371,S373,S375,S477,T478,Q493,G496
C1332	G339,S371,S373,S375,S477,T478,Q493,G496,N501,Y505
C1333	G339,S371,S373,S375,S477,T478,Q493,G496,Q498
C1334	G339,S371,S373,S375,S477,T478,Q493,G496,Q498,N501
C1335	G339,S371,S373,S375,S477,T478,Q493,G496,Q498,Y505
C1336	G339,S371,S373,S375,S477,T478,Q493,G496,Y505
C1337	G339,S371,S373,S375,S477,T478,Q493,N501,Y505
C1338	G339,S371,S373,S375,S477,T478,Q493,Q498
C1339	G339,S371,S373,S375,S477,T478,Q493,Q498,N501
C1340	G339,S371,S373,S375,S477,T478,Q493,Q498,N501,Y505
C1341	G339,S371,S373,S375,S477,T478,Q493,Q498,Y505
C1342	G339,S371,S373,S375,S477,T478,Q493,Y505
C1343	G339,S371,S373,S375,S477,T478,Q498
C1344	G339,S371,S373,S375,S477,T478,Q498,N501
C1345	G339,S371,S373,S375,S477,T478,Q498,N501,Y505
C1346	G339,S371,S373,S375,S477,T478,Q498,Y505
C1347	G339,S371,S373,S375,S477,T478,Y505
C1348	G339,S371,S373,S375,S477,Y505
C1349	G339,S371,S373,S375,T376
C1350	G339,S371,S373,S375,T376,D405
C1351	G339,S371,S373,S375,T376,D405,E484
C1352	G339,S371,S373,S375,T376,D405,E484,F486
C1353	G339,S371,S373,S375,T376,D405,E484,F486,Q498
C1354	G339,S371,S373,S375,T376,D405,E484,F486,Q498,N501
C1355	G339,S371,S373,S375,T376,D405,E484,F486,Q498,N501,Y505
C1356	G339,S371,S373,S375,T376,D405,E484,F486,Q498,Y505
C1357	G339,S371,S373,S375,T376,D405,E484,N501
C1358	G339,S371,S373,S375,T376,D405,E484,Q493
C1359	G339,S371,S373,S375,T376,D405,E484,Q493,Q498
C1360	G339,S371,S373,S375,T376,D405,E484,Q493,Q498,N501
C1361	G339,S371,S373,S375,T376,D405,E484,Q493,Q498,N501,Y505
C1362	G339,S371,S373,S375,T376,D405,E484,Q498
C1363	G339,S371,S373,S375,T376,D405,E484,Q498,N501
C1364	G339,S371,S373,S375,T376,D405,E484,Q498,N501,Y505
C1365	G339,S371,S373,S375,T376,D405,E484,Q498,Y505
C1366	G339,S371,S373,S375,T376,D405,F486,N501,Y505
C1367	G339,S371,S373,S375,T376,D405,F486,Q498
C1368	G339,S371,S373,S375,T376,D405,F486,Q498,N501
C1369	G339,S371,S373,S375,T376,D405,F486,Q498,N501,Y505
C1370	G339,S371,S373,S375,T376,D405,F486,Q498,Y505
C1371	G339,S371,S373,S375,T376,D405,F486,Y505
C1372	G339,S371,S373,S375,T376,D405,K417,E484,F486,Q498,N501,Y505
C1373	G339,S371,S373,S375,T376,D405,K417,E484,F486,Q498,Y505

C1374	G339,S371,S373,S375,T376,D405,K417,E484,N501
C1375	G339,S371,S373,S375,T376,D405,K417,E484,Q493,Q498,N501,Y505
C1376	G339,S371,S373,S375,T376,D405,K417,E484,Q498,N501,Y505
C1377	G339,S371,S373,S375,T376,D405,K417,E484,Q498,Y505
C1378	G339,S371,S373,S375,T376,D405,K417,F486,Q498,N501,Y505
C1379	G339,S371,S373,S375,T376,D405,K417,F486,Q498,Y505
C1380	G339,S371,S373,S375,T376,D405,K417,L452,E484,N501
C1381	G339,S371,S373,S375,T376,D405,K417,L452,S477,T478,E484,F486,Q498,Y505
C1382	G339,S371,S373,S375,T376,D405,K417,L452,S477,T478,E484,Q498,Y505
C1383	G339,S371,S373,S375,T376,D405,K417,L452,T478,E484,F486,Q498,N501,Y505
C1384	G339,S371,S373,S375,T376,D405,K417,L452,T478,E484,Q498,N501,Y505
C1385	G339,S371,S373,S375,T376,D405,K417,N440,E484,F486,Q498,N501,Y505
C1386	G339,S371,S373,S375,T376,D405,K417,N440,E484,F486,Q498,Y505
C1387	G339,S371,S373,S375,T376,D405,K417,N440,E484,N501
C1388	G339,S371,S373,S375,T376,D405,K417,N440,E484,Q498,N501,Y505
C1389	G339,S371,S373,S375,T376,D405,K417,N440,L452,E484,F486,Q498,N501,Y505
C1390	G339,S371,S373,S375,T376,D405,K417,N440,L452,E484,F486,Q498,Y505
C1391	G339,S371,S373,S375,T376,D405,K417,N440,L452,E484,N501
C1392	G339,S371,S373,S375,T376,D405,K417,N440,L452,N501
C1393	G339,S371,S373,S375,T376,D405,K417,N440,L452,S477,T478,E484,F486,Q498,Y505
C1394	G339,S371,S373,S375,T376,D405,K417,N440,L452,S477,T478,E484,Q498,Y505
C1395	G339,S371,S373,S375,T376,D405,K417,N440,L452,S477,T478,Q498,Y505
C1396	G339,S371,S373,S375,T376,D405,K417,N440,L452,T478,E484,F486,Q498,N501,Y505
C1397	G339,S371,S373,S375,T376,D405,K417,N440,L452,T478,E484,F486,Q498,Y505
C1398	G339,S371,S373,S375,T376,D405,K417,N440,L452,T478,E484,Q498,N501,Y505
C1399	G339,S371,S373,S375,T376,D405,K417,N440,L452,T478,Q498,N501,Y505
C1400	G339,S371,S373,S375,T376,D405,K417,N440,Q498,N501,Y505
C1401	G339,S371,S373,S375,T376,D405,K417,N440,S477,T478,E484,F486,Q498,N501,Y505
C1402	G339,S371,S373,S375,T376,D405,K417,N440,S477,T478,E484,F486,Q498,Y505
C1403	G339,S371,S373,S375,T376,D405,K417,N440,S477,T478,E484,N501
C1404	G339,S371,S373,S375,T376,D405,K417,N440,S477,T478,E484,Q493,Q498,N501,Y505
C1405	G339,S371,S373,S375,T376,D405,K417,N440,S477,T478,E484,Q498,N501,Y505
C1406	G339,S371,S373,S375,T376,D405,K417,N440,S477,T478,E484,Q498,Y505
C1407	G339,S371,S373,S375,T376,D405,K417,N440,S477,T478,N501
C1408	G339,S371,S373,S375,T376,D405,K417,N440,S477,T478,Q493,Q498,N501,Y505
C1409	G339,S371,S373,S375,T376,D405,K417,N440,S477,T478,Q498,N501,Y505
C1410	G339,S371,S373,S375,T376,D405,K417,N440,T478,E484,F486,Q498,N501,Y505
C1411	G339,S371,S373,S375,T376,D405,K417,N440,T478,E484,F486,Q498,Y505
C1412	G339,S371,S373,S375,T376,D405,K417,N440,T478,E484,N501
C1413	G339,S371,S373,S375,T376,D405,K417,N440,T478,E484,Q498,N501,Y505
C1414	G339,S371,S373,S375,T376,D405,K417,N440,T478,Q498,N501,Y505
C1415	G339,S371,S373,S375,T376,D405,K417,N501
C1416	G339,S371,S373,S375,T376,D405,K417,Q498,N501,Y505

C1417	G339,S371,S373,S375,T376,D405,K417,Q498,Y505
C1418	G339,S371,S373,S375,T376,D405,K417,S477,T478,E484,F486,Q498,N501,Y505
C1419	G339,S371,S373,S375,T376,D405,K417,S477,T478,E484,F486,Q498,Y505
C1420	G339,S371,S373,S375,T376,D405,K417,S477,T478,E484,N501
C1421	G339,S371,S373,S375,T376,D405,K417,S477,T478,E484,Q493,Q498,N501,Y505
C1422	G339,S371,S373,S375,T376,D405,K417,S477,T478,E484,Q498,N501,Y505
C1423	G339,S371,S373,S375,T376,D405,K417,S477,T478,E484,Q498,Y505
C1424	G339,S371,S373,S375,T376,D405,K417,S477,T478,Q498,N501,Y505
C1425	G339,S371,S373,S375,T376,D405,K417,S477,T478,Q498,Y505
C1426	G339,S371,S373,S375,T376,D405,K417,T478,E484,F486,Q498,N501,Y505
C1427	G339,S371,S373,S375,T376,D405,K417,T478,E484,F486,Q498,Y505
C1428	G339,S371,S373,S375,T376,D405,K417,T478,E484,N501
C1429	G339,S371,S373,S375,T376,D405,K417,T478,E484,Q498,N501,Y505
C1430	G339,S371,S373,S375,T376,D405,K417,T478,E484,Q498,Y505
C1431	G339,S371,S373,S375,T376,D405,K417,T478,Q498,N501,Y505
C1432	G339,S371,S373,S375,T376,D405,L452,E484,F486,Q498,N501,Y505
C1433	G339,S371,S373,S375,T376,D405,L452,E484,F486,Q498,Y505
C1434	G339,S371,S373,S375,T376,D405,L452,E484,N501
C1435	G339,S371,S373,S375,T376,D405,L452,S477,T478,E484,F486,Q498,Y505
C1436	G339,S371,S373,S375,T376,D405,L452,S477,T478,E484,Q498,Y505
C1437	G339,S371,S373,S375,T376,D405,L452,T478,E484,F486,Q498,N501,Y505
C1438	G339,S371,S373,S375,T376,D405,L452,T478,E484,Q498,N501,Y505
C1439	G339,S371,S373,S375,T376,D405,L452,T478,E484,Q498,Y505
C1440	G339,S371,S373,S375,T376,D405,N440,E484,F486,Q498,N501,Y505
C1441	G339,S371,S373,S375,T376,D405,N440,E484,F486,Q498,Y505
C1442	G339,S371,S373,S375,T376,D405,N440,E484,N501
C1443	G339,S371,S373,S375,T376,D405,N440,E484,Q493,Q498,N501,Y505
C1444	G339,S371,S373,S375,T376,D405,N440,E484,Q498,N501,Y505
C1445	G339,S371,S373,S375,T376,D405,N440,E484,Q498,Y505
C1446	G339,S371,S373,S375,T376,D405,N440,L452,E484
C1447	G339,S371,S373,S375,T376,D405,N440,L452,E484,F486,Q498,N501,Y505
C1448	G339,S371,S373,S375,T376,D405,N440,L452,E484,F486,Q498,Y505
C1449	G339,S371,S373,S375,T376,D405,N440,L452,E484,N501
C1450	G339,S371,S373,S375,T376,D405,N440,L452,E484,Q498,N501,Y505
C1451	G339,S371,S373,S375,T376,D405,N440,L452,E484,Q498,Y505
C1452	G339,S371,S373,S375,T376,D405,N440,L452,S477,T478,E484,F486
C1453	G339,S371,S373,S375,T376,D405,N440,L452,S477,T478,E484,F486,Q498,Y505
C1454	G339,S371,S373,S375,T376,D405,N440,L452,S477,T478,E484,Q498,Y505
C1455	G339,S371,S373,S375,T376,D405,N440,L452,T478,E484,F486
C1456	G339,S371,S373,S375,T376,D405,N440,L452,T478,E484,F486,Q498,N501,Y505
C1457	G339,S371,S373,S375,T376,D405,N440,L452,T478,E484,F486,Q498,Y505
C1458	G339,S371,S373,S375,T376,D405,N440,L452,T478,E484,Q498,N501,Y505
C1459	G339,S371,S373,S375,T376,D405,N440,Q498,N501,Y505

C1460	G339,S371,S373,S375,T376,D405,N440,S477,T478
C1461	G339,S371,S373,S375,T376,D405,N440,S477,T478,E484
C1462	G339,S371,S373,S375,T376,D405,N440,S477,T478,E484,F486
C1463	G339,S371,S373,S375,T376,D405,N440,S477,T478,E484,F486,Q498,N501,Y505
C1464	G339,S371,S373,S375,T376,D405,N440,S477,T478,E484,F486,Q498,Y505
C1465	G339,S371,S373,S375,T376,D405,N440,S477,T478,E484,N501
C1466	G339,S371,S373,S375,T376,D405,N440,S477,T478,E484,Q493,Q498,N501,Y505
C1467	G339,S371,S373,S375,T376,D405,N440,S477,T478,E484,Q498,N501,Y505
C1468	G339,S371,S373,S375,T376,D405,N440,S477,T478,E484,Q498,Y505
C1469	G339,S371,S373,S375,T376,D405,N440,S477,T478,Q498,N501,Y505
C1470	G339,S371,S373,S375,T376,D405,N440,T478,E484,F486,Q498,N501,Y505
C1471	G339,S371,S373,S375,T376,D405,N440,T478,E484,F486,Q498,Y505
C1472	G339,S371,S373,S375,T376,D405,N440,T478,E484,N501
C1473	G339,S371,S373,S375,T376,D405,N440,T478,E484,Q498,N501,Y505
C1474	G339,S371,S373,S375,T376,D405,N440,T478,Q498,N501,Y505
C1475	G339,S371,S373,S375,T376,D405,N501
C1476	G339,S371,S373,S375,T376,D405,N501,Y505
C1477	G339,S371,S373,S375,T376,D405,Q493,N501
C1478	G339,S371,S373,S375,T376,D405,Q493,Q498,N501
C1479	G339,S371,S373,S375,T376,D405,Q493,Q498,N501,Y505
C1480	G339,S371,S373,S375,T376,D405,Q498
C1481	G339,S371,S373,S375,T376,D405,Q498,N501
C1482	G339,S371,S373,S375,T376,D405,Q498,N501,Y505
C1483	G339,S371,S373,S375,T376,D405,Q498,Y505
C1484	G339,S371,S373,S375,T376,D405,R408,E484,N501
C1485	G339,S371,S373,S375,T376,D405,R408,K417
C1486	G339,S371,S373,S375,T376,D405,R408,K417,E484
C1487	G339,S371,S373,S375,T376,D405,R408,K417,E484,N501
C1488	G339,S371,S373,S375,T376,D405,R408,K417,E484,Q498
C1489	G339,S371,S373,S375,T376,D405,R408,K417,E484,Q498,Y505
C1490	G339,S371,S373,S375,T376,D405,R408,K417,L452
C1491	G339,S371,S373,S375,T376,D405,R408,K417,L452,E484
C1492	G339,S371,S373,S375,T376,D405,R408,K417,L452,E484,N501
C1493	G339,S371,S373,S375,T376,D405,R408,K417,L452,S477,T478,E484,F486,Q498,Y505
C1494	G339,S371,S373,S375,T376,D405,R408,K417,L452,S477,T478,E484,Q498,Y505
C1495	G339,S371,S373,S375,T376,D405,R408,K417,L452,T478
C1496	G339,S371,S373,S375,T376,D405,R408,K417,L452,T478,E484,F486,Q498,N501,Y505
C1497	G339,S371,S373,S375,T376,D405,R408,K417,L452,T478,E484,F486,Q498,Y505
C1498	G339,S371,S373,S375,T376,D405,R408,K417,L452,T478,E484,Q498,N501,Y505
C1499	G339,S371,S373,S375,T376,D405,R408,K417,N440
C1500	G339,S371,S373,S375,T376,D405,R408,K417,N440,E484
C1501	G339,S371,S373,S375,T376,D405,R408,K417,N440,E484,F486,Q498,N501
C1502	G339,S371,S373,S375,T376,D405,R408,K417,N440,E484,F486,Q498,N501,Y505

C1503	G339,S371,S373,S375,T376,D405,R408,K417,N440,E484,N501
C1504	G339,S371,S373,S375,T376,D405,R408,K417,N440,E484,Q493,Q498,N501,Y505
C1505	G339,S371,S373,S375,T376,D405,R408,K417,N440,E484,Q498,N501
C1506	G339,S371,S373,S375,T376,D405,R408,K417,N440,E484,Q498,N501,Y505
C1507	G339,S371,S373,S375,T376,D405,R408,K417,N440,E484,Q498,Y505
C1508	G339,S371,S373,S375,T376,D405,R408,K417,N440,L452
C1509	G339,S371,S373,S375,T376,D405,R408,K417,N440,L452,E484
C1510	G339,S371,S373,S375,T376,D405,R408,K417,N440,L452,E484,F486,Q498
C1511	G339,S371,S373,S375,T376,D405,R408,K417,N440,L452,E484,F486,Q498,N501
C1512	G339,S371,S373,S375,T376,D405,R408,K417,N440,L452,E484,F486,Q498,N501,Y505
C1513	G339,S371,S373,S375,T376,D405,R408,K417,N440,L452,E484,F486,Q498,Y505
C1514	G339,S371,S373,S375,T376,D405,R408,K417,N440,L452,E484,Q498,N501,Y505
C1515	G339,S371,S373,S375,T376,D405,R408,K417,N440,L452,N501
C1516	G339,S371,S373,S375,T376,D405,R408,K417,N440,L452,Q498,N501,Y505
C1517	G339,S371,S373,S375,T376,D405,R408,K417,N440,L452,Q498,Y505
C1518	G339,S371,S373,S375,T376,D405,R408,K417,N440,L452,S477,E484,F486,Q498,Y505
C1519	G339,S371,S373,S375,T376,D405,R408,K417,N440,L452,S477,E484,Q498,Y505
C1520	G339,S371,S373,S375,T376,D405,R408,K417,N440,L452,S477,T478,E484
C1521	G339,S371,S373,S375,T376,D405,R408,K417,N440,L452,S477,T478,E484,F486
C1522	G339,S371,S373,S375,T376,D405,R408,K417,N440,L452,S477,T478,E484,F486,Q498
C1523	G339,S371,S373,S375,T376,D405,R408,K417,N440,L452,S477,T478,E484,Q498
C1524	G339,S371,S373,S375,T376,D405,R408,K417,N440,L452,S477,T478,E484,Q498,Y505
C1525	G339,S371,S373,S375,T376,D405,R408,K417,N440,L452,S477,T478,E484,Y505
C1526	G339,S371,S373,S375,T376,D405,R408,K417,N440,L452,S477,T478,F486,Q498,Y505
C1527	G339,S371,S373,S375,T376,D405,R408,K417,N440,L452,S477,T478,Q498,Y505
C1528	G339,S371,S373,S375,T376,D405,R408,K417,N440,L452,T478,E484
C1529	G339,S371,S373,S375,T376,D405,R408,K417,N440,L452,T478,E484,F486
C1530	G339,S371,S373,S375,T376,D405,R408,K417,N440,L452,T478,E484,F486,Q498,N501
C1531	G339,S371,S373,S375,T376,D405,R408,K417,N440,L452,T478,E484,F486,Q498,Y505
C1532	G339,S371,S373,S375,T376,D405,R408,K417,N440,L452,T478,E484,N501,Y505
C1533	G339,S371,S373,S375,T376,D405,R408,K417,N440,L452,T478,E484,Q498
C1534	G339,S371,S373,S375,T376,D405,R408,K417,N440,L452,T478,E484,Q498,N501
C1535	G339,S371,S373,S375,T376,D405,R408,K417,N440,L452,T478,E484,Q498,N501,Y505
C1536	G339,S371,S373,S375,T376,D405,R408,K417,N440,L452,T478,F486,Q498,N501,Y505
C1537	G339,S371,S373,S375,T376,D405,R408,K417,N440,L452,T478,Q498,N501,Y505
C1538	G339,S371,S373,S375,T376,D405,R408,K417,N440,N501
C1539	G339,S371,S373,S375,T376,D405,R408,K417,N440,Q493,Q498,N501,Y505
C1540	G339,S371,S373,S375,T376,D405,R408,K417,N440,Q498,N501
C1541	G339,S371,S373,S375,T376,D405,R408,K417,N440,Q498,N501,Y505
C1542	G339,S371,S373,S375,T376,D405,R408,K417,N440,Q498,Y505
C1543	G339,S371,S373,S375,T376,D405,R408,K417,N440,S477,E484,F486,Q498,N501,Y505
C1544	G339,S371,S373,S375,T376,D405,R408,K417,N440,S477,E484,N501
C1545	G339,S371,S373,S375,T376,D405,R408,K417,N440,S477,E484,Q493,Q498,N501,Y505

C1546	G339,S371,S373,S375,T376,D405,R408,K417,N440,S477,E484,Q498,N501,Y505
C1547	G339,S371,S373,S375,T376,D405,R408,K417,N440,S477,Q498,N501,Y505
C1548	G339,S371,S373,S375,T376,D405,R408,K417,N440,S477,T478
C1549	G339,S371,S373,S375,T376,D405,R408,K417,N440,S477,T478,E484
C1550	G339,S371,S373,S375,T376,D405,R408,K417,N440,S477,T478,E484,F486
C1551	G339,S371,S373,S375,T376,D405,R408,K417,N440,S477,T478,E484,F486,Q498,N501
C1552	G339,S371,S373,S375,T376,D405,R408,K417,N440,S477,T478,E484,F486,Q498,Y505
C1553	G339,S371,S373,S375,T376,D405,R408,K417,N440,S477,T478,E484,N501,Y505
C1554	G339,S371,S373,S375,T376,D405,R408,K417,N440,S477,T478,E484,Q493
C1555	G339,S371,S373,S375,T376,D405,R408,K417,N440,S477,T478,E484,Q493,N501,Y505
C1556	G339,S371,S373,S375,T376,D405,R408,K417,N440,S477,T478,E484,Q493,Q498
C1557	G339,S371,S373,S375,T376,D405,R408,K417,N440,S477,T478,E484,Q493,Q498,N501
C1558	G339,S371,S373,S375,T376,D405,R408,K417,N440,S477,T478,E484,Q493,Q498,Y505
C1559	G339,S371,S373,S375,T376,D405,R408,K417,N440,S477,T478,E484,Q498
C1560	G339,S371,S373,S375,T376,D405,R408,K417,N440,S477,T478,E484,Q498,N501
C1561	G339,S371,S373,S375,T376,D405,R408,K417,N440,S477,T478,E484,Q498,N501,Y505
C1562	G339,S371,S373,S375,T376,D405,R408,K417,N440,S477,T478,E484,Q498,Y505
C1563	G339,S371,S373,S375,T376,D405,R408,K417,N440,S477,T478,E484,Y505
C1564	G339,S371,S373,S375,T376,D405,R408,K417,N440,S477,T478,F486,Q498,N501,Y505
C1565	G339,S371,S373,S375,T376,D405,R408,K417,N440,S477,T478,N501
C1566	G339,S371,S373,S375,T376,D405,R408,K417,N440,S477,T478,N501,Y505
C1567	G339,S371,S373,S375,T376,D405,R408,K417,N440,S477,T478,Q493,Q498,N501,Y505
C1568	G339,S371,S373,S375,T376,D405,R408,K417,N440,S477,T478,Q498
C1569	G339,S371,S373,S375,T376,D405,R408,K417,N440,S477,T478,Q498,N501
C1570	G339,S371,S373,S375,T376,D405,R408,K417,N440,S477,T478,Q498,Y505
C1571	G339,S371,S373,S375,T376,D405,R408,K417,N440,T478
C1572	G339,S371,S373,S375,T376,D405,R408,K417,N440,T478,E484
C1573	G339,S371,S373,S375,T376,D405,R408,K417,N440,T478,E484,F486,Q498,N501,Y505
C1574	G339,S371,S373,S375,T376,D405,R408,K417,N440,T478,E484,N501
C1575	G339,S371,S373,S375,T376,D405,R408,K417,N440,T478,E484,N501,Y505
C1576	G339,S371,S373,S375,T376,D405,R408,K417,N440,T478,E484,Q498
C1577	G339,S371,S373,S375,T376,D405,R408,K417,N440,T478,E484,Q498,N501
C1578	G339,S371,S373,S375,T376,D405,R408,K417,N440,T478,E484,Q498,N501,Y505
C1579	G339,S371,S373,S375,T376,D405,R408,K417,N440,T478,E484,Q498,Y505
C1580	G339,S371,S373,S375,T376,D405,R408,K417,N440,T478,Q498,N501,Y505
C1581	G339,S371,S373,S375,T376,D405,R408,K417,N501
C1582	G339,S371,S373,S375,T376,D405,R408,K417,Q493,Q498,N501,Y505
C1583	G339,S371,S373,S375,T376,D405,R408,K417,Q498,N501,Y505
C1584	G339,S371,S373,S375,T376,D405,R408,K417,Q498,Y505
C1585	G339,S371,S373,S375,T376,D405,R408,K417,S477,E484,Q498,Y505
C1586	G339,S371,S373,S375,T376,D405,R408,K417,S477,T478
C1587	G339,S371,S373,S375,T376,D405,R408,K417,S477,T478,E484
C1588	G339,S371,S373,S375,T376,D405,R408,K417,S477,T478,E484,F486,Q498,N501,Y505

C1589	G339,S371,S373,S375,T376,D405,R408,K417,S477,T478,E484,F486,Q498,Y505
C1590	G339,S371,S373,S375,T376,D405,R408,K417,S477,T478,E484,N501
C1591	G339,S371,S373,S375,T376,D405,R408,K417,S477,T478,E484,Q493
C1592	G339,S371,S373,S375,T376,D405,R408,K417,S477,T478,E484,Q493,Q498,N501,Y505
C1593	G339,S371,S373,S375,T376,D405,R408,K417,S477,T478,E484,Q498
C1594	G339,S371,S373,S375,T376,D405,R408,K417,S477,T478,E484,Q498,N501,Y505
C1595	G339,S371,S373,S375,T376,D405,R408,K417,S477,T478,E484,Y505
C1596	G339,S371,S373,S375,T376,D405,R408,K417,S477,T478,Q498,N501,Y505
C1597	G339,S371,S373,S375,T376,D405,R408,K417,S477,T478,Q498,Y505
C1598	G339,S371,S373,S375,T376,D405,R408,K417,T478
C1599	G339,S371,S373,S375,T376,D405,R408,K417,T478,E484
C1600	G339,S371,S373,S375,T376,D405,R408,K417,T478,E484,F486,Q498,N501,Y505
C1601	G339,S371,S373,S375,T376,D405,R408,K417,T478,E484,Q498,N501,Y505
C1602	G339,S371,S373,S375,T376,D405,R408,K417,T478,E484,Q498,Y505
C1603	G339,S371,S373,S375,T376,D405,R408,L452,E484,N501
C1604	G339,S371,S373,S375,T376,D405,R408,L452,S477,T478,E484,F486,Q498,Y505
C1605	G339,S371,S373,S375,T376,D405,R408,L452,S477,T478,E484,Q498,Y505
C1606	G339,S371,S373,S375,T376,D405,R408,L452,T478,E484,F486,Q498,N501,Y505
C1607	G339,S371,S373,S375,T376,D405,R408,L452,T478,E484,Q498,N501,Y505
C1608	G339,S371,S373,S375,T376,D405,R408,N440,E484
C1609	G339,S371,S373,S375,T376,D405,R408,N440,E484,N501
C1610	G339,S371,S373,S375,T376,D405,R408,N440,L452,E484,N501
C1611	G339,S371,S373,S375,T376,D405,R408,N440,L452,S477,T478,E484,F486,Q498,Y505
C1612	G339,S371,S373,S375,T376,D405,R408,N440,L452,S477,T478,E484,Q498,Y505
C1613	G339,S371,S373,S375,T376,D405,R408,N440,L452,T478,E484,F486,Q498,N501,Y505
C1614	G339,S371,S373,S375,T376,D405,R408,N440,L452,T478,E484,Q498,N501,Y505
C1615	G339,S371,S373,S375,T376,D405,R408,N440,S477,T478,E484
C1616	G339,S371,S373,S375,T376,D405,R408,N440,S477,T478,E484,F486,Q498,N501,Y505
C1617	G339,S371,S373,S375,T376,D405,R408,N440,S477,T478,E484,F486,Q498,Y505
C1618	G339,S371,S373,S375,T376,D405,R408,N440,S477,T478,E484,N501
C1619	G339,S371,S373,S375,T376,D405,R408,N440,S477,T478,E484,Q493,Q498
C1620	G339,S371,S373,S375,T376,D405,R408,N440,S477,T478,E484,Q493,Q498,N501,Y505
C1621	G339,S371,S373,S375,T376,D405,R408,N440,S477,T478,E484,Q498
C1622	G339,S371,S373,S375,T376,D405,R408,N440,S477,T478,E484,Q498,N501,Y505
C1623	G339,S371,S373,S375,T376,D405,R408,N440,S477,T478,E484,Q498,Y505
C1624	G339,S371,S373,S375,T376,D405,R408,N440,S477,T478,Q498
C1625	G339,S371,S373,S375,T376,D405,R408,N440,S477,T478,Q498,N501,Y505
C1626	G339,S371,S373,S375,T376,D405,R408,N440,T478,E484,F486,Q498,N501,Y505
C1627	G339,S371,S373,S375,T376,D405,R408,N440,T478,E484,Q498
C1628	G339,S371,S373,S375,T376,D405,R408,N440,T478,E484,Q498,N501,Y505
C1629	G339,S371,S373,S375,T376,D405,R408,S477,T478,E484,F486,Q498,N501,Y505
C1630	G339,S371,S373,S375,T376,D405,R408,S477,T478,E484,F486,Q498,Y505
C1631	G339,S371,S373,S375,T376,D405,R408,S477,T478,E484,N501

C1632	G339,S371,S373,S375,T376,D405,R408,S477,T478,E484,Q493,Q498,N501,Y505
C1633	G339,S371,S373,S375,T376,D405,R408,S477,T478,E484,Q498
C1634	G339,S371,S373,S375,T376,D405,R408,S477,T478,E484,Q498,N501,Y505
C1635	G339,S371,S373,S375,T376,D405,R408,S477,T478,E484,Q498,Y505
C1636	G339,S371,S373,S375,T376,D405,R408,S477,T478,Q498,N501,Y505
C1637	G339,S371,S373,S375,T376,D405,R408,T478,E484,F486,Q498,N501,Y505
C1638	G339,S371,S373,S375,T376,D405,R408,T478,E484,Q498,N501,Y505
C1639	G339,S371,S373,S375,T376,D405,S477,E484,F486,Q498,N501,Y505
C1640	G339,S371,S373,S375,T376,D405,S477,E484,F486,Q498,Y505
C1641	G339,S371,S373,S375,T376,D405,S477,E484,N501
C1642	G339,S371,S373,S375,T376,D405,S477,E484,Q498,N501,Y505
C1643	G339,S371,S373,S375,T376,D405,S477,E484,Q498,Y505
C1644	G339,S371,S373,S375,T376,D405,S477,Q498,N501,Y505
C1645	G339,S371,S373,S375,T376,D405,S477,T478,E484
C1646	G339,S371,S373,S375,T376,D405,S477,T478,E484,F486,Q498
C1647	G339,S371,S373,S375,T376,D405,S477,T478,E484,F486,Q498,N501
C1648	G339,S371,S373,S375,T376,D405,S477,T478,E484,F486,Q498,N501,Y505
C1649	G339,S371,S373,S375,T376,D405,S477,T478,E484,F486,Q498,Y505
C1650	G339,S371,S373,S375,T376,D405,S477,T478,E484,N501
C1651	G339,S371,S373,S375,T376,D405,S477,T478,E484,Q493,Q498,N501
C1652	G339,S371,S373,S375,T376,D405,S477,T478,E484,Q493,Q498,N501,Y505
C1653	G339,S371,S373,S375,T376,D405,S477,T478,E484,Q498
C1654	G339,S371,S373,S375,T376,D405,S477,T478,E484,Q498,N501
C1655	G339,S371,S373,S375,T376,D405,S477,T478,E484,Q498,N501,Y505
C1656	G339,S371,S373,S375,T376,D405,S477,T478,E484,Q498,Y505
C1657	G339,S371,S373,S375,T376,D405,S477,T478,N501
C1658	G339,S371,S373,S375,T376,D405,S477,T478,Q493,Q498,N501,Y505
C1659	G339,S371,S373,S375,T376,D405,S477,T478,Q498,N501
C1660	G339,S371,S373,S375,T376,D405,S477,T478,Q498,N501,Y505
C1661	G339,S371,S373,S375,T376,D405,S477,T478,Q498,Y505
C1662	G339,S371,S373,S375,T376,D405,T478,E484
C1663	G339,S371,S373,S375,T376,D405,T478,E484,F486,Q498,N501
C1664	G339,S371,S373,S375,T376,D405,T478,E484,F486,Q498,N501,Y505
C1665	G339,S371,S373,S375,T376,D405,T478,E484,F486,Q498,Y505
C1666	G339,S371,S373,S375,T376,D405,T478,E484,N501
C1667	G339,S371,S373,S375,T376,D405,T478,E484,Q493,Q498,N501
C1668	G339,S371,S373,S375,T376,D405,T478,E484,Q493,Q498,N501,Y505
C1669	G339,S371,S373,S375,T376,D405,T478,E484,Q498
C1670	G339,S371,S373,S375,T376,D405,T478,E484,Q498,N501
C1671	G339,S371,S373,S375,T376,D405,T478,E484,Q498,N501,Y505
C1672	G339,S371,S373,S375,T376,D405,T478,E484,Q498,Y505
C1673	G339,S371,S373,S375,T376,D405,T478,Q498,N501
C1674	G339,S371,S373,S375,T376,D405,T478,Q498,N501,Y505

C1675	G339,S371,S373,S375,T376,D405,T478,Q498,Y505
C1676	G339,S371,S373,S375,T376,D405,Y505
C1677	G339,S371,S373,S375,T376,E484
C1678	G339,S371,S373,S375,T376,E484,F486
C1679	G339,S371,S373,S375,T376,E484,F486,Q498
C1680	G339,S371,S373,S375,T376,E484,F486,Q498,N501
C1681	G339,S371,S373,S375,T376,E484,F486,Q498,N501,Y505
C1682	G339,S371,S373,S375,T376,E484,F486,Q498,Y505
C1683	G339,S371,S373,S375,T376,E484,N501
C1684	G339,S371,S373,S375,T376,E484,Q493,Q498,N501
C1685	G339,S371,S373,S375,T376,E484,Q493,Q498,N501,Y505
C1686	G339,S371,S373,S375,T376,E484,Q498
C1687	G339,S371,S373,S375,T376,E484,Q498,N501
C1688	G339,S371,S373,S375,T376,E484,Q498,N501,Y505
C1689	G339,S371,S373,S375,T376,E484,Q498,Y505
C1690	G339,S371,S373,S375,T376,F486,N501,Y505
C1691	G339,S371,S373,S375,T376,F486,Q498
C1692	G339,S371,S373,S375,T376,F486,Q498,N501
C1693	G339,S371,S373,S375,T376,F486,Q498,N501,Y505
C1694	G339,S371,S373,S375,T376,F486,Q498,Y505
C1695	G339,S371,S373,S375,T376,F486,Y505
C1696	G339,S371,S373,S375,T376,K417,E484,N501
C1697	G339,S371,S373,S375,T376,K417,L452,E484,N501
C1698	G339,S371,S373,S375,T376,K417,L452,S477,T478,E484,F486,Q498,Y505
C1699	G339,S371,S373,S375,T376,K417,L452,S477,T478,E484,Q498,Y505
C1700	G339,S371,S373,S375,T376,K417,L452,T478,E484,F486,Q498,N501,Y505
C1701	G339,S371,S373,S375,T376,K417,L452,T478,E484,Q498,N501,Y505
C1702	G339,S371,S373,S375,T376,K417,N440,E484,N501
C1703	G339,S371,S373,S375,T376,K417,N440,L452,E484,N501
C1704	G339,S371,S373,S375,T376,K417,N440,L452,S477,T478,E484,F486,Q498,Y505
C1705	G339,S371,S373,S375,T376,K417,N440,L452,S477,T478,E484,Q498,Y505
C1706	G339,S371,S373,S375,T376,K417,N440,L452,T478,E484,F486,Q498,N501,Y505
C1707	G339,S371,S373,S375,T376,K417,N440,L452,T478,E484,Q498,N501,Y505
C1708	G339,S371,S373,S375,T376,K417,N440,S477,T478,E484,F486,Q498,N501,Y505
C1709	G339,S371,S373,S375,T376,K417,N440,S477,T478,E484,F486,Q498,Y505
C1710	G339,S371,S373,S375,T376,K417,N440,S477,T478,E484,N501
C1711	G339,S371,S373,S375,T376,K417,N440,S477,T478,E484,Q493,Q498,N501,Y505
C1712	G339,S371,S373,S375,T376,K417,N440,S477,T478,E484,Q498,N501,Y505
C1713	G339,S371,S373,S375,T376,K417,N440,S477,T478,E484,Q498,Y505
C1714	G339,S371,S373,S375,T376,K417,N440,S477,T478,Q498,N501,Y505
C1715	G339,S371,S373,S375,T376,K417,N440,T478,E484,F486,Q498,N501,Y505
C1716	G339,S371,S373,S375,T376,K417,N440,T478,E484,Q498,N501,Y505
C1717	G339,S371,S373,S375,T376,K417,S477,T478,E484,F486,Q498,N501,Y505

C1718	G339,S371,S373,S375,T376,K417,S477,T478,E484,F486,Q498,Y505
C1719	G339,S371,S373,S375,T376,K417,S477,T478,E484,N501
C1720	G339,S371,S373,S375,T376,K417,S477,T478,E484,Q493,Q498,N501,Y505
C1721	G339,S371,S373,S375,T376,K417,S477,T478,E484,Q498,N501,Y505
C1722	G339,S371,S373,S375,T376,K417,S477,T478,E484,Q498,Y505
C1723	G339,S371,S373,S375,T376,K417,S477,T478,Q498,N501,Y505
C1724	G339,S371,S373,S375,T376,K417,T478,E484,F486,Q498,N501,Y505
C1725	G339,S371,S373,S375,T376,K417,T478,E484,Q498,N501,Y505
C1726	G339,S371,S373,S375,T376,L452,E484,N501
C1727	G339,S371,S373,S375,T376,L452,S477,T478,E484,F486,Q498,Y505
C1728	G339,S371,S373,S375,T376,L452,S477,T478,E484,Q498,Y505
C1729	G339,S371,S373,S375,T376,L452,T478,E484,F486,Q498,N501,Y505
C1730	G339,S371,S373,S375,T376,L452,T478,E484,Q498,N501,Y505
C1731	G339,S371,S373,S375,T376,N440
C1732	G339,S371,S373,S375,T376,N440,E484,N501
C1733	G339,S371,S373,S375,T376,N440,L452,E484,N501
C1734	G339,S371,S373,S375,T376,N440,L452,S477,T478,E484,F486,Q498,Y505
C1735	G339,S371,S373,S375,T376,N440,L452,S477,T478,E484,Q498,Y505
C1736	G339,S371,S373,S375,T376,N440,L452,T478,E484,F486,Q498,N501,Y505
C1737	G339,S371,S373,S375,T376,N440,L452,T478,E484,Q498,N501,Y505
C1738	G339,S371,S373,S375,T376,N440,S477,T478,E484,F486,Q498,N501,Y505
C1739	G339,S371,S373,S375,T376,N440,S477,T478,E484,F486,Q498,Y505
C1740	G339,S371,S373,S375,T376,N440,S477,T478,E484,N501
C1741	G339,S371,S373,S375,T376,N440,S477,T478,E484,Q493,Q498,N501,Y505
C1742	G339,S371,S373,S375,T376,N440,S477,T478,E484,Q498,N501,Y505
C1743	G339,S371,S373,S375,T376,N440,S477,T478,E484,Q498,Y505
C1744	G339,S371,S373,S375,T376,N440,S477,T478,Q498,N501,Y505
C1745	G339,S371,S373,S375,T376,N440,T478,E484,F486,Q498,N501,Y505
C1746	G339,S371,S373,S375,T376,N440,T478,E484,Q498,N501,Y505
C1747	G339,S371,S373,S375,T376,N501
C1748	G339,S371,S373,S375,T376,N501,Y505
C1749	G339,S371,S373,S375,T376,Q493,Q498,N501,Y505
C1750	G339,S371,S373,S375,T376,Q498
C1751	G339,S371,S373,S375,T376,Q498,N501
C1752	G339,S371,S373,S375,T376,Q498,N501,Y505
C1753	G339,S371,S373,S375,T376,Q498,Y505
C1754	G339,S371,S373,S375,T376,R408,K417,E484,N501
C1755	G339,S371,S373,S375,T376,R408,K417,L452,E484,N501
C1756	G339,S371,S373,S375,T376,R408,K417,L452,S477,T478,E484,F486,Q498,Y505
C1757	G339,S371,S373,S375,T376,R408,K417,L452,T478,E484,F486,Q498,N501,Y505
C1758	G339,S371,S373,S375,T376,R408,K417,N440,E484,N501
C1759	G339,S371,S373,S375,T376,R408,K417,N440,L452,E484,N501
C1760	G339,S371,S373,S375,T376,R408,K417,N440,L452,S477,T478,E484,Q498,Y505

C1761	G339,S371,S373,S375,T376,R408,K417,N440,L452,T478,E484,Q498,N501,Y505
C1762	G339,S371,S373,S375,T376,R408,K417,N440,S477,T478,E484,N501
C1763	G339,S371,S373,S375,T376,R408,K417,N440,S477,T478,E484,Q493,Q498,N501,Y505
C1764	G339,S371,S373,S375,T376,R408,K417,N440,S477,T478,E484,Q498,N501,Y505
C1765	G339,S371,S373,S375,T376,R408,K417,N440,S477,T478,E484,Q498,Y505
C1766	G339,S371,S373,S375,T376,R408,K417,N440,S477,T478,Q498,N501,Y505
C1767	G339,S371,S373,S375,T376,R408,K417,N440,T478,E484,Q498,N501,Y505
C1768	G339,S371,S373,S375,T376,R408,K417,S477,T478,E484,F486,Q498,N501,Y505
C1769	G339,S371,S373,S375,T376,R408,K417,S477,T478,E484,N501
C1770	G339,S371,S373,S375,T376,R408,K417,S477,T478,E484,Q493,Q498,N501,Y505
C1771	G339,S371,S373,S375,T376,R408,K417,S477,T478,E484,Q498,N501,Y505
C1772	G339,S371,S373,S375,T376,R408,K417,S477,T478,E484,Q498,Y505
C1773	G339,S371,S373,S375,T376,R408,K417,S477,T478,Q498,N501,Y505
C1774	G339,S371,S373,S375,T376,R408,K417,T478,E484,Q498,N501,Y505
C1775	G339,S371,S373,S375,T376,R408,L452,E484,N501
C1776	G339,S371,S373,S375,T376,R408,L452,S477,T478,E484,Q498,Y505
C1777	G339,S371,S373,S375,T376,R408,L452,T478,E484,Q498,N501,Y505
C1778	G339,S371,S373,S375,T376,R408,N440,E484,N501
C1779	G339,S371,S373,S375,T376,R408,N440,S477,T478,E484,N501
C1780	G339,S371,S373,S375,T376,R408,N440,S477,T478,E484,Q493,Q498,N501,Y505
C1781	G339,S371,S373,S375,T376,R408,N440,S477,T478,E484,Q498,N501,Y505
C1782	G339,S371,S373,S375,T376,R408,N440,S477,T478,E484,Q498,Y505
C1783	G339,S371,S373,S375,T376,R408,N440,S477,T478,Q498,N501,Y505
C1784	G339,S371,S373,S375,T376,R408,N440,T478,E484,Q498,N501,Y505
C1785	G339,S371,S373,S375,T376,R408,S477,T478,E484,N501
C1786	G339,S371,S373,S375,T376,R408,S477,T478,E484,Q493,Q498,N501,Y505
C1787	G339,S371,S373,S375,T376,R408,S477,T478,E484,Q498,N501,Y505
C1788	G339,S371,S373,S375,T376,R408,S477,T478,E484,Q498,Y505
C1789	G339,S371,S373,S375,T376,R408,S477,T478,Q498,N501,Y505
C1790	G339,S371,S373,S375,T376,S477,E484,F486,Q498,N501,Y505
C1791	G339,S371,S373,S375,T376,S477,E484,F486,Q498,Y505
C1792	G339,S371,S373,S375,T376,S477,E484,N501
C1793	G339,S371,S373,S375,T376,S477,E484,Q498,N501,Y505
C1794	G339,S371,S373,S375,T376,S477,E484,Q498,Y505
C1795	G339,S371,S373,S375,T376,S477,Q498,N501,Y505
C1796	G339,S371,S373,S375,T376,S477,T478,E484,F486,Q498,N501,Y505
C1797	G339,S371,S373,S375,T376,S477,T478,E484,F486,Q498,Y505
C1798	G339,S371,S373,S375,T376,S477,T478,E484,N501
C1799	G339,S371,S373,S375,T376,S477,T478,E484,Q493,Q498,N501,Y505
C1800	G339,S371,S373,S375,T376,S477,T478,E484,Q498,N501,Y505
C1801	G339,S371,S373,S375,T376,S477,T478,E484,Q498,Y505
C1802	G339,S371,S373,S375,T376,S477,T478,N501
C1803	G339,S371,S373,S375,T376,S477,T478,Q493,Q498,N501,Y505

C1804	G339,S371,S373,S375,T376,S477,T478,Q498,N501,Y505
C1805	G339,S371,S373,S375,T376,S477,T478,Q498,Y505
C1806	G339,S371,S373,S375,T376,T478,E484,F486,Q498
C1807	G339,S371,S373,S375,T376,T478,E484,F486,Q498,N501
C1808	G339,S371,S373,S375,T376,T478,E484,F486,Q498,N501,Y505
C1809	G339,S371,S373,S375,T376,T478,E484,F486,Q498,Y505
C1810	G339,S371,S373,S375,T376,T478,E484,N501
C1811	G339,S371,S373,S375,T376,T478,E484,Q493,Q498,N501,Y505
C1812	G339,S371,S373,S375,T376,T478,E484,Q498
C1813	G339,S371,S373,S375,T376,T478,E484,Q498,N501
C1814	G339,S371,S373,S375,T376,T478,E484,Q498,N501,Y505
C1815	G339,S371,S373,S375,T376,T478,E484,Q498,Y505
C1816	G339,S371,S373,S375,T376,T478,Q498,N501
C1817	G339,S371,S373,S375,T376,T478,Q498,N501,Y505
C1818	G339,S371,S373,S375,T376,Y505
C1819	G339,S371,S373,S375,T478
C1820	G339,S371,S373,S375,T478,E484
C1821	G339,S371,S373,S375,T478,E484,F486,Q498,N501,Y505
C1822	G339,S371,S373,S375,T478,E484,G496
C1823	G339,S371,S373,S375,T478,E484,G496,Y505
C1824	G339,S371,S373,S375,T478,E484,N501
C1825	G339,S371,S373,S375,T478,E484,N501,Y505
C1826	G339,S371,S373,S375,T478,E484,Q493,G496,Q498
C1827	G339,S371,S373,S375,T478,E484,Q493,G496,Q498,Y505
C1828	G339,S371,S373,S375,T478,E484,Q493,Q498
C1829	G339,S371,S373,S375,T478,E484,Q493,Q498,N501
C1830	G339,S371,S373,S375,T478,E484,Q493,Q498,N501,Y505
C1831	G339,S371,S373,S375,T478,E484,Q493,Q498,Y505
C1832	G339,S371,S373,S375,T478,E484,Q498
C1833	G339,S371,S373,S375,T478,E484,Q498,N501
C1834	G339,S371,S373,S375,T478,E484,Q498,N501,Y505
C1835	G339,S371,S373,S375,T478,E484,Q498,Y505
C1836	G339,S371,S373,S375,T478,E484,Y505
C1837	G339,S371,S373,S375,T478,G496
C1838	G339,S371,S373,S375,T478,G496,N501
C1839	G339,S371,S373,S375,T478,G496,Q498
C1840	G339,S371,S373,S375,T478,G496,Q498,Y505
C1841	G339,S371,S373,S375,T478,G496,Y505
C1842	G339,S371,S373,S375,T478,N501
C1843	G339,S371,S373,S375,T478,Q493,G496,Q498
C1844	G339,S371,S373,S375,T478,Q493,G496,Q498,N501
C1845	G339,S371,S373,S375,T478,Q493,G496,Q498,N501,Y505
C1846	G339,S371,S373,S375,T478,Q493,G496,Q498,Y505

C1847	G339,S371,S373,S375,T478,Q493,Q498,N501
C1848	G339,S371,S373,S375,T478,Q493,Q498,N501,Y505
C1849	G339,S371,S373,S375,T478,Q498
C1850	G339,S371,S373,S375,T478,Q498,N501
C1851	G339,S371,S373,S375,T478,Q498,N501,Y505
C1852	G339,S371,S373,S375,T478,Q498,Y505
C1853	G339,S371,S373,S375,T478,Y505
C1854	G339,S371,S373,S375,Y505
C1855	G339,S371,S373,S477,E484,Q493,G496,Q498,N501,Y505
C1856	G339,S371,S373,S477,E484,Q493,G496,Q498,Y505
C1857	G339,S371,S373,S477,E484,Q493,Q498,N501,Y505
C1858	G339,S371,S373,S477,E484,Q493,Q498,Y505
C1859	G339,S371,S373,S477,E484,Q498,N501,Y505
C1860	G339,S371,S373,S477,E484,Q498,Y505
C1861	G339,S371,S373,S477,T478
C1862	G339,S371,S373,S477,T478,E484,F486,Q498,N501,Y505
C1863	G339,S371,S373,S477,T478,E484,F486,Q498,Y505
C1864	G339,S371,S373,S477,T478,E484,N501
C1865	G339,S371,S373,S477,T478,E484,Q493,G496,Q498,Y505
C1866	G339,S371,S373,S477,T478,E484,Q493,Q498,N501,Y505
C1867	G339,S371,S373,S477,T478,E484,Q493,Q498,Y505
C1868	G339,S371,S373,S477,T478,E484,Q498,N501,Y505
C1869	G339,S371,S373,S477,T478,E484,Q498,Y505
C1870	G339,S371,S373,S477,T478,Q493,G496,Q498,N501,Y505
C1871	G339,S371,S373,S477,T478,Q493,Q498,N501,Y505
C1872	G339,S371,S373,S477,T478,Q498,N501,Y505
C1873	G339,S371,S373,T376,D405,E484,N501
C1874	G339,S371,S373,T376,D405,S477,T478,E484,N501
C1875	G339,S371,S373,T376,D405,S477,T478,E484,Q493,Q498,N501,Y505
C1876	G339,S371,S373,T376,D405,S477,T478,E484,Q498,N501,Y505
C1877	G339,S371,S373,T376,D405,S477,T478,E484,Q498,Y505
C1878	G339,S371,S373,T376,D405,S477,T478,Q498,N501,Y505
C1879	G339,S371,S373,T376,D405,T478,E484,Q498,N501,Y505
C1880	G339,S371,S373,T376,E484
C1881	G339,S371,S373,T376,E484,N501
C1882	G339,S371,S373,T376,E484,Q493,Q498
C1883	G339,S371,S373,T376,E484,Q498
C1884	G339,S371,S373,T376,N501
C1885	G339,S371,S373,T376,Q493,Q498,N501,Y505
C1886	G339,S371,S373,T376,Q498
C1887	G339,S371,S373,T376,Q498,N501,Y505
C1888	G339,S371,S373,T376,Q498,Y505
C1889	G339,S371,S373,T376,S477,T478,E484,F486,Q498,N501,Y505

C1890	G339,S371,S373,T376,S477,T478,E484,F486,Q498,Y505
C1891	G339,S371,S373,T376,S477,T478,E484,N501
C1892	G339,S371,S373,T376,S477,T478,E484,Q493,Q498,N501,Y505
C1893	G339,S371,S373,T376,S477,T478,E484,Q498,N501,Y505
C1894	G339,S371,S373,T376,S477,T478,E484,Q498,Y505
C1895	G339,S371,S373,T376,S477,T478,Q498,N501,Y505
C1896	G339,S371,S373,T376,T478,E484,F486,Q498,N501,Y505
C1897	G339,S371,S373,T376,T478,E484,Q498,N501,Y505
C1898	G339,S371,S373,T478
C1899	G339,S371,S373,T478,E484,F486,Q498,N501,Y505
C1900	G339,S371,S373,T478,E484,N501
C1901	G339,S371,S373,T478,E484,Q493,Q498,N501,Y505
C1902	G339,S371,S373,T478,E484,Q493,Q498,Y505
C1903	G339,S371,S373,T478,E484,Q498,N501,Y505
C1904	G339,S371,S373,T478,E484,Q498,Y505
C1905	G339,S371,S373,T478,G496,Q498,Y505
C1906	G339,S371,S373,T478,Q493,Q498,N501,Y505
C1907	G339,S371,S373,T478,Q498,N501
C1908	G339,S371,S373,T478,Q498,N501,Y505
C1909	G339,S371,S373,T478,Q498,Y505
C1910	G339,S371,S375,E484,Q493,G496,Q498,Y505
C1911	G339,S371,S375,G496,N501
C1912	G339,S371,S375,K417,N440,E484,N501
C1913	G339,S371,S375,K417,N440,G446,E484,Q493,G496,Q498,N501,Y505
C1914	G339,S371,S375,K417,N440,G446,S477,E484,Q493,G496,Q498,N501,Y505
C1915	G339,S371,S375,K417,N440,G446,S477,T478
C1916	G339,S371,S375,K417,N440,G446,T478
C1917	G339,S371,S375,K417,N440,G446,T478,E484,Q493,G496,Q498,Y505
C1918	G339,S371,S375,K417,N440,G446,T478,G496,Q498,Y505
C1919	G339,S371,S375,K417,N440,S477,E484,Q493,Q498,N501,Y505
C1920	G339,S371,S375,K417,N440,S477,T478
C1921	G339,S371,S375,K417,N440,S477,T478,E484,N501
C1922	G339,S371,S375,K417,N440,S477,T478,E484,Q493,Q498,N501,Y505
C1923	G339,S371,S375,K417,N440,S477,T478,E484,Q493,Q498,Y505
C1924	G339,S371,S375,K417,N440,S477,T478,E484,Q498,N501,Y505
C1925	G339,S371,S375,K417,N440,S477,T478,E484,Q498,Y505
C1926	G339,S371,S375,K417,N440,S477,T478,Q498,N501,Y505
C1927	G339,S371,S375,K417,N440,T478,E484,N501
C1928	G339,S371,S375,K417,N440,T478,E484,Q493,Q498,N501,Y505
C1929	G339,S371,S375,K417,N440,T478,E484,Q498,N501,Y505
C1930	G339,S371,S375,K417,N440,T478,E484,Q498,Y505
C1931	G339,S371,S375,K417,N440,T478,Q498,N501,Y505
C1932	G339,S371,S375,K417,N440,T478,Q498,Y505

C1933	G339,S371,S375,K417,S477,T478
C1934	G339,S371,S375,K417,S477,T478,E484,Q498,Y505
C1935	G339,S371,S375,K417,T478
C1936	G339,S371,S375,K417,T478,E484,Q498,Y505
C1937	G339,S371,S375,N440,G446,S477,T478,E484,Q493,G496,Q498,Y505
C1938	G339,S371,S375,N440,G446,T478,E484,Q493,G496,Q498,Y505
C1939	G339,S371,S375,N440,S477,T478,E484,Q493,Q498,Y505
C1940	G339,S371,S375,N501
C1941	G339,S371,S375,S477,E484,Q493,Q498,N501,Y505
C1942	G339,S371,S375,S477,E484,Q493,Q498,Y505
C1943	G339,S371,S375,S477,T478
C1944	G339,S371,S375,S477,T478,E484,Q493,Q498,Y505
C1945	G339,S371,S375,S477,T478,Q493,G496,Q498,N501,Y505
C1946	G339,S371,S375,S477,T478,Q493,Q498,N501,Y505
C1947	G339,S371,S375,T376,D405,R408,K417,N440,E484,N501
C1948	G339,S371,S375,T376,D405,R408,K417,N440,L452,E484,N501
C1949	G339,S371,S375,T376,D405,R408,K417,N440,L452,S477,T478,E484,Q498,Y505
C1950	G339,S371,S375,T376,D405,R408,K417,N440,L452,T478,E484,Q498,N501,Y505
C1951	G339,S371,S375,T376,D405,R408,K417,N440,S477,T478,E484,N501
C1952	G339,S371,S375,T376,D405,R408,K417,N440,S477,T478,E484,Q493,Q498,N501,Y505
C1953	G339,S371,S375,T376,D405,R408,K417,N440,S477,T478,E484,Q498,N501,Y505
C1954	G339,S371,S375,T376,D405,R408,K417,N440,S477,T478,E484,Q498,Y505
C1955	G339,S371,S375,T376,D405,R408,K417,N440,S477,T478,Q498,N501,Y505
C1956	G339,S371,S375,T376,D405,R408,K417,N440,T478,E484,Q498,N501,Y505
C1957	G339,S371,S375,T376,D405,R408,K417,S477,T478,E484,Q498,Y505
C1958	G339,S371,S375,T376,E484,N501
C1959	G339,S371,S375,T376,S477,T478,E484,N501
C1960	G339,S371,S375,T376,S477,T478,E484,Q493,Q498,N501,Y505
C1961	G339,S371,S375,T376,S477,T478,E484,Q498,N501,Y505
C1962	G339,S371,S375,T376,S477,T478,E484,Q498,Y505
C1963	G339,S371,S375,T376,S477,T478,Q498,N501,Y505
C1964	G339,S371,S375,T376,T478,E484,Q498,N501,Y505
C1965	G339,S371,S375,T478,Q493,G496,Q498,N501,Y505
C1966	G339,S371,S375,T478,Q498,Y505
C1967	G339,S371,S477,E484,Q493,Q498,N501,Y505
C1968	G339,S371,S477,E484,Q493,Q498,Y505
C1969	G339,S371,S477,E484,Q498,N501,Y505
C1970	G339,S371,S477,E484,Q498,Y505
C1971	G339,S371,S477,T478
C1972	G339,S371,S477,T478,E484,F486,Q498,N501,Y505
C1973	G339,S371,S477,T478,E484,F486,Q498,Y505
C1974	G339,S371,S477,T478,E484,N501
C1975	G339,S371,S477,T478,E484,Q493,Q498,N501,Y505

C1976	G339,S371,S477,T478,E484,Q493,Q498,Y505
C1977	G339,S371,S477,T478,E484,Q498,N501,Y505
C1978	G339,S371,S477,T478,E484,Q498,Y505
C1979	G339,S371,S477,T478,Q493,Q498,N501,Y505
C1980	G339,S371,S477,T478,Q498,N501,Y505
C1981	G339,S371,T478,E484,F486,Q498,N501,Y505
C1982	G339,S371,T478,E484,Q498,N501,Y505
C1983	G339,S371,T478,Q498,Y505
C1984	G339,S373
C1985	G339,S373,D405,E484,N501
C1986	G339,S373,D405,E484,Q493,Q498,N501,Y505
C1987	G339,S373,D405,E484,Q498,N501,Y505
C1988	G339,S373,D405,E484,Q498,Y505
C1989	G339,S373,D405,K417,E484,N501
C1990	G339,S373,D405,K417,N440,E484,N501
C1991	G339,S373,D405,K417,N440,S477,T478,E484,N501
C1992	G339,S373,D405,K417,N440,S477,T478,E484,Q493,Q498,N501,Y505
C1993	G339,S373,D405,K417,N440,S477,T478,E484,Q498,N501,Y505
C1994	G339,S373,D405,K417,N440,S477,T478,E484,Q498,Y505
C1995	G339,S373,D405,K417,N440,S477,T478,Q498,N501,Y505
C1996	G339,S373,D405,K417,N440,T478,E484,Q498,N501,Y505
C1997	G339,S373,D405,K417,S477,T478,E484,N501
C1998	G339,S373,D405,K417,S477,T478,E484,Q493,Q498,N501,Y505
C1999	G339,S373,D405,K417,S477,T478,E484,Q498,N501,Y505
C2000	G339,S373,D405,K417,S477,T478,E484,Q498,Y505
C2001	G339,S373,D405,K417,S477,T478,Q498,N501,Y505
C2002	G339,S373,D405,K417,T478,E484,Q498,N501,Y505
C2003	G339,S373,D405,L452,E484,N501
C2004	G339,S373,D405,L452,S477,T478,E484,Q498,Y505
C2005	G339,S373,D405,L452,T478,E484,Q498,N501,Y505
C2006	G339,S373,D405,Q498,N501,Y505
C2007	G339,S373,D405,R408,E484,N501
C2008	G339,S373,D405,R408,S477,T478,E484,N501
C2009	G339,S373,D405,R408,S477,T478,E484,Q493,Q498,N501,Y505
C2010	G339,S373,D405,R408,S477,T478,E484,Q498,N501,Y505
C2011	G339,S373,D405,R408,S477,T478,E484,Q498,Y505
C2012	G339,S373,D405,R408,S477,T478,Q498,N501,Y505
C2013	G339,S373,D405,R408,T478,E484,Q498,N501,Y505
C2014	G339,S373,D405,S477,T478,E484,F486,Q498,N501,Y505
C2015	G339,S373,D405,S477,T478,E484,F486,Q498,Y505
C2016	G339,S373,D405,S477,T478,E484,N501
C2017	G339,S373,D405,S477,T478,E484,Q493,Q498,N501,Y505
C2018	G339,S373,D405,S477,T478,E484,Q498,N501,Y505

C2019	G339,S373,D405,S477,T478,E484,Q498,Y505
C2020	G339,S373,D405,S477,T478,Q498,N501,Y505
C2021	G339,S373,D405,T478,E484,F486,Q498,N501,Y505
C2022	G339,S373,D405,T478,E484,Q498,N501,Y505
C2023	G339,S373,E484,F486,Q498,N501,Y505
C2024	G339,S373,E484,F486,Q498,Y505
C2025	G339,S373,E484,N501
C2026	G339,S373,E484,Q493,G496,Q498,N501,Y505
C2027	G339,S373,E484,Q493,G496,Q498,Y505
C2028	G339,S373,E484,Q493,Q498
C2029	G339,S373,E484,Q493,Q498,N501
C2030	G339,S373,E484,Q493,Q498,N501,Y505
C2031	G339,S373,E484,Q493,Q498,Y505
C2032	G339,S373,E484,Q498
C2033	G339,S373,E484,Q498,N501
C2034	G339,S373,E484,Q498,N501,Y505
C2035	G339,S373,E484,Q498,Y505
C2036	G339,S373,G496,N501
C2037	G339,S373,G496,Q498,Y505
C2038	G339,S373,K417,N440,E484,N501
C2039	G339,S373,K417,N440,S477,E484,Q493,Q498,N501,Y505
C2040	G339,S373,K417,N440,S477,T478
C2041	G339,S373,K417,N440,S477,T478,E484,N501
C2042	G339,S373,K417,N440,S477,T478,E484,Q493,Q498,N501,Y505
C2043	G339,S373,K417,N440,S477,T478,E484,Q493,Q498,Y505
C2044	G339,S373,K417,N440,S477,T478,E484,Q498,N501,Y505
C2045	G339,S373,K417,N440,S477,T478,E484,Q498,Y505
C2046	G339,S373,K417,N440,S477,T478,Q498,N501,Y505
C2047	G339,S373,K417,N440,T478,E484,Q498,N501,Y505
C2048	G339,S373,K417,N440,T478,Q498,Y505
C2049	G339,S373,K417,S477,E484,Q493,Q498,N501,Y505
C2050	G339,S373,K417,S477,T478
C2051	G339,S373,K417,S477,T478,E484,Q493,Q498,Y505
C2052	G339,S373,K417,S477,T478,E484,Q498,Y505
C2053	G339,S373,K417,T478,Q498,Y505
C2054	G339,S373,L452,E484,N501
C2055	G339,S373,L452,S477,T478,E484,F486,Q498,Y505
C2056	G339,S373,L452,S477,T478,E484,Q498,Y505
C2057	G339,S373,L452,T478,E484,F486,Q498,N501,Y505
C2058	G339,S373,L452,T478,E484,Q498,N501,Y505
C2059	G339,S373,N440,E484,N501
C2060	G339,S373,N440,G446,S477,E484,Q493,G496,Q498,N501,Y505
C2061	G339,S373,N440,G446,S477,E484,Q493,N501,Y505

C2062	G339,S373,N440,G446,S477,T478
C2063	G339,S373,N440,G446,S477,T478,E484,Q493,G496,Q498,Y505
C2064	G339,S373,N440,G446,S477,T478,E484,Q493,Y505
C2065	G339,S373,N440,G446,T478,G496,Q498,Y505
C2066	G339,S373,N440,G446,T478,Y505
C2067	G339,S373,N440,S477,E484,Q493,Q498,N501,Y505
C2068	G339,S373,N440,S477,T478
C2069	G339,S373,N440,S477,T478,E484,N501
C2070	G339,S373,N440,S477,T478,E484,N501,Y505
C2071	G339,S373,N440,S477,T478,E484,Q493,N501,Y505
C2072	G339,S373,N440,S477,T478,E484,Q493,Q498,N501,Y505
C2073	G339,S373,N440,S477,T478,E484,Q493,Q498,Y505
C2074	G339,S373,N440,S477,T478,E484,Q498,N501,Y505
C2075	G339,S373,N440,S477,T478,E484,Q498,Y505
C2076	G339,S373,N440,S477,T478,E484,Y505
C2077	G339,S373,N440,S477,T478,N501,Y505
C2078	G339,S373,N440,S477,T478,Q498,N501,Y505
C2079	G339,S373,N440,T478,E484,N501,Y505
C2080	G339,S373,N440,T478,E484,Q498,N501,Y505
C2081	G339,S373,N440,T478,Q498,Y505
C2082	G339,S373,N501
C2083	G339,S373,Q493
C2084	G339,S373,Q493,G496,Q498,N501,Y505
C2085	G339,S373,Q493,N501
C2086	G339,S373,Q493,Q498,N501
C2087	G339,S373,Q493,Q498,N501,Y505
C2088	G339,S373,Q498
C2089	G339,S373,Q498,N501
C2090	G339,S373,Q498,N501,Y505
C2091	G339,S373,Q498,Y505
C2092	G339,S373,R408,K417,N440,E484,N501
C2093	G339,S373,R408,K417,N440,S477,T478,E484,N501
C2094	G339,S373,R408,K417,N440,S477,T478,E484,Q493,Q498,N501,Y505
C2095	G339,S373,R408,K417,N440,S477,T478,E484,Q498,N501,Y505
C2096	G339,S373,R408,K417,N440,S477,T478,E484,Q498,Y505
C2097	G339,S373,R408,K417,N440,S477,T478,Q498,N501,Y505
C2098	G339,S373,R408,K417,N440,T478,E484,Q498,N501,Y505
C2099	G339,S373,R408,K417,S477,T478,E484,Q498,Y505
C2100	G339,S373,S375
C2101	G339,S373,S375,D405,E484,N501
C2102	G339,S373,S375,D405,S477,T478,E484,N501
C2103	G339,S373,S375,D405,S477,T478,E484,Q493,Q498,N501,Y505
C2104	G339,S373,S375,D405,S477,T478,E484,Q498,N501,Y505

C2105	G339,S373,S375,D405,S477,T478,E484,Q498,Y505
C2106	G339,S373,S375,D405,S477,T478,Q498,N501,Y505
C2107	G339,S373,S375,D405,T478,E484,Q498,N501,Y505
C2108	G339,S373,S375,E484
C2109	G339,S373,S375,E484,F486,Q498,N501,Y505
C2110	G339,S373,S375,E484,F486,Q498,Y505
C2111	G339,S373,S375,E484,N501
C2112	G339,S373,S375,E484,Q493,G496,Q498,N501,Y505
C2113	G339,S373,S375,E484,Q493,G496,Q498,Y505
C2114	G339,S373,S375,E484,Q493,Q498
C2115	G339,S373,S375,E484,Q493,Q498,N501
C2116	G339,S373,S375,E484,Q493,Q498,N501,Y505
C2117	G339,S373,S375,E484,Q493,Q498,Y505
C2118	G339,S373,S375,E484,Q498
C2119	G339,S373,S375,E484,Q498,N501
C2120	G339,S373,S375,E484,Q498,N501,Y505
C2121	G339,S373,S375,E484,Q498,Y505
C2122	G339,S373,S375,G446,S477,E484,Q493,G496,Q498,N501,Y505
C2123	G339,S373,S375,G446,S477,T478
C2124	G339,S373,S375,G446,S477,T478,E484,Q493,G496,Q498,Y505
C2125	G339,S373,S375,G446,T478,G496,Q498,Y505
C2126	G339,S373,S375,G496,N501
C2127	G339,S373,S375,G496,Q498
C2128	G339,S373,S375,G496,Q498,N501
C2129	G339,S373,S375,G496,Q498,Y505
C2130	G339,S373,S375,K417,N440,E484,N501
C2131	G339,S373,S375,K417,N440,G446,S477,E484,Q493,G496,Q498,N501,Y505
C2132	G339,S373,S375,K417,N440,G446,S477,T478
C2133	G339,S373,S375,K417,N440,G446,S477,T478,E484,Q493,G496,Q498,Y505
C2134	G339,S373,S375,K417,N440,G446,T478,G496,Q498,Y505
C2135	G339,S373,S375,K417,N440,S477,E484,Q493,Q498,N501,Y505
C2136	G339,S373,S375,K417,N440,S477,E484,Q498,N501,Y505
C2137	G339,S373,S375,K417,N440,S477,T478
C2138	G339,S373,S375,K417,N440,S477,T478,E484,N501
C2139	G339,S373,S375,K417,N440,S477,T478,E484,Q493,Q498,N501,Y505
C2140	G339,S373,S375,K417,N440,S477,T478,E484,Q493,Q498,Y505
C2141	G339,S373,S375,K417,N440,S477,T478,E484,Q498,N501,Y505
C2142	G339,S373,S375,K417,N440,S477,T478,E484,Q498,Y505
C2143	G339,S373,S375,K417,N440,S477,T478,Q498,N501,Y505
C2144	G339,S373,S375,K417,N440,T478,E484,Q498,N501,Y505
C2145	G339,S373,S375,K417,N440,T478,Q498,Y505
C2146	G339,S373,S375,K417,S477,E484,Q493,Q498,N501,Y505
C2147	G339,S373,S375,K417,S477,E484,Q498,N501,Y505

C2148	G339,S373,S375,K417,S477,T478
C2149	G339,S373,S375,K417,S477,T478,E484,Q493,Q498,Y505
C2150	G339,S373,S375,K417,S477,T478,E484,Q498,Y505
C2151	G339,S373,S375,K417,T478,Q498,Y505
C2152	G339,S373,S375,N440,E484,N501
C2153	G339,S373,S375,N440,G446,E484,Q493,G496,Q498,N501
C2154	G339,S373,S375,N440,G446,S477,E484,Q493,G496,Q498,N501,Y505
C2155	G339,S373,S375,N440,G446,S477,T478
C2156	G339,S373,S375,N440,G446,S477,T478,E484,Q493,G496,Q498,Y505
C2157	G339,S373,S375,N440,G446,T478
C2158	G339,S373,S375,N440,G446,T478,E484,Q493,G496,Q498
C2159	G339,S373,S375,N440,G446,T478,G496,Q498
C2160	G339,S373,S375,N440,G446,T478,G496,Q498,Y505
C2161	G339,S373,S375,N440,S477,E484,Q493,Q498,N501,Y505
C2162	G339,S373,S375,N440,S477,E484,Q498,N501,Y505
C2163	G339,S373,S375,N440,S477,T478
C2164	G339,S373,S375,N440,S477,T478,E484,N501
C2165	G339,S373,S375,N440,S477,T478,E484,Q493,Q498,N501,Y505
C2166	G339,S373,S375,N440,S477,T478,E484,Q493,Q498,Y505
C2167	G339,S373,S375,N440,S477,T478,E484,Q498,N501,Y505
C2168	G339,S373,S375,N440,S477,T478,E484,Q498,Y505
C2169	G339,S373,S375,N440,S477,T478,Q498,N501,Y505
C2170	G339,S373,S375,N440,T478,E484,N501
C2171	G339,S373,S375,N440,T478,E484,Q493,Q498,N501
C2172	G339,S373,S375,N440,T478,E484,Q498
C2173	G339,S373,S375,N440,T478,E484,Q498,N501
C2174	G339,S373,S375,N440,T478,E484,Q498,N501,Y505
C2175	G339,S373,S375,N440,T478,Q498,N501
C2176	G339,S373,S375,N440,T478,Q498,Y505
C2177	G339,S373,S375,N501
C2178	G339,S373,S375,N501,Y505
C2179	G339,S373,S375,Q493,G496,Q498,N501,Y505
C2180	G339,S373,S375,Q493,G496,Q498,Y505
C2181	G339,S373,S375,Q493,Q498,N501
C2182	G339,S373,S375,Q493,Q498,N501,Y505
C2183	G339,S373,S375,Q498
C2184	G339,S373,S375,Q498,N501
C2185	G339,S373,S375,Q498,N501,Y505
C2186	G339,S373,S375,Q498,Y505
C2187	G339,S373,S375,S477,E484
C2188	G339,S373,S375,S477,E484,N501
C2189	G339,S373,S375,S477,E484,Q493,G496,Q498,N501,Y505
C2190	G339,S373,S375,S477,E484,Q493,G496,Q498,Y505

C2191	G339,S373,S375,S477,E484,Q493,Q498,N501,Y505
C2192	G339,S373,S375,S477,E484,Q493,Q498,Y505
C2193	G339,S373,S375,S477,E484,Q498
C2194	G339,S373,S375,S477,E484,Q498,N501
C2195	G339,S373,S375,S477,E484,Q498,N501,Y505
C2196	G339,S373,S375,S477,E484,Q498,Y505
C2197	G339,S373,S375,S477,Q493,G496,Q498,N501,Y505
C2198	G339,S373,S375,S477,Q493,G496,Q498,Y505
C2199	G339,S373,S375,S477,T478
C2200	G339,S373,S375,S477,T478,E484
C2201	G339,S373,S375,S477,T478,E484,F486,Q498,N501,Y505
C2202	G339,S373,S375,S477,T478,E484,F486,Q498,Y505
C2203	G339,S373,S375,S477,T478,E484,N501
C2204	G339,S373,S375,S477,T478,E484,Q493,G496,Q498,Y505
C2205	G339,S373,S375,S477,T478,E484,Q493,Q498,N501,Y505
C2206	G339,S373,S375,S477,T478,E484,Q493,Q498,Y505
C2207	G339,S373,S375,S477,T478,E484,Q498
C2208	G339,S373,S375,S477,T478,E484,Q498,N501,Y505
C2209	G339,S373,S375,S477,T478,E484,Q498,Y505
C2210	G339,S373,S375,S477,T478,N501
C2211	G339,S373,S375,S477,T478,Q493,G496,Q498,N501,Y505
C2212	G339,S373,S375,S477,T478,Q493,G496,Q498,Y505
C2213	G339,S373,S375,S477,T478,Q493,Q498,N501,Y505
C2214	G339,S373,S375,S477,T478,Q498,N501
C2215	G339,S373,S375,S477,T478,Q498,N501,Y505
C2216	G339,S373,S375,S477,T478,Q498,Y505
C2217	G339,S373,S375,T376
C2218	G339,S373,S375,T376,D405,E484,F486,Q498,N501,Y505
C2219	G339,S373,S375,T376,D405,E484,F486,Q498,Y505
C2220	G339,S373,S375,T376,D405,E484,N501
C2221	G339,S373,S375,T376,D405,E484,Q498,N501,Y505
C2222	G339,S373,S375,T376,D405,E484,Q498,Y505
C2223	G339,S373,S375,T376,D405,K417,E484,N501
C2224	G339,S373,S375,T376,D405,K417,N440,E484,N501
C2225	G339,S373,S375,T376,D405,K417,N440,L452,E484,N501
C2226	G339,S373,S375,T376,D405,K417,N440,L452,S477,T478,E484,F486,Q498,Y505
C2227	G339,S373,S375,T376,D405,K417,N440,L452,T478,E484,F486,Q498,N501,Y505
C2228	G339,S373,S375,T376,D405,K417,N440,S477,T478,E484,F486,Q498,N501,Y505
C2229	G339,S373,S375,T376,D405,K417,N440,S477,T478,E484,F486,Q498,Y505
C2230	G339,S373,S375,T376,D405,K417,N440,S477,T478,E484,N501
C2231	G339,S373,S375,T376,D405,K417,N440,S477,T478,E484,Q493,Q498,N501,Y505
C2232	G339,S373,S375,T376,D405,K417,N440,S477,T478,E484,Q498,N501,Y505
C2233	G339,S373,S375,T376,D405,K417,N440,S477,T478,E484,Q498,Y505

C2234	G339,S373,S375,T376,D405,K417,N440,S477,T478,Q498,N501,Y505
C2235	G339,S373,S375,T376,D405,K417,N440,T478,E484,F486,Q498,N501,Y505
C2236	G339,S373,S375,T376,D405,K417,N440,T478,E484,Q498,N501,Y505
C2237	G339,S373,S375,T376,D405,K417,S477,T478,E484,F486,Q498,N501,Y505
C2238	G339,S373,S375,T376,D405,K417,S477,T478,E484,F486,Q498,Y505
C2239	G339,S373,S375,T376,D405,K417,S477,T478,E484,N501
C2240	G339,S373,S375,T376,D405,K417,S477,T478,E484,Q493,Q498,N501,Y505
C2241	G339,S373,S375,T376,D405,K417,S477,T478,E484,Q498,N501,Y505
C2242	G339,S373,S375,T376,D405,K417,S477,T478,E484,Q498,Y505
C2243	G339,S373,S375,T376,D405,K417,S477,T478,Q498,N501,Y505
C2244	G339,S373,S375,T376,D405,K417,T478,E484,F486,Q498,N501,Y505
C2245	G339,S373,S375,T376,D405,K417,T478,E484,Q498,N501,Y505
C2246	G339,S373,S375,T376,D405,L452,E484,N501
C2247	G339,S373,S375,T376,D405,L452,S477,T478,E484,F486,Q498,Y505
C2248	G339,S373,S375,T376,D405,L452,S477,T478,E484,Q498,Y505
C2249	G339,S373,S375,T376,D405,L452,T478,E484,F486,Q498,N501,Y505
C2250	G339,S373,S375,T376,D405,L452,T478,E484,Q498,N501,Y505
C2251	G339,S373,S375,T376,D405,N440,E484,N501
C2252	G339,S373,S375,T376,D405,N440,L452,E484,N501
C2253	G339,S373,S375,T376,D405,N440,L452,S477,T478,E484,F486,Q498,Y505
C2254	G339,S373,S375,T376,D405,N440,L452,S477,T478,E484,Q498,Y505
C2255	G339,S373,S375,T376,D405,N440,L452,T478,E484,F486,Q498,N501,Y505
C2256	G339,S373,S375,T376,D405,N440,L452,T478,E484,Q498,N501,Y505
C2257	G339,S373,S375,T376,D405,N440,S477,T478,E484,F486,Q498,N501,Y505
C2258	G339,S373,S375,T376,D405,N440,S477,T478,E484,N501
C2259	G339,S373,S375,T376,D405,N440,S477,T478,E484,Q493,Q498,N501,Y505
C2260	G339,S373,S375,T376,D405,N440,S477,T478,E484,Q498,N501,Y505
C2261	G339,S373,S375,T376,D405,N440,S477,T478,E484,Q498,Y505
C2262	G339,S373,S375,T376,D405,N440,S477,T478,Q498,N501,Y505
C2263	G339,S373,S375,T376,D405,N440,T478,E484,Q498,N501,Y505
C2264	G339,S373,S375,T376,D405,Q498,N501,Y505
C2265	G339,S373,S375,T376,D405,R408,K417,E484,N501
C2266	G339,S373,S375,T376,D405,R408,K417,N440,E484,N501
C2267	G339,S373,S375,T376,D405,R408,K417,N440,L452,E484,N501
C2268	G339,S373,S375,T376,D405,R408,K417,N440,L452,S477,T478,E484,F486,Q498,Y505
C2269	G339,S373,S375,T376,D405,R408,K417,N440,L452,S477,T478,E484,Q498,Y505
C2270	G339,S373,S375,T376,D405,R408,K417,N440,L452,T478,E484,F486,Q498,N501,Y505
C2271	G339,S373,S375,T376,D405,R408,K417,N440,L452,T478,E484,Q498,N501,Y505
C2272	G339,S373,S375,T376,D405,R408,K417,N440,S477,T478,E484,F486,Q498,N501,Y505
C2273	G339,S373,S375,T376,D405,R408,K417,N440,S477,T478,E484,N501
C2274	G339,S373,S375,T376,D405,R408,K417,N440,S477,T478,E484,Q493,Q498,N501,Y505
C2275	G339,S373,S375,T376,D405,R408,K417,N440,S477,T478,E484,Q498,N501,Y505
C2276	G339,S373,S375,T376,D405,R408,K417,N440,S477,T478,E484,Q498,Y505

C2277	G339,S373,S375,T376,D405,R408,K417,N440,S477,T478,Q498,N501,Y505
C2278	G339,S373,S375,T376,D405,R408,K417,N440,T478,E484,Q498,N501,Y505
C2279	G339,S373,S375,T376,D405,R408,K417,S477,T478,E484,N501
C2280	G339,S373,S375,T376,D405,R408,K417,S477,T478,E484,Q493,Q498,N501,Y505
C2281	G339,S373,S375,T376,D405,R408,K417,S477,T478,E484,Q498,N501,Y505
C2282	G339,S373,S375,T376,D405,R408,K417,S477,T478,E484,Q498,Y505
C2283	G339,S373,S375,T376,D405,R408,K417,S477,T478,Q498,N501,Y505
C2284	G339,S373,S375,T376,D405,R408,K417,T478,E484,Q498,N501,Y505
C2285	G339,S373,S375,T376,D405,R408,N440,E484,N501
C2286	G339,S373,S375,T376,D405,R408,N440,S477,T478,E484,N501
C2287	G339,S373,S375,T376,D405,R408,N440,S477,T478,E484,Q493,Q498,N501,Y505
C2288	G339,S373,S375,T376,D405,R408,N440,S477,T478,E484,Q498,N501,Y505
C2289	G339,S373,S375,T376,D405,R408,N440,S477,T478,E484,Q498,Y505
C2290	G339,S373,S375,T376,D405,R408,N440,S477,T478,Q498,N501,Y505
C2291	G339,S373,S375,T376,D405,R408,N440,T478,E484,Q498,N501,Y505
C2292	G339,S373,S375,T376,D405,R408,S477,T478,E484,Q498,Y505
C2293	G339,S373,S375,T376,D405,S477,T478,E484,F486,Q498
C2294	G339,S373,S375,T376,D405,S477,T478,E484,F486,Q498,N501
C2295	G339,S373,S375,T376,D405,S477,T478,E484,F486,Q498,N501,Y505
C2296	G339,S373,S375,T376,D405,S477,T478,E484,F486,Q498,Y505
C2297	G339,S373,S375,T376,D405,S477,T478,E484,N501
C2298	G339,S373,S375,T376,D405,S477,T478,E484,Q493,Q498,N501,Y505
C2299	G339,S373,S375,T376,D405,S477,T478,E484,Q498
C2300	G339,S373,S375,T376,D405,S477,T478,E484,Q498,N501
C2301	G339,S373,S375,T376,D405,S477,T478,E484,Q498,N501,Y505
C2302	G339,S373,S375,T376,D405,S477,T478,E484,Q498,Y505
C2303	G339,S373,S375,T376,D405,S477,T478,Q498,N501
C2304	G339,S373,S375,T376,D405,S477,T478,Q498,N501,Y505
C2305	G339,S373,S375,T376,D405,T478,E484,F486,Q498,N501
C2306	G339,S373,S375,T376,D405,T478,E484,F486,Q498,N501,Y505
C2307	G339,S373,S375,T376,D405,T478,E484,F486,Q498,Y505
C2308	G339,S373,S375,T376,D405,T478,E484,N501
C2309	G339,S373,S375,T376,D405,T478,E484,Q498,N501,Y505
C2310	G339,S373,S375,T376,D405,T478,E484,Q498,Y505
C2311	G339,S373,S375,T376,D405,T478,Q498,N501,Y505
C2312	G339,S373,S375,T376,E484
C2313	G339,S373,S375,T376,E484,F486
C2314	G339,S373,S375,T376,E484,F486,Q498
C2315	G339,S373,S375,T376,E484,F486,Q498,N501
C2316	G339,S373,S375,T376,E484,F486,Q498,N501,Y505
C2317	G339,S373,S375,T376,E484,F486,Q498,Y505
C2318	G339,S373,S375,T376,E484,N501
C2319	G339,S373,S375,T376,E484,Q493,Q498,N501

C2320	G339,S373,S375,T376,E484,Q493,Q498,N501,Y505
C2321	G339,S373,S375,T376,E484,Q498
C2322	G339,S373,S375,T376,E484,Q498,N501
C2323	G339,S373,S375,T376,E484,Q498,N501,Y505
C2324	G339,S373,S375,T376,E484,Q498,Y505
C2325	G339,S373,S375,T376,F486,Q498,N501,Y505
C2326	G339,S373,S375,T376,F486,Q498,Y505
C2327	G339,S373,S375,T376,K417,E484,N501
C2328	G339,S373,S375,T376,K417,N440,E484,N501
C2329	G339,S373,S375,T376,K417,N440,S477,T478,E484,N501
C2330	G339,S373,S375,T376,K417,N440,S477,T478,E484,Q493,Q498,N501,Y505
C2331	G339,S373,S375,T376,K417,N440,S477,T478,E484,Q498,N501,Y505
C2332	G339,S373,S375,T376,K417,N440,S477,T478,E484,Q498,Y505
C2333	G339,S373,S375,T376,K417,N440,S477,T478,Q498,N501,Y505
C2334	G339,S373,S375,T376,K417,N440,T478,E484,Q498,N501,Y505
C2335	G339,S373,S375,T376,K417,S477,T478,E484,F486,Q498,N501,Y505
C2336	G339,S373,S375,T376,K417,S477,T478,E484,F486,Q498,Y505
C2337	G339,S373,S375,T376,K417,S477,T478,E484,N501
C2338	G339,S373,S375,T376,K417,S477,T478,E484,Q493,Q498,N501,Y505
C2339	G339,S373,S375,T376,K417,S477,T478,E484,Q498,N501,Y505
C2340	G339,S373,S375,T376,K417,S477,T478,E484,Q498,Y505
C2341	G339,S373,S375,T376,K417,S477,T478,Q498,N501,Y505
C2342	G339,S373,S375,T376,K417,T478,E484,F486,Q498,N501,Y505
C2343	G339,S373,S375,T376,K417,T478,E484,Q498,N501,Y505
C2344	G339,S373,S375,T376,L452,E484,N501
C2345	G339,S373,S375,T376,L452,S477,T478,E484,F486,Q498,Y505
C2346	G339,S373,S375,T376,L452,S477,T478,E484,Q498,Y505
C2347	G339,S373,S375,T376,L452,T478,E484,F486,Q498,N501,Y505
C2348	G339,S373,S375,T376,L452,T478,E484,Q498,N501,Y505
C2349	G339,S373,S375,T376,N440,E484,N501
C2350	G339,S373,S375,T376,N440,L452,E484,N501
C2351	G339,S373,S375,T376,N440,L452,S477,T478,E484,F486,Q498,Y505
C2352	G339,S373,S375,T376,N440,L452,S477,T478,E484,Q498,Y505
C2353	G339,S373,S375,T376,N440,L452,T478,E484,F486,Q498,N501,Y505
C2354	G339,S373,S375,T376,N440,L452,T478,E484,Q498,N501,Y505
C2355	G339,S373,S375,T376,N440,S477,T478,E484,F486,Q498,N501,Y505
C2356	G339,S373,S375,T376,N440,S477,T478,E484,N501
C2357	G339,S373,S375,T376,N440,S477,T478,E484,Q493,Q498,N501,Y505
C2358	G339,S373,S375,T376,N440,S477,T478,E484,Q498,N501,Y505
C2359	G339,S373,S375,T376,N440,S477,T478,E484,Q498,Y505
C2360	G339,S373,S375,T376,N440,S477,T478,Q498,N501,Y505
C2361	G339,S373,S375,T376,N440,T478,E484,Q498,N501,Y505
C2362	G339,S373,S375,T376,N501

C2363	G339,S373,S375,T376,N501,Y505
C2364	G339,S373,S375,T376,Q498,N501
C2365	G339,S373,S375,T376,Q498,N501,Y505
C2366	G339,S373,S375,T376,Q498,Y505
C2367	G339,S373,S375,T376,R408,K417,E484,N501
C2368	G339,S373,S375,T376,R408,K417,L452,E484,N501
C2369	G339,S373,S375,T376,R408,K417,L452,S477,T478,E484,F486,Q498,Y505
C2370	G339,S373,S375,T376,R408,K417,L452,T478,E484,F486,Q498,N501,Y505
C2371	G339,S373,S375,T376,R408,K417,S477,T478,E484,F486,Q498,N501,Y505
C2372	G339,S373,S375,T376,R408,K417,S477,T478,E484,F486,Q498,Y505
C2373	G339,S373,S375,T376,R408,K417,S477,T478,E484,N501
C2374	G339,S373,S375,T376,R408,K417,S477,T478,E484,Q498,N501,Y505
C2375	G339,S373,S375,T376,R408,K417,S477,T478,E484,Q498,Y505
C2376	G339,S373,S375,T376,R408,K417,S477,T478,Q498,N501,Y505
C2377	G339,S373,S375,T376,R408,K417,T478,E484,F486,Q498,N501,Y505
C2378	G339,S373,S375,T376,S477,T478,E484
C2379	G339,S373,S375,T376,S477,T478,E484,F486,Q498,N501,Y505
C2380	G339,S373,S375,T376,S477,T478,E484,F486,Q498,Y505
C2381	G339,S373,S375,T376,S477,T478,E484,N501
C2382	G339,S373,S375,T376,S477,T478,E484,Q493,Q498,N501,Y505
C2383	G339,S373,S375,T376,S477,T478,E484,Q498,N501,Y505
C2384	G339,S373,S375,T376,S477,T478,E484,Q498,Y505
C2385	G339,S373,S375,T376,S477,T478,N501
C2386	G339,S373,S375,T376,S477,T478,Q498,N501,Y505
C2387	G339,S373,S375,T376,T478,E484,F486,Q498
C2388	G339,S373,S375,T376,T478,E484,F486,Q498,N501
C2389	G339,S373,S375,T376,T478,E484,F486,Q498,N501,Y505
C2390	G339,S373,S375,T376,T478,E484,F486,Q498,Y505
C2391	G339,S373,S375,T376,T478,E484,N501
C2392	G339,S373,S375,T376,T478,E484,Q493,Q498,N501,Y505
C2393	G339,S373,S375,T376,T478,E484,Q498
C2394	G339,S373,S375,T376,T478,E484,Q498,N501
C2395	G339,S373,S375,T376,T478,E484,Q498,N501,Y505
C2396	G339,S373,S375,T376,T478,E484,Q498,Y505
C2397	G339,S373,S375,T376,T478,Q498,N501
C2398	G339,S373,S375,T376,T478,Q498,N501,Y505
C2399	G339,S373,S375,T376,Y505
C2400	G339,S373,S375,T478
C2401	G339,S373,S375,T478,E484,F486,Q498,N501,Y505
C2402	G339,S373,S375,T478,E484,Q493,Q498,Y505
C2403	G339,S373,S375,T478,E484,Q498
C2404	G339,S373,S375,T478,E484,Q498,N501,Y505
C2405	G339,S373,S375,T478,E484,Q498,Y505

C2406	G339,S373,S375,T478,G496,Q498,Y505
C2407	G339,S373,S375,T478,Q493,G496,Q498,N501
C2408	G339,S373,S375,T478,Q493,Q498,N501,Y505
C2409	G339,S373,S375,T478,Q498
C2410	G339,S373,S375,T478,Q498,N501
C2411	G339,S373,S375,T478,Q498,N501,Y505
C2412	G339,S373,S375,T478,Q498,Y505
C2413	G339,S373,S375,Y505
C2414	G339,S373,S477,E484,Q493,G496,Q498,N501,Y505
C2415	G339,S373,S477,E484,Q493,G496,Q498,Y505
C2416	G339,S373,S477,E484,Q493,Q498,N501,Y505
C2417	G339,S373,S477,E484,Q493,Q498,Y505
C2418	G339,S373,S477,E484,Q493,Y505
C2419	G339,S373,S477,E484,Q498,N501,Y505
C2420	G339,S373,S477,E484,Q498,Y505
C2421	G339,S373,S477,T478
C2422	G339,S373,S477,T478,E484,F486,Q498,N501,Y505
C2423	G339,S373,S477,T478,E484,F486,Q498,Y505
C2424	G339,S373,S477,T478,E484,N501
C2425	G339,S373,S477,T478,E484,Q493,G496,Q498,Y505
C2426	G339,S373,S477,T478,E484,Q493,Q498,N501,Y505
C2427	G339,S373,S477,T478,E484,Q493,Q498,Y505
C2428	G339,S373,S477,T478,E484,Q498,N501,Y505
C2429	G339,S373,S477,T478,E484,Q498,Y505
C2430	G339,S373,S477,T478,E484,Y505
C2431	G339,S373,S477,T478,Q493,G496,Q498,N501,Y505
C2432	G339,S373,S477,T478,Q493,N501,Y505
C2433	G339,S373,S477,T478,Q493,Q498,N501,Y505
C2434	G339,S373,S477,T478,Q498,N501,Y505
C2435	G339,S373,T376,D405,R408,K417,N440,L452,E484,N501
C2436	G339,S373,T376,D405,R408,K417,N440,L452,S477,T478,E484,Q498,Y505
C2437	G339,S373,T376,D405,R408,K417,N440,L452,T478,E484,Q498,N501,Y505
C2438	G339,S373,T376,D405,R408,K417,N440,S477,T478,E484,N501
C2439	G339,S373,T376,D405,R408,K417,N440,S477,T478,E484,Q493,Q498,N501,Y505
C2440	G339,S373,T376,D405,R408,K417,N440,S477,T478,E484,Q498,N501,Y505
C2441	G339,S373,T376,D405,R408,K417,N440,S477,T478,Q498,N501,Y505
C2442	G339,S373,T376,D405,R408,K417,S477,T478,E484,Q498,Y505
C2443	G339,S373,T376,E484,N501
C2444	G339,S373,T376,S477,T478,E484,F486,Q498,N501,Y505
C2445	G339,S373,T376,S477,T478,E484,F486,Q498,Y505
C2446	G339,S373,T376,S477,T478,E484,N501
C2447	G339,S373,T376,S477,T478,E484,Q493,Q498,N501,Y505
C2448	G339,S373,T376,S477,T478,E484,Q498,N501,Y505

C2449	G339,S373,T376,S477,T478,E484,Q498,Y505
C2450	G339,S373,T376,S477,T478,Q498,N501,Y505
C2451	G339,S373,T376,T478,E484,F486,Q498,N501,Y505
C2452	G339,S373,T376,T478,E484,Q498,N501,Y505
C2453	G339,S373,T478
C2454	G339,S373,T478,E484,F486,Q498,N501,Y505
C2455	G339,S373,T478,E484,F486,Q498,Y505
C2456	G339,S373,T478,E484,N501
C2457	G339,S373,T478,E484,Q498,N501,Y505
C2458	G339,S373,T478,E484,Q498,Y505
C2459	G339,S373,T478,G496,Q498,Y505
C2460	G339,S373,T478,Q498,N501,Y505
C2461	G339,S373,T478,Q498,Y505
C2462	G339,S375
C2463	G339,S375,E484,N501
C2464	G339,S375,E484,Q493,G496,Q498,Y505
C2465	G339,S375,E484,Q493,Q498,N501,Y505
C2466	G339,S375,E484,Q493,Q498,Y505
C2467	G339,S375,E484,Q498,N501,Y505
C2468	G339,S375,E484,Q498,Y505
C2469	G339,S375,G446
C2470	G339,S375,G446,E484,Q493,G496,Q498,N501,Y505
C2471	G339,S375,G446,E484,Q493,G496,Q498,Y505
C2472	G339,S375,G446,G496,Q498,Y505
C2473	G339,S375,G446,S477,E484,Q493,G496,Q498,N501,Y505
C2474	G339,S375,G446,S477,T478
C2475	G339,S375,G446,S477,T478,E484,Q493,G496,Q498,Y505
C2476	G339,S375,G446,T478,G496,Q498,Y505
C2477	G339,S375,G496,N501
C2478	G339,S375,K417,E484,N501
C2479	G339,S375,K417,N440,E484,N501
C2480	G339,S375,K417,N440,G446,E484,Q493,G496,Q498,N501,Y505
C2481	G339,S375,K417,N440,G446,S477,E484,Q493,G496,Q498,N501,Y505
C2482	G339,S375,K417,N440,G446,S477,N501
C2483	G339,S375,K417,N440,G446,S477,T478
C2484	G339,S375,K417,N440,G446,S477,T478,E484,Q493,G496,Q498,Y505
C2485	G339,S375,K417,N440,G446,T478
C2486	G339,S375,K417,N440,G446,T478,G496,Q498,Y505
C2487	G339,S375,K417,N440,N501
C2488	G339,S375,K417,N440,S477,E484,Q493,Q498,N501,Y505
C2489	G339,S375,K417,N440,S477,T478
C2490	G339,S375,K417,N440,S477,T478,E484,N501
C2491	G339,S375,K417,N440,S477,T478,E484,Q493,Q498,N501,Y505

C2492	G339,S375,K417,N440,S477,T478,E484,Q493,Q498,Y505
C2493	G339,S375,K417,N440,S477,T478,E484,Q498,N501,Y505
C2494	G339,S375,K417,N440,S477,T478,E484,Q498,Y505
C2495	G339,S375,K417,N440,S477,T478,N501
C2496	G339,S375,K417,N440,S477,T478,Q498,N501,Y505
C2497	G339,S375,K417,N440,T478,E484,N501
C2498	G339,S375,K417,N440,T478,E484,Q493,Q498,N501,Y505
C2499	G339,S375,K417,N440,T478,E484,Q498,N501,Y505
C2500	G339,S375,K417,N440,T478,E484,Q498,Y505
C2501	G339,S375,K417,N440,T478,N501
C2502	G339,S375,K417,N440,T478,Q498,N501,Y505
C2503	G339,S375,K417,N440,T478,Q498,Y505
C2504	G339,S375,K417,S477,E484,Q493,G496,Q498,N501,Y505
C2505	G339,S375,K417,S477,T478
C2506	G339,S375,K417,S477,T478,E484,N501
C2507	G339,S375,K417,S477,T478,E484,Q493,Q498,N501,Y505
C2508	G339,S375,K417,S477,T478,E484,Q498,N501,Y505
C2509	G339,S375,K417,S477,T478,E484,Q498,Y505
C2510	G339,S375,K417,S477,T478,Q498,N501,Y505
C2511	G339,S375,K417,T478
C2512	G339,S375,K417,T478,E484,Q498,N501,Y505
C2513	G339,S375,K417,T478,E484,Q498,Y505
C2514	G339,S375,K417,T478,G496,Q498,Y505
C2515	G339,S375,N440,E484,N501
C2516	G339,S375,N440,G446,S477,E484,Q493,G496,Q498,N501
C2517	G339,S375,N440,G446,S477,E484,Q493,G496,Q498,N501,Y505
C2518	G339,S375,N440,G446,S477,T478
C2519	G339,S375,N440,G446,S477,T478,E484,Q493,G496,Q498
C2520	G339,S375,N440,G446,S477,T478,E484,Q493,G496,Q498,Y505
C2521	G339,S375,N440,G446,T478,E484,Q493,G496,Q498,Y505
C2522	G339,S375,N440,G446,T478,G496,Q498
C2523	G339,S375,N440,G446,T478,G496,Q498,Y505
C2524	G339,S375,N440,S477,E484,Q493,G496,Q498,N501,Y505
C2525	G339,S375,N440,S477,E484,Q493,Q498,N501,Y505
C2526	G339,S375,N440,S477,T478
C2527	G339,S375,N440,S477,T478,E484,N501
C2528	G339,S375,N440,S477,T478,E484,Q493,G496,Q498,Y505
C2529	G339,S375,N440,S477,T478,E484,Q493,Q498,N501
C2530	G339,S375,N440,S477,T478,E484,Q493,Q498,N501,Y505
C2531	G339,S375,N440,S477,T478,E484,Q493,Q498,Y505
C2532	G339,S375,N440,S477,T478,E484,Q498
C2533	G339,S375,N440,S477,T478,E484,Q498,N501
C2534	G339,S375,N440,S477,T478,E484,Q498,N501,Y505

C2535	G339,S375,N440,S477,T478,E484,Q498,Y505
C2536	G339,S375,N440,S477,T478,Q498,N501
C2537	G339,S375,N440,S477,T478,Q498,N501,Y505
C2538	G339,S375,N440,T478,E484,Q498,N501
C2539	G339,S375,N440,T478,E484,Q498,N501,Y505
C2540	G339,S375,N440,T478,G496,Q498,Y505
C2541	G339,S375,N440,T478,Q498,Y505
C2542	G339,S375,N501
C2543	G339,S375,Q493,G496,Q498,N501,Y505
C2544	G339,S375,Q493,Q498,N501,Y505
C2545	G339,S375,Q493,Q498,Y505
C2546	G339,S375,Q498
C2547	G339,S375,Q498,N501
C2548	G339,S375,Q498,N501,Y505
C2549	G339,S375,Q498,Y505
C2550	G339,S375,S477,E484,Q493,G496,Q498,N501,Y505
C2551	G339,S375,S477,E484,Q493,G496,Q498,Y505
C2552	G339,S375,S477,E484,Q493,Q498,N501,Y505
C2553	G339,S375,S477,E484,Q493,Q498,Y505
C2554	G339,S375,S477,E484,Q498,N501,Y505
C2555	G339,S375,S477,E484,Q498,Y505
C2556	G339,S375,S477,T478
C2557	G339,S375,S477,T478,E484,F486,Q498,N501,Y505
C2558	G339,S375,S477,T478,E484,F486,Q498,Y505
C2559	G339,S375,S477,T478,E484,N501
C2560	G339,S375,S477,T478,E484,Q493,G496,Q498,Y505
C2561	G339,S375,S477,T478,E484,Q493,Q498,N501,Y505
C2562	G339,S375,S477,T478,E484,Q493,Q498,Y505
C2563	G339,S375,S477,T478,E484,Q498
C2564	G339,S375,S477,T478,E484,Q498,N501,Y505
C2565	G339,S375,S477,T478,E484,Q498,Y505
C2566	G339,S375,S477,T478,N501
C2567	G339,S375,S477,T478,Q493,G496,Q498,N501
C2568	G339,S375,S477,T478,Q493,G496,Q498,N501,Y505
C2569	G339,S375,S477,T478,Q493,Q498,N501,Y505
C2570	G339,S375,S477,T478,Q498,N501,Y505
C2571	G339,S375,T376,D405,E484,N501
C2572	G339,S375,T376,D405,K417,N440,E484,N501
C2573	G339,S375,T376,D405,K417,N440,S477,T478,E484,N501
C2574	G339,S375,T376,D405,K417,N440,S477,T478,E484,Q493,Q498,N501,Y505
C2575	G339,S375,T376,D405,K417,N440,S477,T478,E484,Q498,N501,Y505
C2576	G339,S375,T376,D405,K417,N440,S477,T478,E484,Q498,Y505
C2577	G339,S375,T376,D405,K417,N440,S477,T478,Q498,N501,Y505

C2578	G339,S375,T376,D405,K417,N440,T478,E484,Q498,N501,Y505
C2579	G339,S375,T376,D405,K417,S477,T478,E484,Q498,Y505
C2580	G339,S375,T376,D405,N440,E484,N501
C2581	G339,S375,T376,D405,N440,S477,T478,E484,N501
C2582	G339,S375,T376,D405,N440,S477,T478,E484,Q493,Q498,N501,Y505
C2583	G339,S375,T376,D405,N440,S477,T478,E484,Q498,N501,Y505
C2584	G339,S375,T376,D405,N440,S477,T478,E484,Q498,Y505
C2585	G339,S375,T376,D405,N440,S477,T478,Q498,N501,Y505
C2586	G339,S375,T376,D405,N440,T478,E484,Q498,N501,Y505
C2587	G339,S375,T376,D405,R408,K417,N440,E484,N501
C2588	G339,S375,T376,D405,R408,K417,N440,S477,T478,E484,N501
C2589	G339,S375,T376,D405,R408,K417,N440,S477,T478,E484,Q493,Q498,N501,Y505
C2590	G339,S375,T376,D405,R408,K417,N440,S477,T478,E484,Q498,N501,Y505
C2591	G339,S375,T376,D405,R408,K417,N440,S477,T478,E484,Q498,Y505
C2592	G339,S375,T376,D405,R408,K417,N440,S477,T478,Q498,N501,Y505
C2593	G339,S375,T376,D405,R408,K417,N440,T478,E484,Q498,N501,Y505
C2594	G339,S375,T376,D405,R408,K417,S477,T478,E484,Q498,Y505
C2595	G339,S375,T376,D405,R408,N440,E484,N501
C2596	G339,S375,T376,D405,R408,N440,S477,T478,E484,N501
C2597	G339,S375,T376,D405,R408,N440,S477,T478,E484,Q493,Q498,N501,Y505
C2598	G339,S375,T376,D405,R408,N440,S477,T478,E484,Q498,N501,Y505
C2599	G339,S375,T376,D405,R408,N440,S477,T478,E484,Q498,Y505
C2600	G339,S375,T376,D405,R408,N440,S477,T478,Q498,N501,Y505
C2601	G339,S375,T376,D405,R408,N440,T478,E484,Q498,N501,Y505
C2602	G339,S375,T376,D405,R408,S477,T478,E484,Q498,Y505
C2603	G339,S375,T376,D405,S477,T478,E484,F486,Q498,N501,Y505
C2604	G339,S375,T376,D405,S477,T478,E484,F486,Q498,Y505
C2605	G339,S375,T376,D405,S477,T478,E484,N501
C2606	G339,S375,T376,D405,S477,T478,E484,Q493,Q498,N501,Y505
C2607	G339,S375,T376,D405,S477,T478,E484,Q498,N501,Y505
C2608	G339,S375,T376,D405,S477,T478,E484,Q498,Y505
C2609	G339,S375,T376,D405,S477,T478,Q498,N501,Y505
C2610	G339,S375,T376,D405,T478,E484,F486,Q498,N501,Y505
C2611	G339,S375,T376,D405,T478,E484,Q498,N501,Y505
C2612	G339,S375,T376,E484,F486,Q498,N501,Y505
C2613	G339,S375,T376,E484,F486,Q498,Y505
C2614	G339,S375,T376,E484,N501
C2615	G339,S375,T376,E484,Q493,Q498,N501,Y505
C2616	G339,S375,T376,E484,Q498,N501,Y505
C2617	G339,S375,T376,E484,Q498,Y505
C2618	G339,S375,T376,L452,E484,N501
C2619	G339,S375,T376,L452,S477,T478,E484,F486,Q498,Y505
C2620	G339,S375,T376,L452,T478,E484,F486,Q498,N501,Y505

C2621	G339,S375,T376,N501
C2622	G339,S375,T376,Q493,Q498,N501,Y505
C2623	G339,S375,T376,Q498
C2624	G339,S375,T376,Q498,N501
C2625	G339,S375,T376,Q498,N501,Y505
C2626	G339,S375,T376,Q498,Y505
C2627	G339,S375,T376,S477,T478,E484,F486,Q498,N501,Y505
C2628	G339,S375,T376,S477,T478,E484,F486,Q498,Y505
C2629	G339,S375,T376,S477,T478,E484,N501
C2630	G339,S375,T376,S477,T478,E484,Q493,Q498,N501,Y505
C2631	G339,S375,T376,S477,T478,E484,Q498,N501,Y505
C2632	G339,S375,T376,S477,T478,E484,Q498,Y505
C2633	G339,S375,T376,S477,T478,Q498,N501,Y505
C2634	G339,S375,T376,T478,E484,F486,Q498,N501,Y505
C2635	G339,S375,T376,T478,E484,N501
C2636	G339,S375,T376,T478,E484,Q493,Q498,N501,Y505
C2637	G339,S375,T376,T478,E484,Q498,N501,Y505
C2638	G339,S375,T376,T478,E484,Q498,Y505
C2639	G339,S375,T376,T478,Q498,N501,Y505
C2640	G339,S375,T478
C2641	G339,S375,T478,E484,F486,Q498,N501,Y505
C2642	G339,S375,T478,E484,Q493,Q498,Y505
C2643	G339,S375,T478,E484,Q498,N501,Y505
C2644	G339,S375,T478,G496,Q498,Y505
C2645	G339,S375,T478,Q493,G496,Q498,N501,Y505
C2646	G339,S375,T478,Q493,Q498,N501,Y505
C2647	G339,S375,T478,Q498,Y505
C2648	G339,S477
C2649	G339,S477,E484
C2650	G339,S477,E484,G496,Q498,N501,Y505
C2651	G339,S477,E484,G496,Q498,Y505
C2652	G339,S477,E484,G496,Y505
C2653	G339,S477,E484,N501
C2654	G339,S477,E484,N501,Y505
C2655	G339,S477,E484,Q493
C2656	G339,S477,E484,Q493,G496,Q498
C2657	G339,S477,E484,Q493,G496,Q498,N501
C2658	G339,S477,E484,Q493,G496,Q498,N501,Y505
C2659	G339,S477,E484,Q493,G496,Q498,Y505
C2660	G339,S477,E484,Q493,Q498
C2661	G339,S477,E484,Q493,Q498,N501
C2662	G339,S477,E484,Q493,Q498,N501,Y505
C2663	G339,S477,E484,Q493,Q498,Y505

C2664	G339,S477,E484,Q498
C2665	G339,S477,E484,Q498,N501
C2666	G339,S477,E484,Q498,N501,Y505
C2667	G339,S477,E484,Q498,Y505
C2668	G339,S477,E484,Y505
C2669	G339,S477,G496,N501,Y505
C2670	G339,S477,G496,Y505
C2671	G339,S477,N501
C2672	G339,S477,Q493,G496,Q498,N501,Y505
C2673	G339,S477,Q493,G496,Q498,Y505
C2674	G339,S477,Q493,Q498,N501,Y505
C2675	G339,S477,Q498,N501,Y505
C2676	G339,S477,Q498,Y505
C2677	G339,S477,T478
C2678	G339,S477,T478,E484
C2679	G339,S477,T478,E484,F486,Q498
C2680	G339,S477,T478,E484,F486,Q498,N501
C2681	G339,S477,T478,E484,F486,Q498,N501,Y505
C2682	G339,S477,T478,E484,F486,Q498,Y505
C2683	G339,S477,T478,E484,G496,Q498,Y505
C2684	G339,S477,T478,E484,N501
C2685	G339,S477,T478,E484,N501,Y505
C2686	G339,S477,T478,E484,Q493
C2687	G339,S477,T478,E484,Q493,G496,Q498
C2688	G339,S477,T478,E484,Q493,G496,Q498,Y505
C2689	G339,S477,T478,E484,Q493,N501,Y505
C2690	G339,S477,T478,E484,Q493,Q498
C2691	G339,S477,T478,E484,Q493,Q498,N501
C2692	G339,S477,T478,E484,Q493,Q498,Y505
C2693	G339,S477,T478,E484,Q493,Y505
C2694	G339,S477,T478,E484,Q498
C2695	G339,S477,T478,E484,Q498,N501
C2696	G339,S477,T478,E484,Q498,N501,Y505
C2697	G339,S477,T478,E484,Q498,Y505
C2698	G339,S477,T478,E484,Y505
C2699	G339,S477,T478,G496,N501,Y505
C2700	G339,S477,T478,G496,Q498,N501,Y505
C2701	G339,S477,T478,G496,Y505
C2702	G339,S477,T478,N501
C2703	G339,S477,T478,N501,Y505
C2704	G339,S477,T478,Q493
C2705	G339,S477,T478,Q493,G496,N501,Y505
C2706	G339,S477,T478,Q493,G496,Q498

C2707	G339,S477,T478,Q493,G496,Q498,N501
C2708	G339,S477,T478,Q493,G496,Q498,N501,Y505
C2709	G339,S477,T478,Q493,Q498
C2710	G339,S477,T478,Q493,Q498,N501
C2711	G339,S477,T478,Q493,Q498,N501,Y505
C2712	G339,S477,T478,Q498
C2713	G339,S477,T478,Q498,N501
C2714	G339,S477,T478,Q498,N501,Y505
C2715	G339,S477,T478,Y505
C2716	G339,S477,Y505
C2717	G339,T376,D405,E484,N501
C2718	G339,T376,D405,L452,E484,N501
C2719	G339,T376,D405,L452,S477,T478,E484,F486,Q498,Y505
C2720	G339,T376,D405,L452,T478,E484,F486,Q498,N501,Y505
C2721	G339,T376,D405,S477,T478,E484,F486,Q498,N501,Y505
C2722	G339,T376,D405,S477,T478,E484,F486,Q498,Y505
C2723	G339,T376,D405,S477,T478,E484,N501
C2724	G339,T376,D405,S477,T478,E484,Q498,N501,Y505
C2725	G339,T376,D405,S477,T478,E484,Q498,Y505
C2726	G339,T376,D405,S477,T478,Q498,N501,Y505
C2727	G339,T376,D405,T478,E484,F486,Q498,N501,Y505
C2728	G339,T376,E484,F486,Q498,N501,Y505
C2729	G339,T376,E484,F486,Q498,Y505
C2730	G339,T376,E484,N501
C2731	G339,T376,E484,Q498,N501,Y505
C2732	G339,T376,E484,Q498,Y505
C2733	G339,T376,Q498,N501,Y505
C2734	G339,T376,S477,T478,E484,F486,Q498,N501,Y505
C2735	G339,T376,S477,T478,E484,F486,Q498,Y505
C2736	G339,T376,S477,T478,E484,N501
C2737	G339,T376,S477,T478,E484,Q493,Q498,N501,Y505
C2738	G339,T376,S477,T478,E484,Q498,N501,Y505
C2739	G339,T376,S477,T478,E484,Q498,Y505
C2740	G339,T376,S477,T478,Q498,N501,Y505
C2741	G339,T376,T478,E484,F486,Q498,N501,Y505
C2742	G339,T376,T478,E484,N501
C2743	G339,T376,T478,E484,Q493,Q498,N501,Y505
C2744	G339,T376,T478,E484,Q498,N501,Y505
C2745	G339,T376,T478,E484,Q498,Y505
C2746	G339,T376,T478,Q498,N501,Y505
C2747	G339,T478
C2748	G339,T478,E484
C2749	G339,T478,E484,F486,Q498,N501

C2750	G339,T478,E484,F486,Q498,N501,Y505
C2751	G339,T478,E484,F486,Q498,Y505
C2752	G339,T478,E484,G496,Q498,Y505
C2753	G339,T478,E484,G496,Y505
C2754	G339,T478,E484,N501
C2755	G339,T478,E484,N501,Y505
C2756	G339,T478,E484,Q493
C2757	G339,T478,E484,Q493,G496
C2758	G339,T478,E484,Q493,G496,Q498
C2759	G339,T478,E484,Q493,G496,Q498,Y505
C2760	G339,T478,E484,Q493,G496,Y505
C2761	G339,T478,E484,Q493,Q498
C2762	G339,T478,E484,Q493,Q498,N501
C2763	G339,T478,E484,Q493,Q498,N501,Y505
C2764	G339,T478,E484,Q493,Q498,Y505
C2765	G339,T478,E484,Q493,Y505
C2766	G339,T478,E484,Q498
C2767	G339,T478,E484,Q498,N501
C2768	G339,T478,E484,Q498,N501,Y505
C2769	G339,T478,E484,Q498,Y505
C2770	G339,T478,E484,Y505
C2771	G339,T478,G496
C2772	G339,T478,G496,Q498
C2773	G339,T478,G496,Q498,N501,Y505
C2774	G339,T478,G496,Q498,Y505
C2775	G339,T478,G496,Y505
C2776	G339,T478,N501
C2777	G339,T478,N501,Y505
C2778	G339,T478,Q493
C2779	G339,T478,Q493,G496
C2780	G339,T478,Q493,G496,Q498
C2781	G339,T478,Q493,G496,Q498,N501
C2782	G339,T478,Q493,G496,Q498,N501,Y505
C2783	G339,T478,Q493,G496,Q498,Y505
C2784	G339,T478,Q493,G496,Y505
C2785	G339,T478,Q493,Q498,N501
C2786	G339,T478,Q493,Q498,N501,Y505
C2787	G339,T478,Q493,Q498,Y505
C2788	G339,T478,Q498
C2789	G339,T478,Q498,N501
C2790	G339,T478,Q498,N501,Y505
C2791	G339,T478,Q498,Y505
C2792	G339,T478,Y505

C2793	G339,Y505
C2794	G446
C2795	G446,E484
C2796	G446,E484,G496,N501
C2797	G446,E484,Q493
C2798	G446,E484,Q493,G496,Q498,N501,Y505
C2799	G446,G496,N501
C2800	G446,G496,Q498,Y505
C2801	G446,N501
C2802	G446,Q493
C2803	G446,Q493,G496,Q498,N501,Y505
C2804	G446,Q493,G496,Q498,Y505
C2805	G446,Q493,Q498,N501,Y505
C2806	G446,S477
C2807	G446,S477,E484,Q493,G496,Q498,N501
C2808	G446,S477,E484,Q493,G496,Q498,N501,Y505
C2809	G446,S477,G496,N501
C2810	G446,S477,Q493
C2811	G446,S477,Q493,G496,Q498,N501,Y505
C2812	G446,S477,Q493,G496,Q498,Y505
C2813	G446,S477,Q493,Q498,N501,Y505
C2814	G446,S477,T478
C2815	G446,S477,T478,E484
C2816	G446,S477,T478,E484,G496,N501
C2817	G446,S477,T478,E484,Q493,G496,Q498
C2818	G446,S477,T478,E484,Q493,G496,Q498,Y505
C2819	G446,S477,T478,E484,Q493,Q498,N501
C2820	G446,S477,T478,E484,Q493,Q498,N501,Y505
C2821	G446,S477,T478,G496,N501
C2822	G446,S477,T478,Q493
C2823	G446,T478
C2824	G446,T478,E484
C2825	G446,T478,E484,G496
C2826	G446,T478,E484,G496,N501
C2827	G446,T478,E484,Q493
C2828	G446,T478,E484,Q493,G496,Q498,Y505
C2829	G446,T478,E484,Q493,Q498,N501,Y505
C2830	G446,T478,G496,N501
C2831	G446,T478,G496,Q498
C2832	G446,T478,G496,Q498,Y505
C2833	G446,T478,Q493
C2834	G496
C2835	G496,N501

C2836	G496,N501,Y505
C2837	G496,Q498
C2838	G496,Q498,N501
C2839	G496,Q498,N501,Y505
C2840	G496,Q498,Y505
C2841	G496,Y505
C2842	K417
C2843	K417,E484
C2844	K417,E484,G496,N501
C2845	K417,E484,N501
C2846	K417,E484,Q493,Q498,N501,Y505
C2847	K417,E484,Q498
C2848	K417,E484,Q498,N501,Y505
C2849	K417,E484,Q498,Y505
C2850	K417,G446,S477,T478,E484,G496,N501
C2851	K417,L452
C2852	K417,L452,E484
C2853	K417,L452,T478
C2854	K417,L452,T478,E484
C2855	K417,N440
C2856	K417,N440,E484
C2857	K417,N440,E484,N501
C2858	K417,N440,E484,Q493
C2859	K417,N440,E484,Q493,Q498,N501,Y505
C2860	K417,N440,E484,Q498,N501
C2861	K417,N440,E484,Q498,N501,Y505
C2862	K417,N440,G446,S477,E484
C2863	K417,N440,G446,S477,E484,Q493,G496,Q498
C2864	K417,N440,G446,S477,E484,Q493,G496,Q498,N501,Y505
C2865	K417,N440,G446,S477,T478
C2866	K417,N440,G446,S477,T478,E484
C2867	K417,N440,G446,S477,T478,E484,G496
C2868	K417,N440,G446,S477,T478,E484,G496,N501
C2869	K417,N440,G446,S477,T478,E484,Q493,G496,Q498
C2870	K417,N440,G446,S477,T478,E484,Q493,G496,Q498,Y505
C2871	K417,N440,G446,S477,T478,E484,Q493,Q498
C2872	K417,N440,G446,S477,T478,E484,Q493,Q498,N501,Y505
C2873	K417,N440,G446,T478
C2874	K417,N440,G446,T478,G496,Q498
C2875	K417,N440,G446,T478,G496,Q498,Y505
C2876	K417,N440,N501,Y505
C2877	K417,N440,Q493
C2878	K417,N440,Q493,Q498,N501,Y505

C2879	K417,N440,S477
C2880	K417,N440,S477,E484
C2881	K417,N440,S477,E484,N501
C2882	K417,N440,S477,E484,Q493,Q498,N501,Y505
C2883	K417,N440,S477,E484,Q498,N501,Y505
C2884	K417,N440,S477,N501
C2885	K417,N440,S477,Q493,Q498,N501,Y505
C2886	K417,N440,S477,T478
C2887	K417,N440,S477,T478,E484
C2888	K417,N440,S477,T478,E484,N501
C2889	K417,N440,S477,T478,E484,N501,Y505
C2890	K417,N440,S477,T478,E484,Q493
C2891	K417,N440,S477,T478,E484,Q493,N501,Y505
C2892	K417,N440,S477,T478,E484,Q493,Q498
C2893	K417,N440,S477,T478,E484,Q493,Q498,N501
C2894	K417,N440,S477,T478,E484,Q493,Q498,Y505
C2895	K417,N440,S477,T478,E484,Q493,Y505
C2896	K417,N440,S477,T478,E484,Q498
C2897	K417,N440,S477,T478,E484,Q498,N501
C2898	K417,N440,S477,T478,E484,Q498,N501,Y505
C2899	K417,N440,S477,T478,E484,Q498,Y505
C2900	K417,N440,S477,T478,N501
C2901	K417,N440,S477,T478,N501,Y505
C2902	K417,N440,S477,T478,Q493,Q498,N501,Y505
C2903	K417,N440,S477,T478,Q498
C2904	K417,N440,S477,T478,Q498,N501,Y505
C2905	K417,N440,S477,T478,Y505
C2906	K417,N440,T478
C2907	K417,N440,T478,E484
C2908	K417,N440,T478,E484,N501
C2909	K417,N440,T478,E484,Q493,Q498,N501,Y505
C2910	K417,N440,T478,E484,Q498
C2911	K417,N440,T478,E484,Q498,N501,Y505
C2912	K417,N440,T478,E484,Q498,Y505
C2913	K417,N440,T478,Q498
C2914	K417,N440,T478,Q498,N501
C2915	K417,N440,T478,Q498,Y505
C2916	K417,N501
C2917	K417,Q493
C2918	K417,Q493,N501
C2919	K417,Q493,Q498,N501,Y505
C2920	K417,Q498,N501,Y505
C2921	K417,Q498,Y505

C2922	K417,S477,E484,N501
C2923	K417,S477,E484,Q493,Q498,N501,Y505
C2924	K417,S477,T478
C2925	K417,S477,T478,E484
C2926	K417,S477,T478,E484,N501
C2927	K417,S477,T478,E484,Q493
C2928	K417,S477,T478,E484,Q493,Q498,N501
C2929	K417,S477,T478,E484,Q493,Q498,N501,Y505
C2930	K417,S477,T478,E484,Q498
C2931	K417,S477,T478,E484,Q498,N501,Y505
C2932	K417,S477,T478,E484,Q498,Y505
C2933	K417,T478
C2934	K417,T478,E484
C2935	K417,T478,E484,Q498
C2936	K417,T478,E484,Q498,N501,Y505
C2937	K417,T478,E484,Y505
C2938	K417,T478,Q493,Q498,N501,Y505
C2939	K417,T478,Q498
C2940	K417,T478,Q498,Y505
C2941	K417,T478,Y505
C2942	K417,Y505
C2943	L452
C2944	L452,E484
C2945	L452,E484,N501
C2946	L452,N501
C2947	L452,S477
C2948	L452,S477,E484
C2949	L452,S477,E484,N501
C2950	L452,S477,T478
C2951	L452,S477,T478,E484
C2952	L452,T478,E484
C2953	L452,T478,E484,Q498,N501,Y505
C2954	L452,T478,E484,Q498,Y505
C2955	L452,T478,F486
C2956	L452,T478,N501
C2957	L452,T478,Q498
C2958	L452,T478,Q498,N501
C2959	N440
C2960	N440,E484
C2961	N440,E484,G496,N501
C2962	N440,E484,N501
C2963	N440,E484,Q493,N501,Y505
C2964	N440,E484,Q493,Q498,N501,Y505

C2965	N440,E484,Q498,N501,Y505
C2966	N440,G446
C2967	N440,G446,E484,G496,N501
C2968	N440,G446,E484,Q493,G496,Q498,N501,Y505
C2969	N440,G446,N501
C2970	N440,G446,S477,E484
C2971	N440,G446,S477,E484,G496,N501
C2972	N440,G446,S477,E484,Q493,G496,Q498,N501,Y505
C2973	N440,G446,S477,T478
C2974	N440,G446,S477,T478,E484
C2975	N440,G446,S477,T478,E484,G496
C2976	N440,G446,S477,T478,E484,G496,N501
C2977	N440,G446,S477,T478,E484,N501
C2978	N440,G446,S477,T478,E484,Q493,G496,Q498
C2979	N440,G446,S477,T478,E484,Q493,G496,Q498,Y505
C2980	N440,G446,S477,T478,E484,Q493,Q498,N501,Y505
C2981	N440,G446,T478
C2982	N440,G446,T478,E484,G496,N501
C2983	N440,G446,T478,E484,Q493,G496,Q498,Y505
C2984	N440,G446,T478,E484,Q493,Q498,N501,Y505
C2985	N440,G446,T478,G496,N501
C2986	N440,G446,T478,G496,Q498,Y505
C2987	N440,G496,N501
C2988	N440,L452
C2989	N440,L452,E484,N501
C2990	N440,L452,S477,E484,N501
C2991	N440,L452,S477,T478,E484,Q498,Y505
C2992	N440,L452,T478
C2993	N440,L452,T478,E484,Q498,N501,Y505
C2994	N440,N501
C2995	N440,N501,Y505
C2996	N440,Q493
C2997	N440,Q493,N501
C2998	N440,Q493,Q498
C2999	N440,Q493,Q498,N501
C3000	N440,Q493,Q498,N501,Y505
C3001	N440,S477
C3002	N440,S477,E484
C3003	N440,S477,E484,N501
C3004	N440,S477,E484,Q493,G496,Q498,N501,Y505
C3005	N440,S477,E484,Q493,Q498,N501,Y505
C3006	N440,S477,E484,Q498,N501,Y505
C3007	N440,S477,T478

C3008	N440,S477,T478,E484
C3009	N440,S477,T478,E484,F486,Q498,N501,Y505
C3010	N440,S477,T478,E484,F486,Q498,Y505
C3011	N440,S477,T478,E484,G496,N501
C3012	N440,S477,T478,E484,N501
C3013	N440,S477,T478,E484,Q493
C3014	N440,S477,T478,E484,Q493,G496,Q498,Y505
C3015	N440,S477,T478,E484,Q493,N501,Y505
C3016	N440,S477,T478,E484,Q493,Q498
C3017	N440,S477,T478,E484,Q493,Q498,N501
C3018	N440,S477,T478,E484,Q493,Q498,N501,Y505
C3019	N440,S477,T478,E484,Q493,Q498,Y505
C3020	N440,S477,T478,E484,Q498,N501,Y505
C3021	N440,S477,T478,E484,Q498,Y505
C3022	N440,S477,T478,Q493,Q498,N501,Y505
C3023	N440,S477,T478,Q498,N501,Y505
C3024	N440,T478
C3025	N440,T478,E484
C3026	N440,T478,E484,F486,Q498,N501,Y505
C3027	N440,T478,E484,G496,N501
C3028	N440,T478,E484,N501
C3029	N440,T478,E484,Q493,Q498,N501
C3030	N440,T478,E484,Q493,Q498,N501,Y505
C3031	N440,T478,E484,Q498,N501,Y505
C3032	N440,T478,E484,Q498,Y505
C3033	N440,T478,G496,Q498,Y505
C3034	N440,T478,Q493,Q498,N501,Y505
C3035	N440,T478,Q498,N501,Y505
C3036	N440,T478,Q498,Y505
C3037	N501,Y505
C3038	Q493
C3039	Q493,G496
C3040	Q493,G496,N501
C3041	Q493,G496,N501,Y505
C3042	Q493,G496,Q498
C3043	Q493,G496,Q498,N501
C3044	Q493,G496,Q498,N501,Y505
C3045	Q493,G496,Q498,Y505
C3046	Q493,G496,Y505
C3047	Q493,N501
C3048	Q493,N501,Y505
C3049	Q493,Q498
C3050	Q493,Q498,N501

C3051	Q493,Q498,N501,Y505
C3052	Q493,Q498,Y505
C3053	Q493,Y505
C3054	Q498
C3055	Q498,N501
C3056	Q498,N501,Y505
C3057	Q498,Y505
C3058	R346
C3059	R346,E484
C3060	R346,G446,S477,Q493,G496,Q498,Y505
C3061	R346,G496,N501
C3062	R346,N501
C3063	R346,S371,S373,S375,G496,N501
C3064	R346,S371,S373,S375,K417,N440,G446,S477,T478,E484,Q493,G496,Q498,Y505
C3065	R346,S371,S373,S375,S477,E484,Q493,G496,Q498,Y505
C3066	R346,S477
C3067	R346,S477,E484,Q493,G496,Q498,Y505
C3068	R346,S477,Q493,G496,Q498,Y505
C3069	R346,S477,T478,E484,Q493,G496,Q498,Y505
C3070	R346,T478
C3071	R346,T478,E484
C3072	R408
C3073	R408,E484
C3074	R408,K417,L452,S477,E484,N501
C3075	R408,K417,N440,E484,N501
C3076	R408,K417,N440,S477,E484,N501
C3077	R408,K417,N440,S477,T478,E484,N501
C3078	R408,K417,N440,S477,T478,E484,Q493,Q498,N501,Y505
C3079	R408,K417,N440,S477,T478,E484,Q498,N501,Y505
C3080	R408,K417,N440,S477,T478,E484,Q498,Y505
C3081	R408,K417,N440,S477,T478,Q498,N501,Y505
C3082	R408,K417,N440,T478,E484,Q498,N501,Y505
C3083	R408,K417,S477,E484,N501
C3084	R408,K417,S477,T478,E484,Q498,Y505
C3085	R408,L452
C3086	R408,L452,T478
C3087	R408,N501
C3088	R408,S477
C3089	R408,S477,E484,N501
C3090	R408,T478
C3091	S371
C3092	S371,D405,E484
C3093	S371,D405,E484,N501

C3094	S371,D405,K417,E484,N501
C3095	S371,D405,K417,L452,S477,E484,N501
C3096	S371,D405,K417,N440,E484,N501
C3097	S371,D405,K417,N440,L452,E484,N501
C3098	S371,D405,K417,N440,L452,S477,E484,N501
C3099	S371,D405,K417,N440,L452,S477,N501
C3100	S371,D405,K417,N440,S477,E484,N501
C3101	S371,D405,K417,N501
C3102	S371,D405,K417,S477,E484,N501
C3103	S371,D405,L452,E484,N501
C3104	S371,D405,L452,S477,E484,N501
C3105	S371,D405,N440,E484,N501
C3106	S371,D405,N440,L452,E484,N501
C3107	S371,D405,N440,L452,S477,E484
C3108	S371,D405,N440,L452,S477,E484,N501
C3109	S371,D405,N440,S477,E484,N501
C3110	S371,D405,N501
C3111	S371,D405,R408,K417,L452
C3112	S371,D405,R408,K417,L452,S477,E484
C3113	S371,D405,R408,K417,L452,S477,E484,N501
C3114	S371,D405,R408,K417,N440
C3115	S371,D405,R408,K417,N440,E484,N501
C3116	S371,D405,R408,K417,N440,L452
C3117	S371,D405,R408,K417,N440,L452,E484,N501
C3118	S371,D405,R408,K417,N440,L452,N501
C3119	S371,D405,R408,K417,N440,L452,S477,E484
C3120	S371,D405,R408,K417,N440,L452,S477,N501
C3121	S371,D405,R408,K417,N440,N501
C3122	S371,D405,R408,K417,N440,S477
C3123	S371,D405,R408,K417,N440,S477,E484
C3124	S371,D405,R408,K417,N440,S477,E484,N501
C3125	S371,D405,R408,K417,N440,S477,N501
C3126	S371,D405,R408,K417,N501
C3127	S371,D405,R408,K417,S477,E484
C3128	S371,D405,R408,K417,S477,E484,N501
C3129	S371,D405,R408,L452,S477,E484,N501
C3130	S371,D405,R408,N440,L452,S477,E484,N501
C3131	S371,D405,R408,N440,S477,E484
C3132	S371,D405,R408,N440,S477,E484,N501
C3133	S371,D405,R408,S477,E484,N501
C3134	S371,D405,S477,E484,N501
C3135	S371,D405,S477,N501
C3136	S371,E484

C3137	S371,E484,N501
C3138	S371,E484,Q498,N501,Y505
C3139	S371,E484,Q498,Y505
C3140	S371,K417
C3141	S371,K417,E484
C3142	S371,K417,E484,N501
C3143	S371,K417,L452,N501
C3144	S371,K417,L452,S477,E484,N501
C3145	S371,K417,N440
C3146	S371,K417,N440,E484
C3147	S371,K417,N440,E484,N501
C3148	S371,K417,N440,L452,S477,E484,N501
C3149	S371,K417,N440,N501
C3150	S371,K417,N440,S477
C3151	S371,K417,N440,S477,E484
C3152	S371,K417,N440,S477,E484,N501
C3153	S371,K417,N440,S477,N501
C3154	S371,K417,N440,T478
C3155	S371,K417,N501
C3156	S371,K417,S477
C3157	S371,K417,S477,E484
C3158	S371,K417,S477,E484,N501
C3159	S371,K417,S477,T478
C3160	S371,K417,T478
C3161	S371,K417,T478,E484
C3162	S371,L452
C3163	S371,L452,E484
C3164	S371,L452,E484,N501
C3165	S371,L452,N501
C3166	S371,L452,S477,E484
C3167	S371,L452,S477,E484,N501
C3168	S371,L452,S477,N501
C3169	S371,L452,T478
C3170	S371,N440
C3171	S371,N440,E484,N501
C3172	S371,N440,L452,S477,E484,N501
C3173	S371,N440,N501
C3174	S371,N440,S477
C3175	S371,N440,S477,E484
C3176	S371,N440,S477,E484,N501
C3177	S371,N440,S477,N501
C3178	S371,N440,S477,T478
C3179	S371,N440,T478

C3180	S371,N501
C3181	S371,R408,K417,L452,S477,E484,N501
C3182	S371,R408,K417,N440,L452,S477,E484,N501
C3183	S371,R408,K417,N440,S477,E484,N501
C3184	S371,R408,K417,S477,E484,N501
C3185	S371,R408,L452,S477,E484,N501
C3186	S371,R408,N440,S477,E484,N501
C3187	S371,R408,S477,E484,N501
C3188	S371,S373
C3189	S371,S373,E484
C3190	S371,S373,E484,N501
C3191	S371,S373,K417
C3192	S371,S373,K417,G446,G496,N501
C3193	S371,S373,K417,L452,N501
C3194	S371,S373,K417,L452,T478,Q498,N501,Y505
C3195	S371,S373,K417,L452,T478,Q498,Y505
C3196	S371,S373,K417,N440,G446,S477,T478,E484,G496,N501
C3197	S371,S373,K417,N440,G446,T478,N501
C3198	S371,S373,K417,N440,S477,T478,E484,N501
C3199	S371,S373,K417,Q493,Q498,N501,Y505
C3200	S371,S373,K417,T478
C3201	S371,S373,K417,T478,N501
C3202	S371,S373,K417,T478,Q493,Q498,N501,Y505
C3203	S371,S373,K417,T478,Q493,Q498,Y505
C3204	S371,S373,K417,T478,Q498,N501,Y505
C3205	S371,S373,K417,T478,Q498,Y505
C3206	S371,S373,N440,G446,S477,T478,E484,G496,N501
C3207	S371,S373,N440,S477,T478,E484,N501
C3208	S371,S373,N501
C3209	S371,S373,Q493,Q498,Y505
C3210	S371,S373,S375
C3211	S371,S373,S375,E484
C3212	S371,S373,S375,E484,G496
C3213	S371,S373,S375,E484,G496,N501
C3214	S371,S373,S375,E484,N501
C3215	S371,S373,S375,E484,Q493,Q498,N501,Y505
C3216	S371,S373,S375,E484,Q493,Q498,Y505
C3217	S371,S373,S375,E484,Q498,N501,Y505
C3218	S371,S373,S375,E484,Q498,Y505
C3219	S371,S373,S375,G446
C3220	S371,S373,S375,G446,E484,G496
C3221	S371,S373,S375,G446,E484,G496,N501
C3222	S371,S373,S375,G446,G496,N501

C3223	S371,S373,S375,G446,S477,T478
C3224	S371,S373,S375,G446,S477,T478,E484,G496
C3225	S371,S373,S375,G446,S477,T478,E484,G496,N501
C3226	S371,S373,S375,G446,S477,T478,E484,N501
C3227	S371,S373,S375,G496
C3228	S371,S373,S375,G496,N501
C3229	S371,S373,S375,K417
C3230	S371,S373,S375,K417,E484,N501
C3231	S371,S373,S375,K417,G446,E484
C3232	S371,S373,S375,K417,G446,E484,G496,N501
C3233	S371,S373,S375,K417,G446,S477,T478
C3234	S371,S373,S375,K417,G446,S477,T478,E484,G496,N501
C3235	S371,S373,S375,K417,G446,S477,T478,E484,N501
C3236	S371,S373,S375,K417,N440
C3237	S371,S373,S375,K417,N440,E484,N501
C3238	S371,S373,S375,K417,N440,G446
C3239	S371,S373,S375,K417,N440,G446,E484
C3240	S371,S373,S375,K417,N440,G446,E484,G496,N501
C3241	S371,S373,S375,K417,N440,G446,G496
C3242	S371,S373,S375,K417,N440,G446,G496,N501
C3243	S371,S373,S375,K417,N440,G446,N501
C3244	S371,S373,S375,K417,N440,G446,S477
C3245	S371,S373,S375,K417,N440,G446,S477,E484,G496,N501
C3246	S371,S373,S375,K417,N440,G446,S477,E484,Q493,G496,Q498,N501,Y505
C3247	S371,S373,S375,K417,N440,G446,S477,G496,N501
C3248	S371,S373,S375,K417,N440,G446,S477,T478
C3249	S371,S373,S375,K417,N440,G446,S477,T478,E484
C3250	S371,S373,S375,K417,N440,G446,S477,T478,E484,G496
C3251	S371,S373,S375,K417,N440,G446,S477,T478,E484,N501
C3252	S371,S373,S375,K417,N440,G446,S477,T478,E484,Q493,G496,Q498,Y505
C3253	S371,S373,S375,K417,N440,G446,S477,T478,G496,N501
C3254	S371,S373,S375,K417,N440,G446,S477,T478,N501
C3255	S371,S373,S375,K417,N440,G446,T478,E484,G496,N501
C3256	S371,S373,S375,K417,N440,G446,T478,G496
C3257	S371,S373,S375,K417,N440,G446,T478,G496,Q498,Y505
C3258	S371,S373,S375,K417,N440,N501
C3259	S371,S373,S375,K417,N440,S477,E484,N501
C3260	S371,S373,S375,K417,N440,S477,E484,Q493,Q498,N501,Y505
C3261	S371,S373,S375,K417,N440,S477,E484,Q498,N501,Y505
C3262	S371,S373,S375,K417,N440,S477,T478
C3263	S371,S373,S375,K417,N440,S477,T478,E484
C3264	S371,S373,S375,K417,N440,S477,T478,E484,G496,N501
C3265	S371,S373,S375,K417,N440,S477,T478,E484,N501

C3266	S371,S373,S375,K417,N440,S477,T478,E484,Q493,Q498,N501,Y505
C3267	S371,S373,S375,K417,N440,S477,T478,E484,Q493,Q498,Y505
C3268	S371,S373,S375,K417,N440,S477,T478,E484,Q498,N501,Y505
C3269	S371,S373,S375,K417,N440,S477,T478,E484,Q498,Y505
C3270	S371,S373,S375,K417,N440,S477,T478,N501
C3271	S371,S373,S375,K417,N440,S477,T478,Q498,N501,Y505
C3272	S371,S373,S375,K417,N440,T478,E484,N501
C3273	S371,S373,S375,K417,N440,T478,E484,Q498,N501,Y505
C3274	S371,S373,S375,K417,N440,T478,Q498,Y505
C3275	S371,S373,S375,K417,N501
C3276	S371,S373,S375,K417,S477,E484,G496,N501
C3277	S371,S373,S375,K417,S477,T478
C3278	S371,S373,S375,K417,S477,T478,E484
C3279	S371,S373,S375,K417,S477,T478,E484,G496,N501
C3280	S371,S373,S375,K417,S477,T478,E484,N501
C3281	S371,S373,S375,K417,S477,T478,E484,Q498,Y505
C3282	S371,S373,S375,K417,T478
C3283	S371,S373,S375,K417,T478,E484,N501
C3284	S371,S373,S375,L452
C3285	S371,S373,S375,L452,T478
C3286	S371,S373,S375,N440
C3287	S371,S373,S375,N440,E484,N501
C3288	S371,S373,S375,N440,G446
C3289	S371,S373,S375,N440,G446,E484,G496,N501
C3290	S371,S373,S375,N440,G446,G496
C3291	S371,S373,S375,N440,G446,G496,N501
C3292	S371,S373,S375,N440,G446,N501
C3293	S371,S373,S375,N440,G446,S477
C3294	S371,S373,S375,N440,G446,S477,T478
C3295	S371,S373,S375,N440,G446,S477,T478,E484
C3296	S371,S373,S375,N440,G446,S477,T478,E484,G496
C3297	S371,S373,S375,N440,G446,S477,T478,E484,G496,N501
C3298	S371,S373,S375,N440,G446,S477,T478,E484,Q493,G496,Q498,Y505
C3299	S371,S373,S375,N440,G446,S477,T478,G496,N501
C3300	S371,S373,S375,N440,G496,N501
C3301	S371,S373,S375,N440,S477,T478,E484
C3302	S371,S373,S375,N440,S477,T478,E484,G496
C3303	S371,S373,S375,N440,S477,T478,E484,G496,N501
C3304	S371,S373,S375,N440,S477,T478,E484,N501
C3305	S371,S373,S375,N440,S477,T478,E484,Q493,Q498,Y505
C3306	S371,S373,S375,N440,S477,T478,E484,Q498,Y505
C3307	S371,S373,S375,N440,T478,E484,N501
C3308	S371,S373,S375,N501

C3309	S371,S373,S375,Q493,Q498,N501,Y505
C3310	S371,S373,S375,Q493,Q498,Y505
C3311	S371,S373,S375,Q498,N501,Y505
C3312	S371,S373,S375,Q498,Y505
C3313	S371,S373,S375,S477,E484,G496,N501
C3314	S371,S373,S375,S477,E484,N501
C3315	S371,S373,S375,S477,E484,Q493,G496,Q498,N501,Y505
C3316	S371,S373,S375,S477,E484,Q493,G496,Q498,Y505
C3317	S371,S373,S375,S477,E484,Q493,Q498,N501,Y505
C3318	S371,S373,S375,S477,E484,Q493,Q498,Y505
C3319	S371,S373,S375,S477,E484,Q498,N501,Y505
C3320	S371,S373,S375,S477,E484,Q498,Y505
C3321	S371,S373,S375,S477,T478
C3322	S371,S373,S375,S477,T478,E484
C3323	S371,S373,S375,S477,T478,E484,G496
C3324	S371,S373,S375,S477,T478,E484,G496,N501
C3325	S371,S373,S375,S477,T478,E484,N501
C3326	S371,S373,S375,S477,T478,E484,Q493,G496,Q498,Y505
C3327	S371,S373,S375,S477,T478,E484,Q493,Q498,N501,Y505
C3328	S371,S373,S375,S477,T478,E484,Q493,Q498,Y505
C3329	S371,S373,S375,S477,T478,E484,Q498,N501,Y505
C3330	S371,S373,S375,S477,T478,E484,Q498,Y505
C3331	S371,S373,S375,S477,T478,G496,N501
C3332	S371,S373,S375,S477,T478,N501
C3333	S371,S373,S375,S477,T478,Q493,G496,Q498,N501,Y505
C3334	S371,S373,S375,S477,T478,Q493,Q498,N501,Y505
C3335	S371,S373,S375,S477,T478,Q498,N501,Y505
C3336	S371,S373,S375,T376,D405,R408,K417
C3337	S371,S373,S375,T376,D405,R408,K417,N440
C3338	S371,S373,S375,T376,D405,R408,K417,N440,E484,N501
C3339	S371,S373,S375,T376,D405,R408,K417,N440,L452
C3340	S371,S373,S375,T376,D405,R408,K417,N440,L452,E484,N501
C3341	S371,S373,S375,T376,D405,R408,K417,N440,L452,S477,T478,E484,F486,Q498,Y505
C3342	S371,S373,S375,T376,D405,R408,K417,N440,L452,T478,E484,F486,Q498,N501,Y505
C3343	S371,S373,S375,T376,D405,R408,K417,N440,S477,T478,E484,F486,Q498,N501,Y505
C3344	S371,S373,S375,T376,D405,R408,K417,N440,S477,T478,E484,N501
C3345	S371,S373,S375,T376,D405,R408,K417,N440,S477,T478,E484,Q493,Q498,N501,Y505
C3346	S371,S373,S375,T376,D405,R408,K417,N440,S477,T478,E484,Q498,N501,Y505
C3347	S371,S373,S375,T376,D405,R408,K417,N440,S477,T478,E484,Q498,Y505
C3348	S371,S373,S375,T376,D405,R408,K417,N440,S477,T478,Q498,N501,Y505
C3349	S371,S373,S375,T376,D405,R408,K417,N440,T478,E484,Q498,N501,Y505
C3350	S371,S373,S375,T376,D405,R408,K417,S477,T478,E484,Q498,Y505
C3351	S371,S373,S375,T376,E484,F486,Q498,N501,Y505

C3352	S371,S373,S375,T376,E484,F486,Q498,Y505
C3353	S371,S373,S375,T376,E484,N501
C3354	S371,S373,S375,T376,E484,Q493,Q498,N501,Y505
C3355	S371,S373,S375,T376,E484,Q498,N501,Y505
C3356	S371,S373,S375,T376,E484,Q498,Y505
C3357	S371,S373,S375,T376,F486,Q498,N501,Y505
C3358	S371,S373,S375,T376,F486,Q498,Y505
C3359	S371,S373,S375,T376,K417,N440,E484,N501
C3360	S371,S373,S375,T376,K417,N440,S477,T478,E484,N501
C3361	S371,S373,S375,T376,K417,N440,S477,T478,E484,Q493,Q498,N501,Y505
C3362	S371,S373,S375,T376,K417,N440,S477,T478,E484,Q498,N501,Y505
C3363	S371,S373,S375,T376,K417,N440,S477,T478,E484,Q498,Y505
C3364	S371,S373,S375,T376,K417,N440,S477,T478,Q498,N501,Y505
C3365	S371,S373,S375,T376,K417,N440,T478,E484,Q498,N501,Y505
C3366	S371,S373,S375,T376,K417,S477,T478,E484,Q498,Y505
C3367	S371,S373,S375,T376,N501
C3368	S371,S373,S375,T376,Q498,N501,Y505
C3369	S371,S373,S375,T376,Q498,Y505
C3370	S371,S373,S375,T376,S477,T478,E484,F486,Q498,N501,Y505
C3371	S371,S373,S375,T376,S477,T478,E484,F486,Q498,Y505
C3372	S371,S373,S375,T376,S477,T478,E484,N501
C3373	S371,S373,S375,T376,S477,T478,E484,Q493,Q498,N501,Y505
C3374	S371,S373,S375,T376,S477,T478,E484,Q498,N501,Y505
C3375	S371,S373,S375,T376,S477,T478,E484,Q498,Y505
C3376	S371,S373,S375,T376,S477,T478,Q498,N501,Y505
C3377	S371,S373,S375,T376,T478,E484,F486,Q498,N501,Y505
C3378	S371,S373,S375,T376,T478,E484,F486,Q498,Y505
C3379	S371,S373,S375,T376,T478,E484,N501
C3380	S371,S373,S375,T376,T478,E484,Q498,N501,Y505
C3381	S371,S373,S375,T376,T478,E484,Q498,Y505
C3382	S371,S373,S375,T376,T478,Q498,N501,Y505
C3383	S371,S373,S375,T478
C3384	S371,S373,S375,T478,E484
C3385	S371,S373,S375,T478,E484,G496
C3386	S371,S373,S375,T478,E484,G496,N501
C3387	S371,S373,S375,T478,E484,N501
C3388	S371,S373,S375,T478,E484,Q498,N501,Y505
C3389	S371,S373,S375,T478,E484,Q498,Y505
C3390	S371,S373,S375,T478,G496
C3391	S371,S373,S375,T478,G496,N501
C3392	S371,S373,S375,T478,G496,Q498,Y505
C3393	S371,S373,S375,T478,Q498,N501,Y505
C3394	S371,S373,S375,T478,Q498,Y505

C3395	S371,S373,S477,E484,Q498,N501,Y505
C3396	S371,S373,S477,E484,Q498,Y505
C3397	S371,S373,S477,T478
C3398	S371,S373,S477,T478,E484,F486,Q498,N501,Y505
C3399	S371,S373,S477,T478,E484,F486,Q498,Y505
C3400	S371,S373,S477,T478,E484,G496,N501
C3401	S371,S373,S477,T478,E484,N501
C3402	S371,S373,S477,T478,E484,Q498,N501,Y505
C3403	S371,S373,S477,T478,E484,Q498,Y505
C3404	S371,S373,S477,T478,Q498,N501,Y505
C3405	S371,S373,T376,E484,N501
C3406	S371,S373,T376,S477,T478,E484,F486,Q498,N501,Y505
C3407	S371,S373,T376,S477,T478,E484,F486,Q498,Y505
C3408	S371,S373,T376,S477,T478,E484,N501
C3409	S371,S373,T376,S477,T478,E484,Q498,N501,Y505
C3410	S371,S373,T376,S477,T478,E484,Q498,Y505
C3411	S371,S373,T376,S477,T478,Q498,N501,Y505
C3412	S371,S373,T376,T478,E484,F486,Q498,N501,Y505
C3413	S371,S373,T478,E484,F486,Q498,N501,Y505
C3414	S371,S373,T478,E484,N501
C3415	S371,S373,T478,Q493,Q498,N501,Y505
C3416	S371,S373,T478,Q493,Q498,Y505
C3417	S371,S373,T478,Q498,Y505
C3418	S371,S375,K417,N440,G446,S477,T478,E484,G496,N501
C3419	S371,S375,K417,N440,G446,T478,E484,G496,N501
C3420	S371,S375,K417,N440,S477,T478,E484,N501
C3421	S371,S375,S477,T478,E484,N501
C3422	S371,S477
C3423	S371,S477,E484
C3424	S371,S477,E484,N501
C3425	S371,S477,E484,Q493,Q498,N501,Y505
C3426	S371,S477,E484,Q493,Q498,Y505
C3427	S371,S477,N501
C3428	S371,S477,T478
C3429	S371,S477,T478,E484
C3430	S371,S477,T478,E484,N501
C3431	S371,S477,T478,E484,Q493,Q498,N501,Y505
C3432	S371,S477,T478,E484,Q493,Q498,Y505
C3433	S371,S477,T478,E484,Q498,N501,Y505
C3434	S371,S477,T478,E484,Q498,Y505
C3435	S371,S477,T478,Q493,Q498,N501,Y505
C3436	S371,S477,T478,Q498,N501,Y505
C3437	S371,T478

C3438	S371,T478,E484
C3439	S371,T478,E484,F486,Q498,N501,Y505
C3440	S371,T478,E484,F486,Q498,Y505
C3441	S371,T478,E484,N501
C3442	S371,T478,E484,Q498,N501,Y505
C3443	S371,T478,E484,Q498,Y505
C3444	S371,T478,Q498,N501,Y505
C3445	S371,T478,Q498,Y505
C3446	S373
C3447	S373,E484
C3448	S373,E484,F486,Q498,N501,Y505
C3449	S373,E484,F486,Q498,Y505
C3450	S373,E484,G496,N501
C3451	S373,E484,N501
C3452	S373,E484,Q498,N501,Y505
C3453	S373,E484,Q498,Y505
C3454	S373,K417,E484
C3455	S373,K417,N440,S477,T478,E484,N501
C3456	S373,K417,S477,T478,E484,N501
C3457	S373,L452
C3458	S373,L452,T478
C3459	S373,N440,G446,S477,T478,E484,G496,N501
C3460	S373,N440,G446,S477,T478,E484,N501
C3461	S373,N440,S477,T478,E484,N501
C3462	S373,N501
C3463	S373,Q493,Q498,N501,Y505
C3464	S373,Q493,Q498,Y505
C3465	S373,Q498,N501,Y505
C3466	S373,Q498,Y505
C3467	S373,S375
C3468	S373,S375,E484
C3469	S373,S375,E484,G496,N501
C3470	S373,S375,E484,N501
C3471	S373,S375,E484,Q493,Q498,N501,Y505
C3472	S373,S375,E484,Q493,Q498,Y505
C3473	S373,S375,E484,Q498
C3474	S373,S375,E484,Q498,N501
C3475	S373,S375,E484,Q498,N501,Y505
C3476	S373,S375,E484,Q498,Y505
C3477	S373,S375,G446,S477,T478,E484,G496,N501
C3478	S373,S375,G496,N501
C3479	S373,S375,K417,N440,G446,S477,T478,E484,G496,N501
C3480	S373,S375,K417,N440,S477,E484,Q493,Q498,N501,Y505

C3481	S373,S375,K417,N440,S477,T478
C3482	S373,S375,K417,N440,S477,T478,E484,N501
C3483	S373,S375,K417,N440,S477,T478,E484,Q493,Q498,Y505
C3484	S373,S375,K417,N440,T478,Q498,Y505
C3485	S373,S375,K417,S477,E484,Q493,Q498,N501,Y505
C3486	S373,S375,K417,S477,E484,Q498,N501,Y505
C3487	S373,S375,K417,S477,T478
C3488	S373,S375,K417,S477,T478,E484,N501
C3489	S373,S375,K417,S477,T478,E484,Q493,Q498,Y505
C3490	S373,S375,K417,S477,T478,E484,Q498,Y505
C3491	S373,S375,K417,T478,Q498,Y505
C3492	S373,S375,N440,G446,S477,T478,E484,G496,N501
C3493	S373,S375,N440,G446,T478,E484,G496,N501
C3494	S373,S375,N440,S477,E484,Q493,Q498,N501,Y505
C3495	S373,S375,N440,S477,E484,Q498,N501,Y505
C3496	S373,S375,N440,S477,T478
C3497	S373,S375,N440,S477,T478,E484,N501
C3498	S373,S375,N440,S477,T478,E484,Q493,Q498,Y505
C3499	S373,S375,N440,S477,T478,E484,Q498,Y505
C3500	S373,S375,N440,T478,Q498,Y505
C3501	S373,S375,N501
C3502	S373,S375,Q493,Q498,N501,Y505
C3503	S373,S375,Q498
C3504	S373,S375,Q498,N501
C3505	S373,S375,Q498,N501,Y505
C3506	S373,S375,Q498,Y505
C3507	S373,S375,S477,E484,Q493,Q498,N501,Y505
C3508	S373,S375,S477,E484,Q493,Q498,Y505
C3509	S373,S375,S477,E484,Q498,N501,Y505
C3510	S373,S375,S477,E484,Q498,Y505
C3511	S373,S375,S477,T478
C3512	S373,S375,S477,T478,E484,G496,N501
C3513	S373,S375,S477,T478,E484,N501
C3514	S373,S375,S477,T478,E484,Q493,Q498,Y505
C3515	S373,S375,S477,T478,E484,Q498,Y505
C3516	S373,S375,S477,T478,G496,N501
C3517	S373,S375,S477,T478,Q493,Q498,N501,Y505
C3518	S373,S375,S477,T478,Q498,N501,Y505
C3519	S373,S375,T376
C3520	S373,S375,T376,D405,E484,N501
C3521	S373,S375,T376,D405,K417,E484,N501
C3522	S373,S375,T376,D405,K417,S477,T478,E484,N501
C3523	S373,S375,T376,D405,K417,S477,T478,E484,Q493,Q498,N501,Y505

C3524	S373,S375,T376,D405,K417,S477,T478,E484,Q498,N501,Y505
C3525	S373,S375,T376,D405,K417,S477,T478,E484,Q498,Y505
C3526	S373,S375,T376,D405,K417,S477,T478,Q498,N501,Y505
C3527	S373,S375,T376,D405,K417,T478,E484,Q498,N501,Y505
C3528	S373,S375,T376,D405,R408,K417,E484,N501
C3529	S373,S375,T376,D405,R408,K417,N440,L452,E484,N501
C3530	S373,S375,T376,D405,R408,K417,N440,L452,S477,T478,E484,Q498,Y505
C3531	S373,S375,T376,D405,R408,K417,N440,L452,T478,E484,Q498,N501,Y505
C3532	S373,S375,T376,D405,R408,K417,N440,S477,T478,E484,N501
C3533	S373,S375,T376,D405,R408,K417,N440,S477,T478,E484,Q493,Q498,N501,Y505
C3534	S373,S375,T376,D405,R408,K417,N440,S477,T478,E484,Q498,N501,Y505
C3535	S373,S375,T376,D405,R408,K417,N440,S477,T478,Q498,N501,Y505
C3536	S373,S375,T376,D405,R408,K417,S477,T478,E484,N501
C3537	S373,S375,T376,D405,R408,K417,S477,T478,E484,Q493,Q498,N501,Y505
C3538	S373,S375,T376,D405,R408,K417,S477,T478,E484,Q498,N501,Y505
C3539	S373,S375,T376,D405,R408,K417,S477,T478,E484,Q498,Y505
C3540	S373,S375,T376,D405,R408,K417,S477,T478,Q498,N501,Y505
C3541	S373,S375,T376,D405,R408,K417,T478,E484,Q498,N501,Y505
C3542	S373,S375,T376,D405,S477,T478,E484,F486,Q498,N501,Y505
C3543	S373,S375,T376,D405,S477,T478,E484,F486,Q498,Y505
C3544	S373,S375,T376,D405,S477,T478,E484,N501
C3545	S373,S375,T376,D405,S477,T478,E484,Q498,N501,Y505
C3546	S373,S375,T376,D405,S477,T478,E484,Q498,Y505
C3547	S373,S375,T376,D405,S477,T478,Q498,N501,Y505
C3548	S373,S375,T376,D405,T478,E484,F486,Q498,N501,Y505
C3549	S373,S375,T376,E484,F486,Q498
C3550	S373,S375,T376,E484,F486,Q498,N501
C3551	S373,S375,T376,E484,F486,Q498,N501,Y505
C3552	S373,S375,T376,E484,F486,Q498,Y505
C3553	S373,S375,T376,E484,N501
C3554	S373,S375,T376,E484,Q493,Q498,N501,Y505
C3555	S373,S375,T376,E484,Q498
C3556	S373,S375,T376,E484,Q498,N501
C3557	S373,S375,T376,E484,Q498,N501,Y505
C3558	S373,S375,T376,E484,Q498,Y505
C3559	S373,S375,T376,K417,E484,N501
C3560	S373,S375,T376,K417,S477,T478,E484,F486,Q498,N501,Y505
C3561	S373,S375,T376,K417,S477,T478,E484,F486,Q498,Y505
C3562	S373,S375,T376,K417,S477,T478,E484,N501
C3563	S373,S375,T376,K417,S477,T478,E484,Q498,N501,Y505
C3564	S373,S375,T376,K417,S477,T478,E484,Q498,Y505
C3565	S373,S375,T376,K417,S477,T478,Q498,N501,Y505
C3566	S373,S375,T376,K417,T478,E484,F486,Q498,N501,Y505

C3567	S373,S375,T376,N440,E484,N501
C3568	S373,S375,T376,N440,S477,T478,E484,F486,Q498,N501,Y505
C3569	S373,S375,T376,N440,S477,T478,E484,F486,Q498,Y505
C3570	S373,S375,T376,N440,S477,T478,E484,N501
C3571	S373,S375,T376,N440,S477,T478,E484,Q493,Q498,N501,Y505
C3572	S373,S375,T376,N440,S477,T478,E484,Q498,N501,Y505
C3573	S373,S375,T376,N440,S477,T478,E484,Q498,Y505
C3574	S373,S375,T376,N440,S477,T478,Q498,N501,Y505
C3575	S373,S375,T376,N440,T478,E484,F486,Q498,N501,Y505
C3576	S373,S375,T376,N440,T478,E484,Q498,N501,Y505
C3577	S373,S375,T376,N501
C3578	S373,S375,T376,Q498,N501
C3579	S373,S375,T376,Q498,N501,Y505
C3580	S373,S375,T376,Q498,Y505
C3581	S373,S375,T376,R408,E484,N501
C3582	S373,S375,T376,R408,S477,T478,E484,N501
C3583	S373,S375,T376,R408,S477,T478,E484,Q493,Q498,N501,Y505
C3584	S373,S375,T376,R408,S477,T478,E484,Q498,N501,Y505
C3585	S373,S375,T376,R408,S477,T478,E484,Q498,Y505
C3586	S373,S375,T376,R408,S477,T478,Q498,N501,Y505
C3587	S373,S375,T376,R408,T478,E484,Q498,N501,Y505
C3588	S373,S375,T376,S477,T478,E484,F486,Q498,N501,Y505
C3589	S373,S375,T376,S477,T478,E484,F486,Q498,Y505
C3590	S373,S375,T376,S477,T478,E484,N501
C3591	S373,S375,T376,S477,T478,E484,Q493,Q498,N501,Y505
C3592	S373,S375,T376,S477,T478,E484,Q498,N501,Y505
C3593	S373,S375,T376,S477,T478,E484,Q498,Y505
C3594	S373,S375,T376,S477,T478,Q498,N501,Y505
C3595	S373,S375,T376,T478,E484,F486,Q498,N501,Y505
C3596	S373,S375,T376,T478,E484,F486,Q498,Y505
C3597	S373,S375,T376,T478,E484,N501
C3598	S373,S375,T376,T478,E484,Q493,Q498,N501,Y505
C3599	S373,S375,T376,T478,E484,Q498,N501,Y505
C3600	S373,S375,T376,T478,E484,Q498,Y505
C3601	S373,S375,T376,T478,Q498,N501,Y505
C3602	S373,S375,T478
C3603	S373,S375,T478,E484,N501
C3604	S373,S375,T478,E484,Q493,Q498,Y505
C3605	S373,S375,T478,E484,Q498,Y505
C3606	S373,S375,T478,Q493,Q498,N501,Y505
C3607	S373,S375,T478,Q498,N501,Y505
C3608	S373,S375,T478,Q498,Y505
C3609	S373,S477,E484,Q493,Q498,N501,Y505

C3610	S373,S477,E484,Q493,Q498,Y505
C3611	S373,S477,E484,Q498,N501,Y505
C3612	S373,S477,E484,Q498,Y505
C3613	S373,S477,T478
C3614	S373,S477,T478,E484,F486,Q498,N501,Y505
C3615	S373,S477,T478,E484,F486,Q498,Y505
C3616	S373,S477,T478,E484,G496,N501
C3617	S373,S477,T478,E484,N501
C3618	S373,S477,T478,E484,Q493,Q498,N501,Y505
C3619	S373,S477,T478,E484,Q493,Q498,Y505
C3620	S373,S477,T478,E484,Q498,N501,Y505
C3621	S373,S477,T478,E484,Q498,Y505
C3622	S373,S477,T478,Q493,Q498,N501,Y505
C3623	S373,S477,T478,Q498,N501,Y505
C3624	S373,T478
C3625	S373,T478,E484
C3626	S373,T478,E484,F486,Q498,N501,Y505
C3627	S373,T478,E484,N501
C3628	S373,T478,E484,Q498,N501,Y505
C3629	S373,T478,Q498,Y505
C3630	S375
C3631	S375,E484
C3632	S375,E484,N501
C3633	S375,E484,Q498,N501,Y505
C3634	S375,E484,Q498,Y505
C3635	S375,G446,E484,G496,N501
C3636	S375,G446,S477,T478,E484,G496,N501
C3637	S375,G496,N501
C3638	S375,K417,N440,G446,S477,T478,E484,G496,N501
C3639	S375,K417,N440,G446,S477,T478,N501
C3640	S375,K417,N440,G446,T478,E484,G496,N501
C3641	S375,K417,N440,S477,T478,E484,N501
C3642	S375,K417,S477,T478,E484,G496,N501
C3643	S375,L452
C3644	S375,L452,T478
C3645	S375,N440,G446,S477,T478,E484,G496,N501
C3646	S375,N440,S477,T478,E484,G496,N501
C3647	S375,N440,S477,T478,E484,N501
C3648	S375,N501
C3649	S375,Q498,N501,Y505
C3650	S375,Q498,Y505
C3651	S375,S477,E484,Q493,G496,Q498,N501,Y505
C3652	S375,S477,E484,Q493,G496,Q498,Y505

C3653	S375,S477,E484,Q493,Q498,N501,Y505
C3654	S375,S477,E484,Q493,Q498,Y505
C3655	S375,S477,E484,Q498,N501,Y505
C3656	S375,S477,E484,Q498,Y505
C3657	S375,S477,T478
C3658	S375,S477,T478,E484,G496,N501
C3659	S375,S477,T478,E484,N501
C3660	S375,S477,T478,E484,Q493,G496,Q498,Y505
C3661	S375,S477,T478,E484,Q493,Q498,N501,Y505
C3662	S375,S477,T478,E484,Q493,Q498,Y505
C3663	S375,S477,T478,E484,Q498,N501,Y505
C3664	S375,S477,T478,E484,Q498,Y505
C3665	S375,S477,T478,Q493,G496,Q498,N501,Y505
C3666	S375,S477,T478,Q493,Q498,N501,Y505
C3667	S375,S477,T478,Q498,N501,Y505
C3668	S375,T376
C3669	S375,T376,E484
C3670	S375,T376,E484,F486,Q498,N501,Y505
C3671	S375,T376,E484,F486,Q498,Y505
C3672	S375,T376,E484,N501
C3673	S375,T376,E484,Q498,N501,Y505
C3674	S375,T376,E484,Q498,Y505
C3675	S375,T376,N501
C3676	S375,T376,Q498,N501,Y505
C3677	S375,T376,Q498,Y505
C3678	S375,T376,S477,T478,E484,F486,Q498,N501,Y505
C3679	S375,T376,S477,T478,E484,F486,Q498,Y505
C3680	S375,T376,S477,T478,E484,N501
C3681	S375,T376,S477,T478,E484,Q493,Q498,N501,Y505
C3682	S375,T376,S477,T478,E484,Q498,N501,Y505
C3683	S375,T376,S477,T478,E484,Q498,Y505
C3684	S375,T376,S477,T478,Q498,N501,Y505
C3685	S375,T376,T478,E484,F486,Q498,N501,Y505
C3686	S375,T376,T478,E484,F486,Q498,Y505
C3687	S375,T376,T478,E484,N501
C3688	S375,T376,T478,E484,Q498,N501,Y505
C3689	S375,T376,T478,E484,Q498,Y505
C3690	S375,T376,T478,Q498,N501,Y505
C3691	S375,T478
C3692	S375,T478,E484,N501
C3693	S375,T478,E484,Q498,N501,Y505
C3694	S375,T478,E484,Q498,Y505
C3695	S375,T478,G496,Q498,Y505

C3696	S375,T478,Q498,N501,Y505
C3697	S375,T478,Q498,Y505
C3698	S477
C3699	S477,E484
C3700	S477,E484,F486,Q498,N501,Y505
C3701	S477,E484,F486,Q498,Y505
C3702	S477,E484,G496,N501
C3703	S477,E484,N501
C3704	S477,E484,N501,Y505
C3705	S477,E484,Q493,G496,Q498
C3706	S477,E484,Q493,G496,Q498,N501,Y505
C3707	S477,E484,Q493,G496,Q498,Y505
C3708	S477,E484,Q493,Q498,N501,Y505
C3709	S477,E484,Q493,Q498,Y505
C3710	S477,E484,Q498
C3711	S477,E484,Q498,N501
C3712	S477,E484,Q498,N501,Y505
C3713	S477,E484,Q498,Y505
C3714	S477,E484,Y505
C3715	S477,G496,N501
C3716	S477,N501
C3717	S477,Q493
C3718	S477,Q493,G496,Q498,N501,Y505
C3719	S477,Q493,G496,Q498,Y505
C3720	S477,Q493,Q498,N501,Y505
C3721	S477,Q493,Q498,Y505
C3722	S477,Q498,N501,Y505
C3723	S477,Q498,Y505
C3724	S477,T478
C3725	S477,T478,E484
C3726	S477,T478,E484,F486,Q498
C3727	S477,T478,E484,F486,Q498,N501
C3728	S477,T478,E484,F486,Q498,N501,Y505
C3729	S477,T478,E484,F486,Q498,Y505
C3730	S477,T478,E484,G496,N501
C3731	S477,T478,E484,G496,N501,Y505
C3732	S477,T478,E484,G496,Q498,N501,Y505
C3733	S477,T478,E484,N501
C3734	S477,T478,E484,N501,Y505
C3735	S477,T478,E484,Q493
C3736	S477,T478,E484,Q493,G496
C3737	S477,T478,E484,Q493,G496,N501,Y505
C3738	S477,T478,E484,Q493,G496,Q498

C3739	S477,T478,E484,Q493,G496,Q498,N501
C3740	S477,T478,E484,Q493,G496,Q498,Y505
C3741	S477,T478,E484,Q493,G496,Y505
C3742	S477,T478,E484,Q493,N501,Y505
C3743	S477,T478,E484,Q493,Q498
C3744	S477,T478,E484,Q493,Q498,N501
C3745	S477,T478,E484,Q493,Q498,N501,Y505
C3746	S477,T478,E484,Q493,Q498,Y505
C3747	S477,T478,E484,Q493,Y505
C3748	S477,T478,E484,Q498
C3749	S477,T478,E484,Q498,N501
C3750	S477,T478,E484,Q498,N501,Y505
C3751	S477,T478,E484,Q498,Y505
C3752	S477,T478,E484,Y505
C3753	S477,T478,G496,N501
C3754	S477,T478,G496,N501,Y505
C3755	S477,T478,G496,Q498,N501,Y505
C3756	S477,T478,N501
C3757	S477,T478,N501,Y505
C3758	S477,T478,Q493
C3759	S477,T478,Q493,G496,Q498
C3760	S477,T478,Q493,G496,Q498,N501
C3761	S477,T478,Q493,G496,Q498,N501,Y505
C3762	S477,T478,Q493,G496,Q498,Y505
C3763	S477,T478,Q493,Q498,N501,Y505
C3764	S477,T478,Q493,Q498,Y505
C3765	S477,T478,Q498
C3766	S477,T478,Q498,N501
C3767	S477,T478,Q498,N501,Y505
C3768	S477,T478,Q498,Y505
C3769	S477,T478,Y505
C3770	S477,Y505
C3771	T376
C3772	T376,E484,N501
C3773	T376,L452
C3774	T376,L452,T478
C3775	T376,N501
C3776	T376,S477,T478,E484,N501
C3777	T376,S477,T478,E484,Q493,Q498,N501,Y505
C3778	T376,S477,T478,E484,Q498,N501,Y505
C3779	T376,S477,T478,E484,Q498,Y505
C3780	T376,S477,T478,Q498,N501,Y505
C3781	T376,T478

C3782	T376,T478,E484,Q498,N501,Y505
C3783	T478,E484
C3784	T478,E484,F486,Q498
C3785	T478,E484,F486,Q498,N501
C3786	T478,E484,F486,Q498,N501,Y505
C3787	T478,E484,F486,Q498,Y505
C3788	T478,E484,G496
C3789	T478,E484,G496,N501
C3790	T478,E484,G496,Q498,N501,Y505
C3791	T478,E484,G496,Y505
C3792	T478,E484,N501
C3793	T478,E484,N501,Y505
C3794	T478,E484,Q493
C3795	T478,E484,Q493,G496
C3796	T478,E484,Q493,G496,Q498
C3797	T478,E484,Q493,G496,Q498,N501
C3798	T478,E484,Q493,G496,Q498,N501,Y505
C3799	T478,E484,Q493,G496,Q498,Y505
C3800	T478,E484,Q493,G496,Y505
C3801	T478,E484,Q493,N501
C3802	T478,E484,Q493,Q498
C3803	T478,E484,Q493,Q498,N501
C3804	T478,E484,Q493,Q498,N501,Y505
C3805	T478,E484,Q493,Q498,Y505
C3806	T478,E484,Q493,Y505
C3807	T478,E484,Q498
C3808	T478,E484,Q498,N501
C3809	T478,E484,Q498,N501,Y505
C3810	T478,E484,Q498,Y505
C3811	T478,E484,Y505
C3812	T478,F486
C3813	T478,G496
C3814	T478,G496,N501
C3815	T478,G496,Q498
C3816	T478,G496,Q498,Y505
C3817	T478,N501
C3818	T478,Q493
C3819	T478,Q493,G496,Q498
C3820	T478,Q493,G496,Q498,N501
C3821	T478,Q493,G496,Q498,N501,Y505
C3822	T478,Q493,N501
C3823	T478,Q493,Q498,N501
C3824	T478,Q493,Q498,N501,Y505

C3825	T478,Q493,Q498,Y505
C3826	T478,Q498
C3827	T478,Q498,N501
C3828	T478,Q498,N501,Y505
C3829	T478,Q498,Y505
C3830	T478,Y505
C3831	Y505
