# Supplementary material for: Efficient discovery of frequently co-occurring mutations in a sequence database with matrix factorization
Source: PLoS Comput Biol. 2025 Apr 24;21(4):e1012391. doi: 10.1371/journal.pcbi.1012391 (PMC12273922; doi:10.1371/journal.pcbi.1012391)
Supplement: Attachment — Submitted filename: Response to Reviewers.pdf [file pcbi.1012391.s002.pdf]

**Reviewer #1**

The manuscript addresses an important problem in computational biology and proposes a potentially useful solution that is broadly applicable across different domains within the field. The article itself appears to be well-written and organized. However, I have identified several key points that need to be addressed. I have outlined them below.

1. Algorithm: The use of non-negative matrix factorization (NMF) to address this problem seems appropriate. However, given that the NMF literature has grown by leaps and bounds within the past two decades, the rationale for using the original NMF algorithm based on the Frobenius norm for this application remains unclear.

Numerous improvements to this method have been around for years now, not only from an algorithmic perspective but also from a statistical perspective, i.e., for handling different data and noise structures. Arguably, the applications for which the proposed method is expected to be utilized would be better served by an approach that effectively deals with discrete multivariate data. Specifically, numerous NMF algorithms based on the Poisson model exist for handling large, sparse count data and these have been shown to be far more superior in identifying factors (rows of H or columns of W) than any other alternative by inherently accounting for the data structure. Methods also exist for explicitly imposing sparsity constraints on H or W, or both, as appropriate. Specific examples regarding Poisson-based NMF algorithms and their applications include, but not limited to, Lee & Seung (1999, 2001), Cichocki et al. (2006, 2008, 2009), Brunet et al. (2003), Chagoyen et al. (2006) and Devarajan et al. (2015, 2021). The book by Cichocki et al. (2009) is particularly relevant and a useful reference here. The HALS algorithm of Cichocki et al. could provide for more efficient and faster algorithms that can improve computational times; however, its implementation is likely limited to the Frobenius norm.

At the very least, what would be useful to the audience for this manuscript would be a comparison of the proposed method with a method based on the Poisson model. This would also help provide a balanced view of the proposed approach since it has only been compared to a brute force approach. The use of more recent methods would also permit the imposition of explicit sparsity constraints on the rows of H or the columns of W (or both), depending on the application. This would, in turn, facilitate the efficient identification of potentially useful factors in a more objective manner for further exploration (see comment #2 below).

1-Algo: "... the rationale for using the original NMF algorithm based on the Frobenius norm for this application remains unclear."

*Response*: Thank you for giving us the opportunity to clarify. The Frobenius norm is used here because our domains are unlikely to have any statistical noise. For the latter case Poisson/Gaussian distribution based KL-divergence is used. We have added an extensive literature survey on NMF literature now in the NMF sub-section under Materials and methods.

2. In lines 117-122, the authors describe an ad hoc approach for selecting factors from H where the mean of the entries of H is used to select the threshold which will eventually determine elements that are truncated to 0. The authors claim that this will suppress noise and maintain sparsity in the matrix. However, this approach seems rather arbitrary since the distribution of the elements of H will have an impact on the result. For example, what is the impact of this method when this distribution is skewed with few elements of very high magnitude versus a situation with a more evenly distributed H. There is a wealth of currently available methods to address this problem in a systematic manner. A more robust and agnostic approach would be to impose explicit sparsity constraints on the elements of H (see comment #1 above). This foolproof approach is versatile in that it can handle situations where the distribution of elements of H varies widely.

*Response:* Literature is ripe with methods for identifying the "best" rank/factor number. We have adopted one of them ("dissimilarity" metric or DSIM), to find a range of factors. This is because we observed that factorization with different pre-selected rank/factor numbers lead to different local minima. Many of these local minima are useful in producing meaningful factors (with their presence in the database in significant numbers, which is our target criterion). Hence, instead of selecting a single "best" factor, we have chosen a range of factors, providing a more comprehensive coverage of the search domain.

3. Factor selection: Numerous measures for selecting the number of components, r, in NMF exist in the literature. It should be pointed out that the concept behind the dissimilarity measure between factors described in this manuscript appears to have been outlined in Brunet et al. (2003). However, I am not aware of a quantitative measure based on the product HH'. In that regard, it would be prudent to consider the aforementioned paper and cite it as appropriate.

*Response:* You are absolutely correct in pointing out the importance of Brunet, and we have updated the paper to cite Brunet as well as any other sources we considered for r selection. They are covered in more detail in the question and response immediately below.

Have the authors considered any of the other existing measures in the literature for choosing the rank r for comparison purposes? Does the proposed measure always result in a better interpretation of the decomposition?

*Response*: Yes, we looked at the methods done by Brunet and Gaujoux who both plotted the cophenetic correlation coefficient against rank quantity (Brunet, 2004, Gaujoux, 2010). As well as Hutchins who plotted the residual sum of squares against rank quantity (Hutchins, 2008). All three of these methods shared an approach where they plotted a diagram of their y-axis metrics against a range of possible rank values,

and they looked for an “inflection point” where there was a noticeable decrease in the y-value. We experimented with this technique across multiple metrics until we settled on DSIM. References are available in the manuscript.

4. Further details regarding the implementation of the proposed methods and code utilized are not provided. Including them as Supplemental Information would strengthen the manuscript.

*Response:* We have elaborated on our methods in the paper. Additionally, we added a link to our GitHub repository to elucidate our implementation.

Minor comments:

1. The projected gradient method of Lin (2007) as well as the numerous subsequent NMF algorithms all stem from the original, seminal paper of Lee & Seung (2001) which is not cited here.

*Response:* Good point, the paper has been updated to discuss Lee and Seung’s contributions.

2. There have been recent developments in multi-layered NMF that helps identify hierarchical structures within the data. How would such an approach be relevant or useful to the application in this paper? Some comments on this aspect would help strengthen the paper and provide an avenue for further work on this topic.

*Response*: Hierarchical clustering has been shown to be an effective tool for identifying the heritability of a trait (Matta, 2015). The heritability of genotypes is applicable to immunology research because it is valuable to understand the spread of infectious or vaccine-resistant genotypes. Although the sheer complexity of genetics implies that extracting meaningful traits as explainable features can be a challenging problem. A common solution for a problem that is too complex to solve is to decompose the original problem into more manageable subproblems. Fortunately, NMF serves two purposes (1) to reduce the dimensionality of the data, that is, to reduce the complexity and (2) to extract meaningful data features. This means that instead of forcing NMF to shoulder both these responsibilities simultaneously, NMF can be layered so that earlier layers serve as a preprocessing tool to break up complex patterns into smaller problems for later layers. Experiments have already shown that stacked NMF architectures show an improvement in classification problems (Song, 2015).

Researchers have even created models that synthesize hierarchical NMF with deep learning neural techniques, called Deep NMF (DNMF) (Chen, 2022). This means that multilayered NMF architectures do not even need to be unidirectional; in fact, Le Roux et al integrated a feedback technique commonly used in deep learning neural networks called back-propagation into their DNMF model (LeRoux, 2015). However, their method could not support hierarchical capabilities. Though it opened the door for further innovation with the creation of Neural NMF which can support both, so it can

reap the benefits of clustering data containing hierarchical features and the error reduction provided by back-propagation (Haddock, 2023).

The literature constantly provides inspiration for future work; NMF and machine learning are truly exciting and ever-evolving fields of research because of the endless new directions for research. However, one should not write of the concise method presented due to a lack of layer or complexity. After all, Tanaka et al were successful in linking genes to corresponding phenotypes with their “systems-based genetic approach” that analyzed systems holistically as opposed to alternative two-step approaches that perform statistical association before any gene analysis (Tanaka, 2021). They credited their holistic analysis for catching signals that more layered methods can overlook (Tanaka, 2021). Demonstrating that the quality of the analysis is not directly correlated with the complexity of the method and that there is value in method diversification because some techniques can make discoveries overlooked by others. References are available in the manuscript.

## Reviewer #2

1. The authors adopted the modified Levenshtein distance to represent point mutations. How does this cost function prioritize biologically impactful mutations over neutral ones? Have you tested other scoring systems such as BLOSUM or PAM for comparison?

Criticism 1:The authors adopted the modified Levenshtein distance to represent point mutations. How does this cost function prioritize biologically impactful mutations over neutral ones

*Response*: Thank you for your careful review. The cost function is grounded in the principle that mutations occur at the DNA level and accumulate, potentially leading to amino acid changes which impact the protein. The cost function does not directly guarantee the identification of biologically impactful mutations automatically, as it does not bias for structure changes or make use of structural data or concepts such as. However, the fact that these discovered mutations and subsequent co-mutations take several DNA changes to reach, as well as the fact that a mutation occurs in large frequency in the dataset, increases the likelihood that we are picking up mutations relevant to functional properties such as binding affinity, immune evasion, or structural stability. Yes, it is possible for a relevant mutation to occur from a single position (i.e not in the wobble region), nevertheless, if such a mutation is prevalent (due to its’ biological relevance), one would expect it to be identified as a part of the CMP.

For example, if we consider the **[N501]** position in our dataset**,** the change from N to Y on average resulted in a cost score of 1 (a change reflected within ~500,000 sequences); despite this, due to the prevalence of this mutation in a large majority of the sequences, it still remained a relevant part of many CMPs. As this has been identified in the literature as one of the earliest key mutations, it demonstrates that our cost function does not disproportionately favor more complex mutations with a score of 3.

Criticism2: Have you tested other scoring systems such as BLOSUM or PAM?

*Response*: We did not use BLOSUM nor PAM because those metrics use negative numbers that our non-negative matrix factorization cannot handle.

2. Since the mutation matrix V is sparse, have you evaluated how sparsity impacts the performance of NMF? For example, could denser matrices with additional features such as weighted mutation scores improve the results?

*Response:* It is an interesting idea to see how dense matrices may be handled by the NMF method. However, our data set is fixed on SARS-COV-2 which was sparse and was our only target in this study and there was no scope to experiment with the input matrix. Rather, how to better utilize the sparsity (possibly as a constraint as the Reviewer-1 suggested) may be something that could be explored. In fact, we expect sparsity in our data since the majority of the residues in the protein are conserved, as we are comparing the same types of sequences (i.e SARS-COV-2 RBD to SARS-COV-2 RBD), with specific mutations in the evolution of the protein. Yet, this would be an interesting point to consider if the work was extended to compare sequences that have extensive mutations.

3. You selected the threshold $\Delta<0.1$ to filter redundant CMPs. Was this value optimized through cross-validation, or was it selected heuristically? Could a more adaptive threshold improve results? Please discuss this more.

*Response:* It was selected somewhat heuristically after many experimentations over grid search. Describing these experiments in the article may be overwhelming, and we believe it was not immediately important toward our result. In the spirit of reproducibility, we presented the cut-off value. The cutoff ensured that CMPs did not have too significant of an overlap between them but also balanced the removal of a CMP with the potential loss of information.

4. Table 1 shows the frequencies of CMPs, but there is no discussion of the thresholds used to consider a CMP biologically significant. Could low-frequency CMPs play a critical role in rare but important evolutionary events?

*Response:* The reviewer brings up an interesting point regarding the significance a low-frequency CMP could have in the course of evolutionary events; however, without direct experimentation and extended longitudinal data it may be difficult to separate meaningful CMPs from simple CMPs which arose randomly and did not elicit a significant response. We conducted an extensive literature survey for the biological significance of the CMPs and presented some of the findings in the Biological significance of CMPs subsection from Discussion. It is quite possible that a low-frequency co-mutation may be important, but if it were in the database it did not show its presence in significant numbers, and hence we could not detect it. By our definition, CMP does not cover such events.

5. The authors classify CMPs into four categories. Were these categories derived from

PANGO labels, or were other phylogenetic tools employed? Discuss this and make it clear.

*__Response:__* Yes, the four categories are derived from PANGO labels since each category is defined by the CMP's origin and persistence per COVID variant. We have elaborated on the Biological significance of CMPs section to make this clear. Initially these categories were observed based on the presence and absence of certain co-mutations (i.e certain CMPs, such as C3) share mutation positions, yet do not share sub-lineages when the labeled data is superimposed.

6. The longest identified CMP contains 16 positions. How do you interpret the biological feasibility of such large co-mutations occurring together? Are these large CMPs confirmed by experimental evidence or other computational predictions?

*__Response__:* This is a very interesting question. We believe a relevant question is how such a large co-mutation affects the resulting protein or more importantly, its structure. This is a structural biology question that we intend to explore as our next step. All we know is that they are present in significant numbers in the database to be identified by our method, and hence, it should have some biological significance, which we explored in the literature.

7. The proposed method relies heavily on parameter choices such as DSIM(r) and Δ. You should provide more detailed justifications or sensitivity analyses for these choices for reproducibility. You should also make your code and data available.

*__Response:__* We have updated the paper to elaborate on DSIM(r) and the filter threshold. Additionally, the paper now contains a link to our public GitHub repository.

8. How did you determine the statistical significance for the 21 major mutational positions identified by the brute force method? Was there a comparison with known mutational hotspots in the RBD?

*__Response__:* In fact we did that. The total number of point mutations on the RBD sequences are close to 188, but as we count the frequency of their presence in the database, 21 of them show a clear dominance. This fact is also recognized in literature.

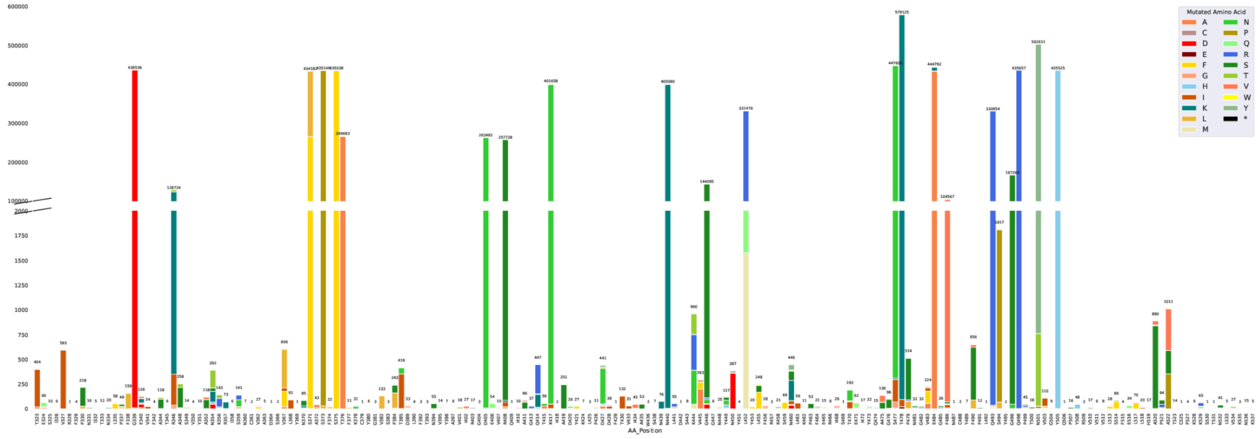

We plotted the above figure where x-axis is the RBD sequence and y-axis represents the number of sequences in the database with mutation at that position. This clearly shows that 21 positions mutated in a significant number. The above Figure is not included in the manuscript, but the summary of our observation is in the Results section, from Comparison against brute-force analysis, pg 10 bottom: "The least frequent among these 21 mutations appeared in 104,567 sequences (14% of the sequences in the database), whereas the highest frequency outside this group was 1,817 sequences (0.0002%), representing a difference of 100-fold."

9. Figure 4 shows clustering of residues based on factor contributions. How did you validate these clusters? Were additional clustering metrics such as Davies-Bouldin Index used to validate these clusters?

*Response:* These clusters are valid in the sense that they are members of a valid component matrix derived from the initial V matrix containing numericized sequence data. We did not use the Davies-Bouldin Index because algorithms using that metric typically expect data with defined coordinates in order to compute their distances. Normally, each coordinate would be plottable along some feature. However, NMF is applied here to discover underlying data features to begin with, and it is not clear how we could apply a Davies-Bouldin Index based algorithm to our matrix.

**Reviewer #3**

In this manuscript, the authors present an approach to discover co-occurring mutations in a sequence database using matrix factorization, more specifically non-negative matrix factorization. While the topic is of interest to the general audience, and NMF has been applied in many areas, I have found the design of the approach has at least a serious flaw, that is "so-called cost function". This function is essentially the count of the different bases between a pair of residues. However, there are many substitutions that are simply synonymous, i.e. multiple codons code the same amino acid. Therefore, the proposed cost function values don't always reflect the difference between two sequences. It's possible you can have exactly the same protein sequence as the reference but completely different scores (of course if all substitutions are synonymous). Also difference scores could be from the same mutation at amino acid residues level. For example, both UUU -> CUU and UUC -> CUU result in Phe->Leu mutation. The score of the first is 1 but the second is 2.

*Responses:*
*Background*: We are analyzing an input matrix that consists of RBD sequences in rows, which are sequences or elements in the database. Since this is not a numerical matrix, we needed a technique to convert each residue into a number that will represent the mutation with respect to the mother sequence (Wuhan). We used the number of mutations of codons in the original nucleic acid sequence for each residue to convert it to an integer, which we called as a "Cost function" for each residue mutation.

Criticism 1: Our codon mutation counts ignore synonymous residues, and hence our numbering scheme is fundamentally wrong, since our study is mainly over residue mutation that matters in protein functionality.

*Response:* Intuitively, our novel Cost function embeds the probability of how long it will take for the residue mutation to take place. Even though synonymous mutation is not effective in protein it is closer to a non-synonymous mutation on an evolutionary pathway, i.e., another nucleic acid mutation may make it a non-synonymous mutation. Since our analysis has some inherent time elements in the evolutionary pathway of the sequences, this is intuitive. The same logic applies to non-synonymous mutations. UUU->CUU mutation may happen faster than the UUC-> CUU mutation and our cost-function measure reflects that.

This does not just occur for single codon mutations because there is statistical support for multi-nucleotide codon mutations (Lucaci, 2021). Consider that the most common Serine codon is AGC and the second most common is TCG (Inouye, 2020). Furthermore, the multi-nucleotide mutation GC→AA is overly represented in the human genome (Harris, 2014, Besenbacher, 2016). If the Serine AGC codon underwent this mutation, then that would change the resultant codon to AAA which encodes the amino acid Lysine. However, that is not possible for the second most common Serine codon TCG; in fact, TCG has a Hamming distance of three relative to the Lysine codon, so Lysine is unreachable for all possible multi-nucleotide mutations with fewer than three positions. Our cost function provides a granular numeralization at the nucleotide base level that can capture nuances that could otherwise be overlooked at the residue level. In fact, multi-nucleotide mutation events can span multiple adjacent codons (Whelan, 2004). Only looking at the amino acids would miss half of the actual mutation. Our cost function can capture multi-nucleotide mutations that do not change an amino acid because they are spread out. See updates to the Mutation matrix generation section in the paper for visual aids for these points. References are available in the manuscript.
